# Heart failure-induced microbial dysbiosis contributes to colonic tumour formation in mice

**DOI:** 10.1093/cvr/cvae038

**Published:** 2024-02-24

**Authors:** Sanne de Wit, Lotte Geerlings, Canxia Shi, Just Dronkers, Elisabeth M Schouten, Gillian Blancke, Vanessa Andries, Tess Yntema, Wouter C Meijers, Debby P Y Koonen, Lars Vereecke, Herman H W Silljé, Joseph-Pierre Aboumsallem, Rudolf A de Boer

**Affiliations:** Department of Cardiology, University Medical Center Groningen, Groningen, 9713 AV, The Netherlands; Department of Cardiology, University Medical Center Groningen, Groningen, 9713 AV, The Netherlands; Department of Cardiology, University Medical Center Groningen, Groningen, 9713 AV, The Netherlands; Thorax Center, Department of Cardiology, Erasmus MC, Cardiovascular Institute, Dr. Molewaterplein 40, Rotterdam, 3015 GD, The Netherlands; Department of Cardiology, University Medical Center Groningen, Groningen, 9713 AV, The Netherlands; Department of Cardiology, University Medical Center Groningen, Groningen, 9713 AV, The Netherlands; Department of Internal Medicine and Paediatrics, Ghent University, 9000, Ghent, Belgium; Host-Microbiota Interaction Lab, VIB Center for Inflammation Research, 9052, Ghent, Belgium; Department of Internal Medicine and Paediatrics, Ghent University, 9000, Ghent, Belgium; Host-Microbiota Interaction Lab, VIB Center for Inflammation Research, 9052, Ghent, Belgium; Department of Paediatrics, University Medical Center Groningen, Groningen, 9713 AV, The Netherlands; Thorax Center, Department of Cardiology, Erasmus MC, Cardiovascular Institute, Dr. Molewaterplein 40, Rotterdam, 3015 GD, The Netherlands; Department of Cardiology, University Medical Center Groningen, Groningen, 9713 AV, The Netherlands; Department of Paediatrics, University Medical Center Groningen, Groningen, 9713 AV, The Netherlands; Department of Internal Medicine and Paediatrics, Ghent University, 9000, Ghent, Belgium; Host-Microbiota Interaction Lab, VIB Center for Inflammation Research, 9052, Ghent, Belgium; Department of Cardiology, University Medical Center Groningen, Groningen, 9713 AV, The Netherlands; Thorax Center, Department of Cardiology, Erasmus MC, Cardiovascular Institute, Dr. Molewaterplein 40, Rotterdam, 3015 GD, The Netherlands; Department of Cardiology, University Medical Center Groningen, Groningen, 9713 AV, The Netherlands; Thorax Center, Department of Cardiology, Erasmus MC, Cardiovascular Institute, Dr. Molewaterplein 40, Rotterdam, 3015 GD, The Netherlands; Department of Cardiology, University Medical Center Groningen, Groningen, 9713 AV, The Netherlands

**Keywords:** Cancer, Faecal microbiota transplantation, Heart failure, Microbiome, Tumour formation

## Abstract

**Aims:**

Heart failure (HF) and cancer are the leading causes of death worldwide. Epidemiological studies revealed that HF patients are prone to develop cancer. Preclinical studies provided some insights into this connection, but the exact mechanisms remain elusive. In colorectal cancer (CRC), gut microbial dysbiosis is linked to cancer progression and recent studies have shown that HF patients display microbial dysbiosis. This current study focussed on the effects of HF-induced microbial dysbiosis on colonic tumour formation.

**Methods and results:**

C57BL/6J mice were subjected to myocardial infarction (MI), with sham surgery as control. After six weeks faeces were collected, processed for 16 s rRNA sequencing, and pooled for faecal microbiota transplantation. CRC tumour growth was provoked in germ-free mice by treating them with Azoxymethane/Dextran sodium sulphate. The CRC mice were transplanted with faeces from MI or sham mice. MI-induced HF resulted in microbial dysbiosis, characterized by a decreased α-diversity and microbial alterations on the genus level, several of which have been associated with CRC. We then performed faecal microbiota transplantation with faeces from HF mice in CRC mice, which resulted in a higher endoscopic disease score and an increase in the number of tumours in CRC mice.

**Conclusion:**

We demonstrated that MI-induced HF contributes to colonic tumour formation by altering the gut microbiota composition, providing a mechanistic explanation for the observed association between HF and increased risk for cancer. Targeting the microbiome may present as a tool to mitigate HF-associated co-morbidities, especially cancer.

## Graphical Abstract

**Graphical Abstract:**
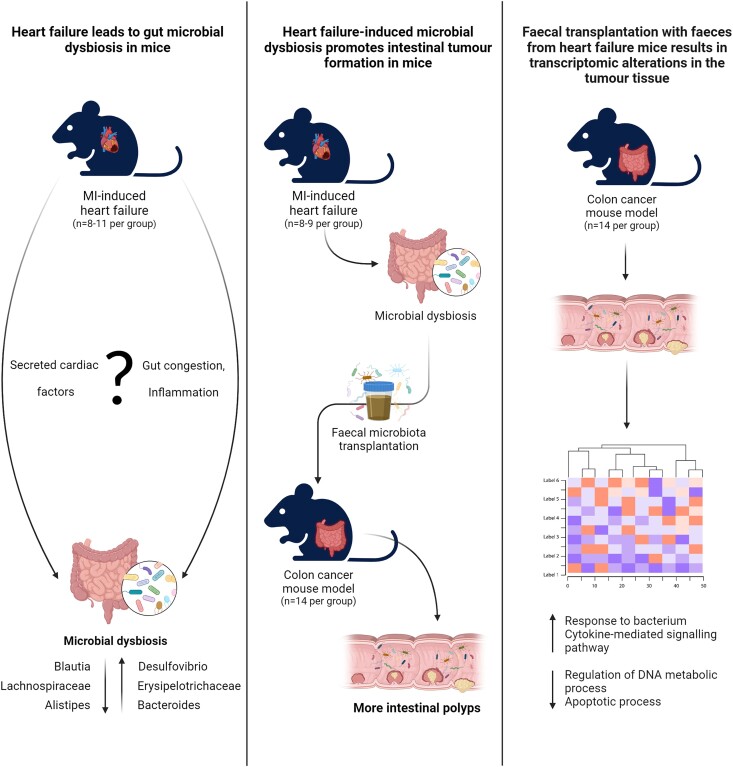

**Time of primary review: 29 days**

**See the editorial comment for this article ‘Reverse cardio-oncology: is heart failuremediated gut dysbiosis the mechanistic driver of colorectal cancer progression?', by A.Y. Rangrez and N. Frey, https://doi.org/10.1093/cvr/cvae051.**

## 1. Introduction

Heart failure (HF) and cancer are the leading causes of death worldwide. Recently, it has become clear that HF and cancer share several pathophysiological characteristics and often coincide in the same patient.^1,2^ Although most attention has gone out to cancer patients with incident cardiovascular disease (CVD) (cardio-oncology), more awareness is given to the increased prevalence of cancer in HF patients (reverse cardio-oncology), including colorectal cancer (CRC)), reported by several independent cohorts recently.^3–5^ In the last few years, several pre-clinical studies have provided mechanistic evidence that HF can stimulate tumour growth. These studies have suggested exciting underlying mechanisms, including the role of cardiac secreted factors and immune cell reprogramming in HF.^6–10^ However, these phenomena likely do not explain the entirety of the bidirectional connections between HF and cancer.

The gut microbiome is emerging as a new potential connector between HF and cancer and has been studied separately in the two diseases. HF patients have an abnormal microbial composition compared to healthy individuals.^11,12^ Increased circulating levels of Trimethylamine N-oxide (TMAO), an essential metabolite produced by gut bacteria, are associated with atherosclerosis and HF^13^ and directly contribute to atherosclerotic plaque formation and HF in mice.^14,15^ In addition, animals models of HF displayed an altered microbial pattern, suggesting that HF itself also induces microbial dysbiosis.^16–18^

The connection between the microbiome and CRC is well established.^19,20^ The microbiome of CRC patients is characterized by a distinctive microbial composition.^21,22^ Microbial dysbiosis may exert tumourigenic properties as preclinical studies showed that faecal transplantation with microbiota from CRC patients stimulated tumour formation in mice,^23,24^ indicating a direct effect of the abnormal microbiome on tumour development.

Considering its role in both diseases, we hypothesized that HF-induced gut microbial dysbiosis could stimulate colorectal tumour formation. Therefore, we aimed to assess the direct effects of HF on the gut microbiota composition, and determine the effect of HF-induced microbial dysbiosis tumour formation in CRC, using a faecal microbiota transplantation (FMT) approach.

## 2. Materials and methods

### 2.1 Animals

All studies were performed in accordance with the Guide for the Care and Use of Laboratory Animals published by the US National Institutes of Health. All experimental procedures were approved by the Animal Ethics Committee (CCD_AVD10500201583, University of Groningen, the Netherlands, LA_1400637, Ghent University, Belgium). HF studies were performed in male 8–10 weeks old C57BL/6J mice (Jackson Laboratory, #000664). The mice were individually housed in conventional housing, under controlled conditions (12 h light–dark cycle) with *ad libitum* access to food and water. Mice were weighed twice a week and food and water intake was monitored weekly. The FMT study was performed in 6–8 weeks old germ-free C57BL/6 mice. C57BL/6 germ-free mice were born and raised at the Ghent Germ-free and Gnotobiotic mouse facility (Ghent University, LA2400637/LA1400637). They were maintained in a sterile environment under controlled conditions (10 h light–dark cycle) with *ad libitum* access to water and diet (2018S, Envigo, USA). Germ-free mice were housed and bred in conventional cages in positive-pressure flexible film isolators (North Kent Plastics). One week before the start of the experiment, germ-free mice were transferred to individually ventilated Isocage-P cages (positive pressure Isocages Techniplast) and housed in groups of 2–4. All handling of the germ-free animals was performed under sterile conditions, working in a laminar flow cabinet (Avantor, VWR international).

### 2.2 Experimental protocol

To study the effects of HF on the gut microbiome 8 weeks old C57BL/6J mice were randomized for myocardial infarction (MI), transverse aortic constriction (TAC) or sham surgery (sham *n* = 11, MI *n* = 8, TAC *n* = 9). Five weeks after surgery cardiac parameters were measured using echocardiography. Six weeks after surgery the mice were euthanized under isoflurane anaesthesia by excising the heart. Faecal samples were collected and snap frozen for 16 s RNA gene sequencing, and tissues were collected and snap frozen for molecular analyses or fixated in 4% formalin and paraffin embedded for histochemical purposes. To study the role of HF-induced microbial dysbiosis on CRC tumour formation FMT was performed. As donor mice, 8–10 weeks old C57BL/6J mice were subjected to MI or sham surgery and the same experimental setup was used as described above. Six weeks after surgery, caecum content and faeces were collected when the mice were euthanized, and processed and pooled for faecal transplantation. Three mice were excluded as donor mice because they did not show a decrease in EF or an increase in ANP levels and therefore did not meet the a priori agreed criteria for the HF phenotype (sham *n* = 8, MI *n* = 9). As recipient mice, 6–8 weeks old germ-free mice were transplanted with faeces of either MI mice or sham mice (FMT-sham *n* = 14, FMT-MI *n* = 14). Mice were inoculated three times in the first week and then every other week for 8 weeks. The tumour model was induced two weeks after the first inoculation by treating the mice with Azoxymethane (AOM)/Dextran sodium sulphate (DSS). Disease progression was monitored using the disease severity score and endoscopy. Ten weeks after the first inoculation mice were euthanized by CO_2_ exposure. The colon was isolated, measured and dissected longitudinally. Tumour count and size were determined macroscopically. The tumour load was calculated, assuming a hemispherical form of the polyps; (tumour count*(2/3*3.14*((0.5*tumour size)^3)). From three sham mice, the pictures failed; therefore tumour size and load were not determined from those mice. Tissues were collected and snap frozen for molecular analyses or fixated in 4% formalin and paraffin embedded for histochemical purposes.

### 2.3 Heart failure surgery

For all surgical procedures mice were intubated and mechanically ventilated with a 2% isoflurane/oxygen mixture using a rodent ventilator (Harvard Minivent, model 845). Body temperature was maintained at 37°C. Preoperatively, all mice received carprofen (5.0 mg/kg) for analgesic purposes. MI was inflicted by permanent ligation of the left anterior descending coronary artery using 6-0 perma-hand silk suture, through an incision in the fourth intercostal space. After tying the ligature, the heart was inspected for paleness indicative for impaired blood flow. Ribs were closed with 5-0 prolene and the skin layer was sutured with 5-0 vicryl. TAC was inflicted by placing an 0.56 mm rubber O-ring (Apple rubber) around the ascending aorta, through an incision in the second intercostal space as described by Melleby et al.^25^ Sham-operated animals underwent the same procedure, except the placement of the ligature, or placement of the O-ring.

### 2.4 Faecal microbiota transplantation

Faecal and caecal content from the donor mice were collected when the mice were euthanized (6 weeks after HF surgery). The faecal and caecal content was snap frozen in liquid nitrogen and stored at −80°C. Faecal and caecal content were homeogenized (Tissuelyser LT- Qiagen) at 30 Hz for 3 min and dissolved in 1× PBS in an anaerobic chamber (Ruskinn, Concept 400, Baker Company). Faecal slurry was aliquoted and stored at −80°C. FMT was performed on 3 consecutive days in the first week and every other week for the subsequent 8 weeks. The faecal slurry was transplanted via oral gavage under sterile conditions in a laminar flow hood (Avantor, VWR international).

### 2.5 AOM/DSS treatment

Tumour development was induced by treating the mice with AOM (Santa Cruz Biotechnology, SC-358746) and DSS (MP Biomedicals, 9011-18-1). AOM was injected intra-peritoneally at a dose of 10 mg/kg, 2 weeks after the first faecal transplant inoculation. One week after AOM injection, 2% DSS was administered via drinking water for 5 days. The DSS treatment was repeated twice with a 16-day recovery period. The third round of DSS was performed for 3 days.

### 2.6 Echo

Five weeks after HF surgery, M-mode and 2-dimentional echocardiography was performed to assess cardiac structure and function using the Vevo 3100 system (FuJiFLIM VisualSonics, Toronto, Canada), equipped with 40-MHz MXX550D linear array transducer. Mice were anaesthetized with 2–2.5% isoflurane in oxygen during the procedure. Body temperature was maintained at 37°C. The Vevo LAB software version 5.5.0 (FuJiFLIM VisualSonics) was used to measure echocardiographic parameters.

### 2.7 Endoscopy

High-resolution mouse endoscopy was performed, as previously described,^26^ using a Coloview endoscopic system (Karl Storz). Mice were anaesthetized with 2–2.5% isoflurane in oxygen during the procedure. Evaluation and scoring of the colonic architecture was done according to the endoscopic colitis (MEICS) scoring reported by Becker *et al*. focussing on colonic wall thickness, changes in vascular pattern (bleeding), fibrin deposition, granularity and stool consistency.^26^

### 2.8 Disease severity score

During DSS treatment mice were monitored and scored for bodyweight changes, faecal consistency and blood in the faeces using the hemoCARE Guaiac test. Body weight score was defined as 0, 0–5% body weight loss; 1, 5–10%; 2, 10–15%; 3, 15–20%; and 4, > 20%. For stool consistency, a score of 0 was assigned for well-formed stools, a score of 2 was assigned for pasty and semi formed stools and a score of 4 was assigned for liquid stools. For bleeding, a score of 0 was assigned for no blood, a score of 2 was assigned for positive Hemoccult and a score of 4 was assigned for gross bleeding. The disease severity score was calculated as the average of all categories combined.

### 2.9 Blood collection

Blood was collected via cardiac puncture in EDTA tubes. Blood was centrifuged at 1500xg for 10 min at room temperature. The upper plasma layer was transferred to a new tube and stored at −80°C.

### 2.10 DNA isolation

Fresh stool samples were collected when the mice were euthanized and were immediately snap frozen in liquid nitrogen and stored at −80°C. To determine the overlap in the faecal content that was used for the FMT and the faeces of the transplanted mice, DNA was isolated from the pooled faecal/caecal samples from the donor mice (four technical replicates per group). DNA was isolated using a phenol-chloroform extraction technique with mechanical disruption (bead-beating). DNA was precipitated in 1 × TE buffer. The presence of bacterial DNA was verified by PCR and gel electrophoresis using universal primer set for the V4 region (515F, 806R) (see Supplementary material online, *Table S3*). PCR products were loaded on a 1.5% agarose gel with the gene ruler DNA ladder mix.

### 2.11 RNA gene sequencing

16S RNA gene sequencing amplification, indexing and Illumina MiSeq sequencing (2 × 250 base pairs (bp)) was performed by Novogene Co., Ltd. The hypervariable V4 region was amplified using the 515F (GTGCCAGCMGCCGCGGTAA) and 806R primers (GGACTACHVGGGTWTCTAAT). Subsequent analysis was performed through the Qiime2 pipeline (https://doi.org/10.1038/s41587-019-0209-9) and visualisations were made in Rstudio (v2022.07.02) with the packages ggplot2 (v3.4.4; DOI: 10.1007/978-3-319-24277-4), ggsignif (v0.6.4)(DOI: 10.31234/osf.io/7awm6), ggpubr (v.0.6.0 Kassambara A (2023). ggpubr: ‘ggplot2’ Based Publication Ready Plots. R package version 0.6.0, https://rpkgs.datanovia.com/ggpubr/.), ggrepel (v0.9.3, https://CRAN.R-project.org/package=ggrepel), mia (v1.2.7)(DOI: 10.18129/B9.bioc.mia), miaViz (v1.2.1) (DOI: 10.18129/B9.bioc.miaViz), scater (v1.22.0, DOI: 10.18129/B9.bioc.scater), vegan (v2.6-4, https://doi.org/10.1111/j.1654-1103.2003.tb02228.x) with the kruskal.test function. Rarefaction was performed to normalize for library size. Significant taxa on the genus level were identified using MaAslin2 (v1.8.0, 10.1371/journal.pcbi.1009442), a package determining multivariate association based on linear models.^27^ Default MaAslin2 parameters were applied; maximum percentage of samples NA in metadata 10%, *P* value *P* < 0.05 and all *P* values were adjusted for multiple comparisons using the Benjamini Hochberg correction with a FDR cut-off of q < 0.25%.^27^ All 16S rRNA gene sequencing data used in this paper will be shared by the lead contact upon request.

### 2.12 RNA sequencing

RNA was isolated from snap frozen colon tissue using TRI reagent (Sigma-Aldrich, St Louis, MO). RNA samples were characterized by UV-Vis spectrophotometry (Nanodrop2000c, Thermo Fisher), RNA integrity was assessed on a Fragment Analyzer System using the DNF-471 RNA Kit (15 nt) (Agilent). Sequencing-ready libraries were produced using a QuantSeq 3’ mRNA-Seq Library Prep Kit REV for Illumina (015UG009V0241) following standard procedures, as outlined in the respective UserGuide:https://www.lexogen.com/wp-content/uploads/2018/08/015UG009V0241_QuantSeq_Illumina.pdf

Indexed library preparation was performed to allow for multiplexed sequencing. For library preparation, 200 ng of (provided) RNA samples were used as an input. Prepared libraries were quality controlled on a Bioanalyzer device (Agilent), using the HS-DNA assay. Concentration of obtained libraries was quantified using a Qubit dsDNA HS assay (Thermo Fisher). A sequencing-ready pool of indexed libraries was prepared according to these quantifications. Sequencing was performed on an Illumina NextSeq2000 with SR100 read mode at Lexogen GmbH.

The ShinyGO enrichment tool (version 0.77) (http://bioinformatics.sdstate.edu/go/ (accessed on 31 November 2023) was used for exploring enrichment in Gene Ontology (GO) categories for biological processes using the lists of up- and down-regulated genes and proteins (*P* < 0.05).^28^ Biological processes with false discovery rate (FDR) < 0.05 were considered significant.

### 2.13 Caecal SCFA measurements

Caecal SCFA concentrations were measured as previously described.^29^ In brief, the caecum was thawed, internal standard (0.5 mg/mL 2-ethylbutyric acid solution) was added and the caecum was homeogenized using beat-beating (4°C, 60 s, 6000 rpm). SCFAs were extracted with 2 mL diethyl ether (MERCK, #60-29-7). Derivatization was performed overnight with 500 µL of the supernatant and 50 µL of N-tert-Butyldimethylsilyl-N-methyltrifluoroacetamide (MTBSTFA)(Sigma-Aldrich, #77377-52-7), at room temperature. SCFA concentrations were measured on an Agilent 5975C series GC/MS (Agilent Technologies), equipped with a ZB-1 column (Phenomenex, Torrance, CA, USA). Mass spectrometry analysis was performed by electron ionization. Ions monitored were m/z 117 for acetate, m/z 131 for propionate, m/z 145 for butyrate and m/z 221 for 4-phenol butyric acid.

### 2.14 Real time PCR

RNA was isolated from snap frozen LV and colon tissue using TRI reagent (Sigma-Aldrich, St Louis, MO) and cDNA was made using the QuantiTect® Reverse Transcription Kit (Qiagen, Germany) according to the manufacturer’s protocol. qPCR was performed using a Bio-Rad CFX384 Real-Time PCR system (Bio-Rad, CA, USA) using SYBR Green dye. Peptidyl-prolyl cis-trans isomerase (Ppia) or 36B4 (Rplp0, ribosomal protein large P0) were used as a reference to correct for the measured mRNA expression. Primer sequences are shown in *Table 1*. For TNFα one outlier was excluded in the MI group. For IL6 in several samples IL6 levels were below threshold and could not be measured.

**Table 1:** Primer sequences

Gene	Sequence
Nppa	Fwd: GCTTCCAGGCCATATTGGAG Rev: GGTGGTCTAGCAGGTTCTTG
Ppia	Fwd: CCACCGTGTTCTTCGACATC Rev: AGTGCTCAGAGCTCGAAAGT
ZO-1	Fwd: GGGCCATCTCAACTCCTGTA Rev: AGAAGGGCTGACGGGTAAAT
Occludin	Fwd: ACTATGCGGAAAGAGTTGACAG Rev: GTCATCCACACTCAAGGTCAG
Claudin-3	Fwd: CCTGTGGATGAACTGCGTG Rev: GTAGTCCTTGCGGTCGTAG
MUC2	Fwd: GTAGTGGAGATTGTGCCGCT Rev: CAGGAACACGCACAGGTTTG
TNF-α	Fwd: AAACCACCAAGTGGAGGAGC Rev: ACAAGGTACAACCCATCGGC
IL-6	Fwd: TCCCAACAGACCTGTCTATAC Rev: CAGAATTGCCATTGCACAACTC
36B4	Fwd: AAGCGCGTCCTGGCATTGTC Rev: GCAGCCGCAAATGCAGATGG
515F	GTG-CCA-GCM-GCC-GCG-GTA-A
806r	GGA-CTA-CHV-GGG-TWT-CTA-AT

### 2.15 Histology

Paraffin embedded colonic tissues were sectioned at 4 µm. sections were deparaffinated and dehydrated, and incubated with antigen retrieval for 15 min. For immunohistochemistry, endogenous peroxidase was blocked with 3% H_2_O_2_. Sections were incubated with primary rabbit anti Ki67 (AB16667, Abcam) for 60 min, secondary goat anti-rabbit HRP (P0448, DAKO) for 30 min and tertiary rabbit anti-goat HRP (P0449, DAKO) for 30 min at room temperature. The slides were counterstained with haematoxylin, mounted and imaged using a Nanozoomer 2.0 HT (Hamamatsu, Japan). The percentage of Ki67 positive cells was determined in tumour tissue using ImageJ. The Ki67 staining failed in two samples, therefore the *N* = 13 per group. For the immunofluorescence staining, the sections were stained with either Claudin-3 (#34-1700, Thermo Fisher) or ZO-1 (#61-7300, Thermo Fisher) as primary antibody and Dk anti Rb 555 (#A31572, Thermo Fisher) as secondary antibody. The sections were counterstained with WGA-FITC (Sigma, L4895-2 mg), mounted with Vectashield mounting medium with DAPI (Vector Labs, H-1200) and imaged using a Nanozoomer 2.0 HT (Hamamatsu, Japan). The images were visualized using OLYMPUS-OliVIA.

### 2.16 Statistical analyses

Data are represented as mean ± standard deviation (SD). Outliers were determined using the ROUT method. Normal (Gaussian) distribution was determined using the Shapiro-Wilk test. Student-t test was used to determine statistical significance for normally distributed data, or Mann–Whitney *U* test for non-normally distributed data or data with small group sizes. For parameters that were measured over time two-way ANOVA with Sidak’s multiple comparisons test was performed. Differences were considered significant at *P* < 0.05. Statistical tests were performed in GraphPad Prism 9.1.0 (GraphPad Software, La Jolla, CA, USA) or Rstudio (version v2022.07.02) for the sequencing data.

## 3. Results

### 3.1 Myocardial infarction-induced HF alters the gut microbial composition

**Figure 1:**
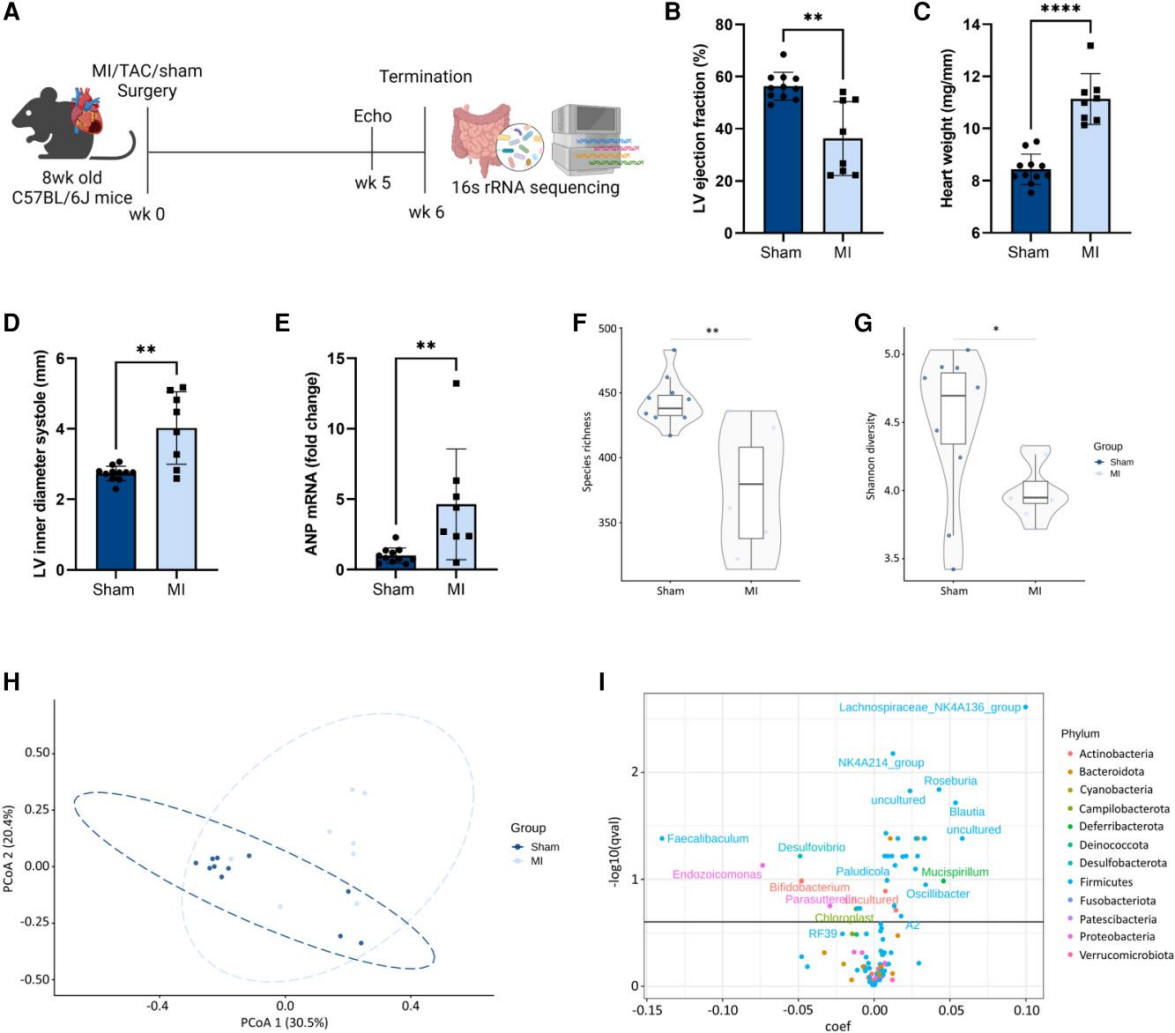
Effects of MI-induced HF on the gut microbial composition. (*A*) Experimental setup (*B*). Left ventricular ejection fraction (LVEF) (*C*). Heart weight normalized for tibia length (*D*). Left ventricular inner diameter systole (LVIDs) (*E*). Relative *natriuretic peptide A (Nppa)* expression in LV, normalized for *peptidyl-prolyl cis-trans isomerase (Ppia)*. α-Diversity analysis represented by (*F*). the number of observed species and (*G*). Shannon diversity index. (*H*) β-diversity represented by principal coordinates analysis (PCoA) of Bray–Curtis dissimilarity distances of bacterial composition. (*I*) Volcano plot showing differentially abundant bacteria on the genus level, represented by −log10(q-value) (bacterial genera are coloured according to the phylum they belong to). The data from *Figure 1B–D* have previously been published by Aboumsallem *et al.*^30^ Data are represented as mean ± SD. Statistical significance was determined using Mann–Whitney *U* test (B-G) or MaAslin2, FDR q < 0.25% (*I*). *P* < 0.05 was considered as statistically significant. * *P* < 0.05, ** *P* < 0.01, **** *P* < 0.0001. Sham *n* = 11, MI *n* = 8.

The effect of HF on the gut microbial composition was investigated in mice subjected to either MI, TAC, or sham surgery (*Figure 1A*). MI-induced HF resulted in a 20% decrease in left ventricular (LV) ejection fraction (EF) (*Figure 1B*), as well as an increase in heart weight, LV inner diameter, and a four-fold increase in atrial natriuretic peptide (ANP, encoded by the *NPPA* gene) expression (*Figure 1C–E*). No differences were observed in body weight and food intake between sham and HF mice (see Supplementary material online, *Figure S1A and B*). Faecal samples of MI mice showed a decrease in the number of bacterial species and Shannon diversity index, indicative of a decreased microbial diversity (*Figure 1F* and *G*). Principle coordinate analysis (PCoA) revealed that most of the MI mice were clustered separately form the sham mice, although some overlap occurred (*Figure 1H*). At the genus level, MI-induced HF was associated with 36 differentially abundant bacteria. *Lachnospiraceae* NK4A136 and *Oscillospiraceae* NK4A214 were the most significantly decreased genera (*Figure 1I* and Supplementary material online, Files*S1* and *S2*). In addition, the genera *Blautia*, *Alistipes*, *Anaerotruncus*, and *Intestimonas* were decreased, among others (*Figure 1I* and Supplementary material online, Files*S1* and *S2*). The genera *Faecalibaculum* and *Desulfovibrio* were the most significantly increased in the MI mice (*Figure 1I* and Supplementary material online, Files*S1* and *S2*). Several of the bacteria that were decreased in the faeces of the MI mice are known to be involved in short-chain fatty acid (SCFA) production.^31,32^ In line with the decrease in SCFA-producing bacteria, caecal butyrate levels were decreased in HF mice (see Supplementary material online, *Figure S1C*). It is hypothesized that HF might affect intestinal barrier function and induce low-grade inflammation in the gut.^33^ In the colon of HF mice no changes were observed in gene expression levels of barrier proteins, *Zonula occludens-1 (ZO-1)*, *occludin, claudin-3* and *mucin-2 (MUC2)*, as well as inflammatory markers, tumour necrosis factor-α (TNFα) and interleukin-6 (IL6) (see Supplementary material online, *Figure S1D–H*).

Comparable to MI, TAC-induced HF resulted in a decreased EF and an increase in heart weight LV inner diameter and ANP expression (see Supplementary material online, *Figure S2A–D*). However, no differences in species richness or Shannon diversity were observed between sham and TAC mice (see Supplementary material online, *Figure S2E* and *F*). In addition, TAC and sham mice were largely clustered together in the PCoA analysis (see Supplementary material online, *Figure S2G*). On the genus level 9 significantly different bacteria were observed between TAC and sham (see Supplementary material online, *Figure S2H*, Supplementary material online, File*S3*). The decrease in the genera *Lachnospiraceae* NK4A136, *Oscillospiraceae* NK4A214, *Lachnospiraceae* GCA.900066575, *Blautia*, *Peptococcus*, and *Alistipes* were comparable between the TAC and MI mice (*Figure 1I*, Supplementary material online, *Figure S2H*, Supplementary material online, Files*S1*–S3).

### 3.2 Transplant of faeces from mice with MI-induced HF accelerates tumour formation in a CRC mouse model

**Figure 2:**
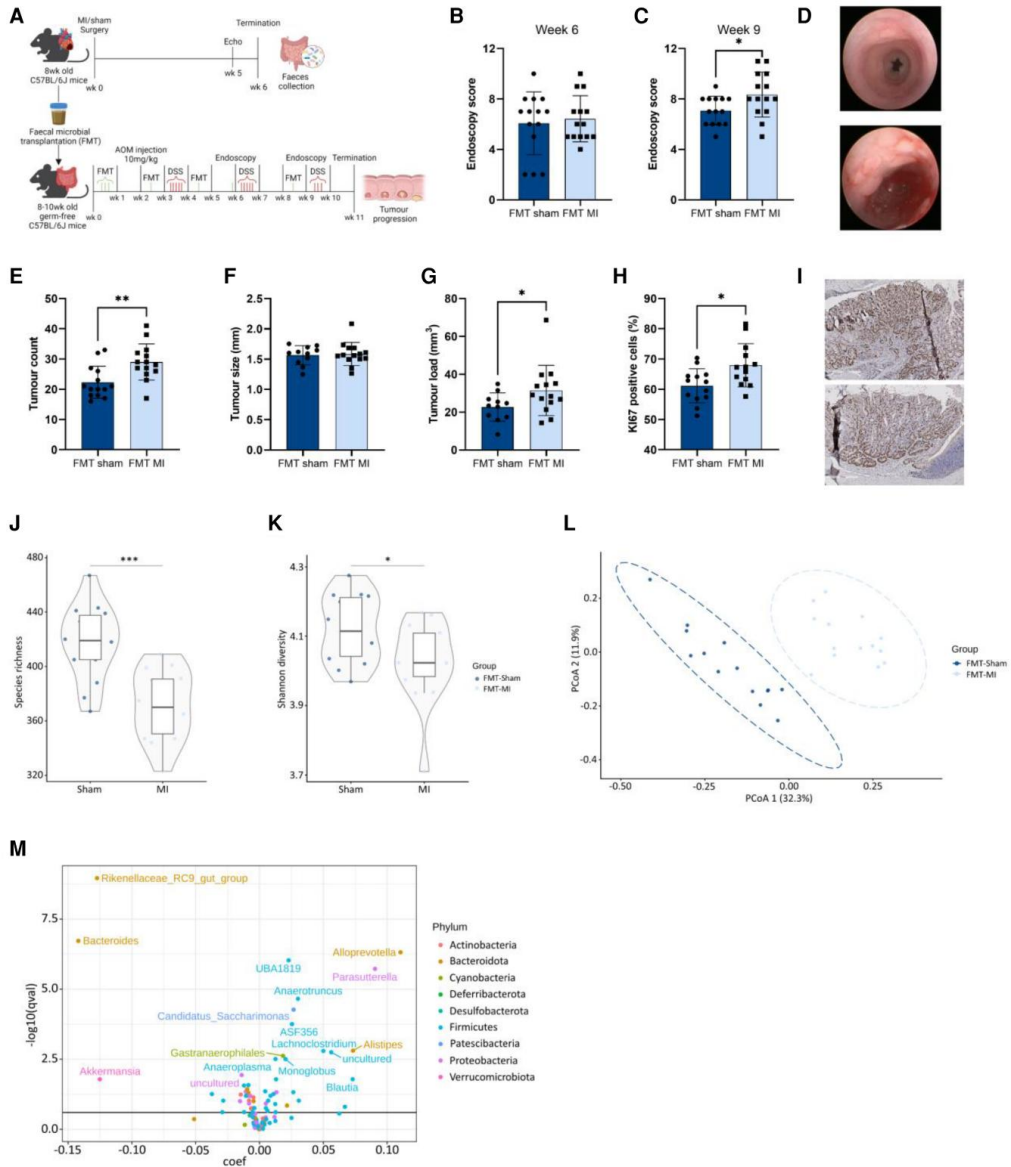
The effect of microbial transplantation from MI mice on colonic tumour formation. (*A*) Experimental setup. Colonic pathology quantified with the murine endoscopic index of colitis severity (MEICS) score at (*B*). 6 weeks and (*C*). 9 weeks. (*D*) Representative pictures of the endoscopy at 6 weeks (top) and 9 weeks (bottom). (*E*) Number of colonic tumours *(F)*. Average size of the tumours (*G*). Calculated tumour load (*H*). Percentage of Ki67 positive cells (*I*). Representative pictures of the Ki67 staining; top: FMT-MI, bottom: FMT-sham, scale bar: 300 µm. α-Diversity analysis represented by (*J*). the number of observed species and (*K*). Shannon diversity index. (*L*) β-diversity represented by principal coordinates analysis (PCoA) of Bray–Curtis dissimilarity distances of bacterial composition. (*M*) Volcano plot showing differentially abundant bacteria on the genus level. Data are represented as mean ± SD. Statistical significance was determined using Student’s *t*-test (B–F, H) or Mann–Whitney *U* test for non-normally distributed data (G, J–K). Significantly differential genera were determined using MaAslin2, FDR q < 0.25% (M). *P* < 0.05 was considered as statistically significant. * *P* < 0.05, ** *P* < 0.01, **** *P* < 0.0001. B-E, J-M: *n* = 14 per group, F-G: FMT-sham *n* = 11, FMT-MI *n* = 14; H: *n* = 13 per group.

To investigate whether gut microbial dysbiosis caused by MI-induced HF could directly trigger intestinal tumour growth, faecal samples from MI mice or control mice were transplanted into germ-free mice. Faecal and caecal content from the donor mice were collected six weeks after the HF surgery, when the mice were euthanized, and were processed anaerobically for faecal transplantation. After the first FMT, the germ free mice were exposed to the AOM/DSS to induce CRC (*Figure 2A*). Donor mice subjected to MI showed a clear HF phenotype characterized by a decrease in EF and an increase in LV diameters, heart weight and *Nppa* expression, 6 weeks after MI surgery (see Supplementary material online, *Figure S3A–D*). MI and sham mice had similar body weight changes and food and water intake during the entire experiment (see Supplementary material online, *Figure S3E* and *F*).

To determine the microbiota composition of the faecal content that was used for the FMT, the pooled and processed faecal/caecal content from the donor mice was sequenced, using four technical replicates. The α-diversity was lower in the pooled donor faeces and the PCoA analysis revealed that the pooled faecal content of the MI mice was clustered separately from the sham mice (see Supplementary material online, *Figure S3H* and *I*). At the genus level, 76 bacteria were detected in the pooled faeces from the sham mice and 74 bacteria in the pooled faeces from the MI mice. Fifty-eight of the detected bacteria in the donor-sham mice were comparable to bacteria detected in the sham mice (76.3%) (see Supplementary material online, File*S4*). Fifty-seven of the detected bacteria in the donor MI mice were comparable to bacteria detected in the MI mice (77%) (see Supplementary material online, File*S4*). MI-induced HF was associated with 47 differentially abundant bacteria. 18 of the differentially abundant bacteria were comparable between the pooled donor MI faeces and faeces from the MI mice (see Supplementary material online, *Figure S3K*, Supplementary material online, File*S4*). This included a decrease in the genera *Lachnospiraceae* NK4A136, *Blautia*, *Alistipes*, *Anaerotruncus*, and *Intestimonas* and an increase in *Desulfovibrio* and *Candidatus Arthromitus*. Discrepancies between the donor MI samples and the MI samples were found in three genera.

In the CRC mice no differences were observed in body weight changes or disease severity score during DSS treatments between mice transplanted with faeces from MI mice or mice transplanted with faeces from sham mice (see Supplementary material online, *Figure S4A* and *B*). Cardiac function of the germ-free mice was not assessed in this model, as previous studies have described that cardiac function and heart weight are not altered in germ-free mice.^34,35^ Six weeks after FMT, no differences were observed in murine endoscopic index of colitis severity (MEICS) scoring between the experimental groups (*Figure 2B*). At nine weeks however, mice transplanted with HF faeces had a higher MEICS score than mice transplanted with faeces from sham mice (*Figure 2C*). The colonic architecture of the mice transplanted with faeces from MI mice was characterized by increased colonic wall thickness, more mucosal bleeding and deposition of fibrin in the lumen, and worse stool consistency (*Figure 2B–D*). No differences in colon length were observed between mice transplanted with sham faeces and mice transplanted with HF faeces (see Supplementary material online, *Figure S4C*). Mice transplanted with faeces from MI mice showed a 30% increase in colonic tumour count (22.29 ± 5.3 vs. 29.0 ± 6.0, *P* = 0.0043) (*Figure 2E*), whereas there was no difference in the size of tumours between the groups (*Figure 2F*). Altogether, FMT with faeces from MI mice led to a significant increase in overall tumour load (22.65 ± 5.6 vs. 31.43 ± 13.3, *P* = 0.041) (*Figure 2G*). In addition, the number of Ki67 positive cells was higher in tumour tissue of mice transplanted with HF faeces (61.14 ± 5.6 vs. 67.95 ± 7.1, *P* = 0.0125) (*Figure 2H* and *I*).

The faecal microbiome from mice transplanted with HF faeces was characterized by a decrease in α-diversity (*Figure 2J* and *K*). PCoA analysis revealed a clear separation between the faecal content of the mice transplanted with faeces from MI mice and mice transplanted with faeces from control mice (*Figure 2L*). In the mice transplanted with faeces from control mice, 121 bacteria were detected on the genus level, of which 65 overlapped with bacteria found in the donor-control mice (see Supplementary material online, Files*S4* and *S5*). In the mice transplanted with faeces from MI mice, 120 bacteria were detected on the genus level, of which 65 overlapped with bacteria found in the pooled faecal samples of the donor MI mice (see Supplementary material online, Files*S4* and *S5*). On the genus level, transplantation with faeces from MI mice was associated with 50 differentially abundant bacteria (*Figure 2M*, Supplementary material online, Files*S5* and *S6*). 14 of the differentially abundant bacteria were comparable with the pooled donor samples (see Supplementary material online, Files*S4* and *S5*). This included a decrease in the genera *Blautia*, *Alistipes*, *Anaerotruncus*, and *Intestimonas* and an increase in *Enterorhabdus*, *Bacteroides*, and *Clostridia* UCG.014 (*Figure 2M*, Supplementary material online, Files*S5* and *S6*). Discrepancies between the donor mice and the mice that received the faecal transplant we observed in eight genera, including *Erysipelotrichaceae* and *Lactobacillus* (*Figure 2M*, Supplementary material online, Files*S5* and *S6*).

### 3.3 Tumour tissue transcriptomics reveals enhanced cytokine-related mechanisms and decreased apoptotic mechanisms in mice transplanted with faeces from MI mice

**Figure 3:**
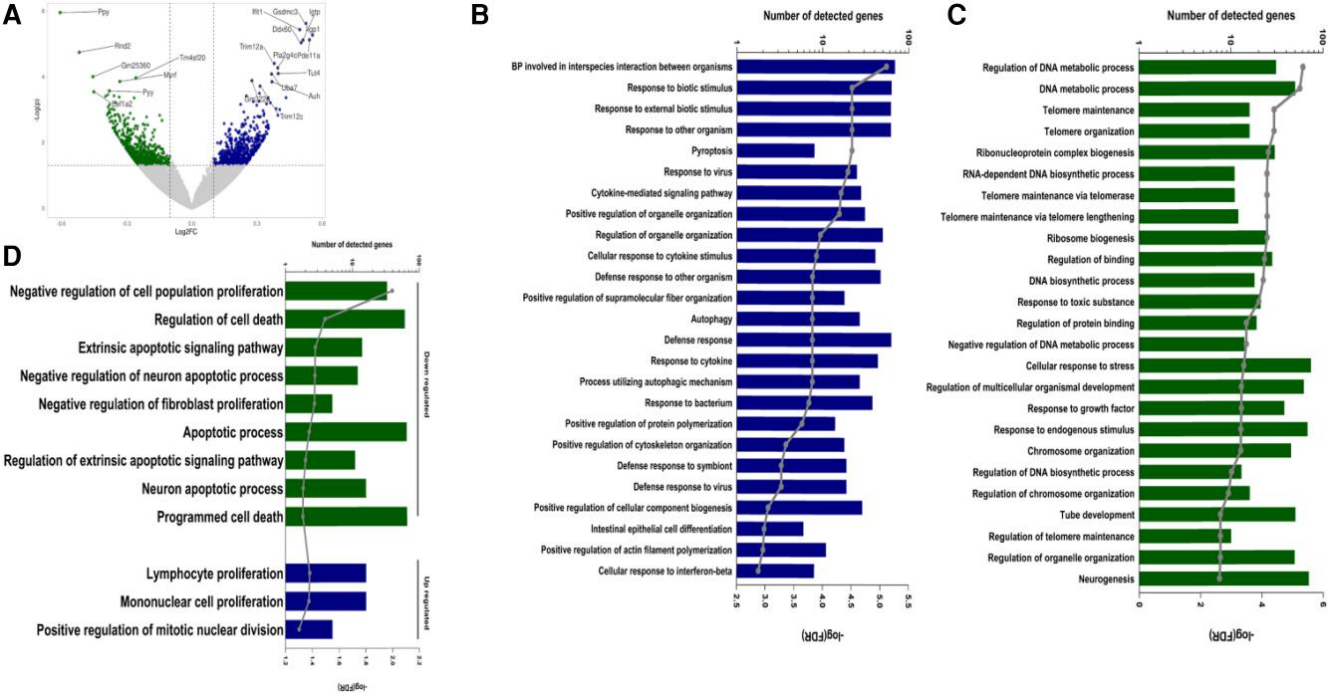
Differential gene expression analysis and enriched biological processes in colon tissue of CRC mice transplanted with faeces from HF mice. (*A*) Volcano plot showing differentially expressed gene and significance (represented by −log(*P*-value)). (*B*) Bar graph representing the number of detected genes (from the list of up-regulated genes in FMT-MI) observed in specific biological processes (only the top 25 processes are shown, based on significance (FDR, presented by the dotted line)). (*C*) Bar graph representing the number of detected genes (from the list of down-regulated genes in FMT-MI) observed in specific biological processes (only the top 25 processes are shown, based on significance (FDR, presented by the dotted line)). (*D*) Bar graph representing the number of detected genes (from the list of down-regulated and upregulated genes in FMT-MI) observed in specific biological processes (only with the terms proliferation, cell death, apoptosis, division). Significance, FDR is represented by grey dots)). *N* = 8 per group.

The volcano plot (*Figure 3A*) represents all differentially expressed genes (DEGs) in tumour tissues from FMT sham and FMT-MI. The gasdermin C3 (Gsdmc3) gene was the top significantly up-regulated gene in FMT-MI (log2FC = 0.525, padj = 0.015), followed by interferon-induced protein with tetratricopeptide repeats 1 (Ifit1) (log2FC = 0.497, padj = 0.0159) and interferon-inducible GTPase 1 (Iigp1) (log2FC = 0.555, padj = 0.0165) (*Figure 3A*). The pancreatic polypeptide (Ppy) gene was the top significantly down-regulated gene in FMT-MI tumour tissues (log2(fold change) (log2FC) = −0.605, padj = 0.0144), followed by the Rho family GTPase 2 (Rnd2) gene (log2FC = −0.518, padj = 0.029) (*Figure 3A*). The complete list of differentially expressed genes is provided in Supplementary material online, File*S7*.

Biological process involved in interspecies interaction between organisms (FDR = 7.8 × 10^−6^, 69 DEG) was the most significantly enriched biological process in FMT-MI. Additionally, the response to bacterium (FDR = 0.00017, 38 DEG) and the defence response to symbiont (FDR = 0.00052, 19 DEG) were significantly enriched (*Figure 3B*). Furthermore, several defence-related biological processes were also enriched among the top 25 most significant biological processes *Figure 3B*). This includes cytokine-mediated signalling pathways (FDR = 4.8 × 10^−5^, 28 DEG) and Cellular response to cytokine stimulus (FDR = 0.00013, 41 DEG) (*Figure 3B*). The complete list of enriched biological processes is provided in Supplementary material online, File*S8*.

Based on the list of down-regulated genes, the regulation of DNA metabolic process was the most significantly enriched biological process (FDR = 4.4 × 10^−6^, 31 DEG) (*Figure 3C*). The top 25 most significant down-regulated biological processes included telomere maintenance (FDR = 3.91 × 10^−^5, 16 DEG) and telomere organization (FDR = 3.91 × 10^−5^, 16 DEG) (*Figure 3C*). The complete list of enriched biological processes in provided in Supplementary material online, File*S9*.

The enrichment analyses revealed that several biological processes related to cell proliferation and apoptosis/cell death were enriched. This includes the mononuclear cell proliferation (FDR = 0.042, 16 DEG) (*Figure 3D*), which was enriched as up-regulated. On the other hand, apoptotic process (FDR = 0.0418, 65 DEG), and programmed cell death (FDR = 0.0467, 66 DEG) were enriched as down-regulated (*Figure 3D*).

## 4. Discussion

In this study, we have explored and discovered a new connection demonstrating that MI-induced HF may contribute to the development of colonic tumours, by changing the gut microbial composition in mice. First, we demonstrate that HF induced by MI leads to a shift in microbial diversity (dysbiosis). Second, when we transplanted microbiota, using faeces from mice with HF into a CRC mouse model, we observed an increase in tumour formation. Third, we show that the microbial content in HF comprises several bacteria compatible with increased tumour formation. Collectively, these findings may suggest a new potential explanation explaining why patients with HF have a higher incidence of cancer.

Over the last years, the role of the microbiome in heart disease has received a growing interest. Patients with heart disease have a distinct microbiota composition compared to healthy controls.^11,36–42^ Several of the differentially expressed bacterial taxa found in our data were comparable to the changes that are observed in patients with HF and CVD, including a decrease in *Blautia*, *Lachnospiraceae*, *Roseburia*, *and Alistipes.*^11,36,37,40–42^ Some contradictory findings were also observed, as the genus *Bifidobacterium* is often reported to be decreased in patients with heart disease and was increased in our MI mice.^37,41^ Interestingly, TAC-induced HF did not result in major alterations in the microbiota composition. Previous preclinical studies have published some confirmatory and some discrepant findings on the effects of MI or TAC on the microbial composition, which may be attributed to differences in experimental setup and sequencing methods.^16–18,43,44^ It would be interesting to further explore the effects of different HF models, and how this translates to humans with HF.

Microbial dysbiosis in patients with CRC has been studied extensively, and patients with CRC present with a distinct microbial pattern. We observed several changes in bacteria that are often reported in patients with CRC, including a decrease in *Blautia*, *Alistipes*, *Lachnoclostridium*, and *Anaerotruncus* and an increase in *Desulfovibrio* and *Bacteroides*.^45–54^ Collectively, the changes observed in our murine HF model contain a credible overlap with the microbial changes observed in human patients with heart disease and patients with CRC.

The exact aetiology of gut dysbiosis in HF is unclear. It is hypothesized that HF induces gut congestion and low-grade inflammation, which is thought to affect gut permeability leading to dysregulation of the microbiome and increased bacterial translocation.^33^ However, in our murine studies we did not observe differences in expression of the tight junction proteins or *MUC2*, suggesting that gut permeability was not affected by the HF model. In addition, no changes in expression of inflammatory markers were observed in the colon, suggesting other mechanisms may be involved in HF-induced microbial dysbiosis.

The role of the microbiome in CRC tumour growth has been addressed before. FMT with faeces from CRC patients increased intestinal tumour growth in mice, compared to faeces from healthy individuals.^23,24^ In addition, several bacteria have been shown to possess tumourigenic properties.^55^ Interestingly, our data showed that HF-induced microbial dysbiosis can also contribute to tumour formation in a CRC mouse model, as FMT with faeces from MI mice resulted in higher tumour counts in a chemically induced CRC mouse model. Over 85% of the bacteria that were detected in the donor mice were also detected in the mice that received the faecal transplant, suggesting the FMT was successful. However, the mice that received the faecal transplant also showed multiple unique bacterial genera. This might be attributed to the processing of the faecal samples and the tumour phenotype, which could have affected the microbiota composition.

RNA sequencing analyses on the colon tissue of the CRC mice revealed Gsdmc3 and Ifit1 as the most significantly up-regulated genes in the CRC mice transplanted with faeces from MI mice. Interestingly, GSDMs are known to be activated by gut microbiota and GSDMc is associated with enhanced tumour growth in CRC mouse models.^56,57^ In addition, GSDMc is associated with poor prognosis in patients with breast or lung cancer.^58,59^ GSDMc induces a shift from apoptosis to pyroptosis,^60^ which was also observed in our RNA sequencing data as pyroptotic processes were up-regulated and apoptotic processes were down-regulated. However, how this process relates to tumourigenesis remains unclear.^60^ IFIT1 has been identified as a potential oncogene involved in F. Nucleatum-induced CRC tumour progression.^61^ In addition, IFIT1 up-regulation is associated with oral squamous cell carcinoma progression and non-small cell lung cancer.^62,63^ Our enrichment analyses revealed that defence response to symbiont, defence response, and cytokine-mediated signalling pathways were up-regulated processes in the tumours of mice transplanted with faeces from MI mice. Moreover, apoptosis-related processes were down-regulated and proliferation processes were up-regulated in the tumour tissues of mice transplanted with faeces from MI mice. This provides another layer of evidence that the MI-induced microbial dysbiosis may contribute to tumour formation.

At this stage, it remains unclear if our observations would represent an amenable target for therapy. In humans, ample experience has accumulated with FMT.^64,65^ Arguably, human patients with HF may benefit from FMT with faecal material from subjects free of HF. Whether this would lower the risk of incident cancer is speculative, and will be difficult to evaluate, since it would require a very sizeable trial. Supplementation with pro- or pre-biotics may be a more practical approach. Preclinical studies focussing on the effects of microbiota of healthy individuals on colonic tumour growth in a combined HF-CRC model could provide evidence whether the tumorigenic effects of HF could be rescued by microbiota transplantation.

In conclusion, our study provides a mechanistic demonstration of the role of HF in stimulating colonic tumour formation through the modulation of the microbiota composition. Recent preclinical studies have focused on the connection between HF and tumour growth, and several mechanisms have been identified.^6,7,9^ Our current findings highlight gut microbial alterations as a potential contributory factor promoting the development of intestinal tumours in the context of HF. This contributes to the expanding body of evidence, that HF increases the susceptibility to cancer in distant tissues. To advance our comprehension of this connection, fostering collaborations between cardiologists and oncologists in both clinical practice and research settings becomes imperative. Such partnerships are pivotal for delving deeper into the shared mechanisms between HF and cancer, paving the way for enhanced therapeutic strategies and a more comprehensive understanding of these interconnected conditions.

### 4.1 Limitations of the study

This study has several limitations. Some contradictory findings were observed between the HF mice and the mice transplanted with HF faeces, and between mouse and human data. Overall, variations in the microbial composition between studies are common. A meta-analysis on the microbial composition of CRC patients revealed that the results of different studies were far from coherent.^22^ The differences between studies may be attributed to the cancer phenotype, environmental factors (diet, medication), method of faeces collection, and sequencing methods. The microbial composition from mice does of course not fully resemble the microbial composition in humans; therefore translation to the human situation remains incomplete. We did however ascertain that the microbial changes in mice resemble those of humans. Further, the sequencing method we used allows analysis as deep as the genus level and is not able to provide detailed information on the specific bacterial species. Last, the microbial community is largely inhabited by anaerobic bacteria. Although we have optimized our protocols to limit exposure to oxygen before snap freezing, it may affect several anaerobic bacteria, and therefore may not be an entirely accurate resemblance to the original gut microbial composition.

## Supplementary Material

cvae038_Supplementary_Data

## Data Availability

This study did not generate new unique reagents. All commercially available materials used in this paper are listed in the Reagents and Tools Table. No original code was used in this paper. DOIs of the used codes are listed in the methods section. All 16S rRNA gene sequencing data used in this paper will be shared by the lead contact upon request.

## References

[1] de Wit S , Glen C, de Boer RA, Lang NN. Mechanisms shared between cancer, heart failure, and targeted anti-cancer therapies. Cardiovasc Res 2022;118:3451–3466. 10.1093/cvr/cvac132 PMC9897696 36004495

[2] de Boer RA , Hulot JS, Tocchetti CG, Aboumsallem JP, Ameri P, Anker SD, Bauersachs J, Bertero E, Coats AJS, Čelutkienė J, Chioncel O, Dodion P, Eschenhagen T, Farmakis D, Bayes-Genis A, Jäger D, Jankowska EA, Kitsis RN, Konety SH, Larkin J, Lehmann L, Lenihan DJ, Maack C, Moslehi JJ, Müller OJ, Nowak-Sliwinska P, Piepoli MF, Ponikowski P, Pudil R, Rainer PP, Ruschitzka F, Sawyer D, Seferovic PM, Suter T, Thum T, van der Meer P, Van Laake LW, von Haehling S, Heymans S, Lyon AR, Backs J. Common mechanistic pathways in cancer and heart failure. A scientific roadmap on behalf of the Translational Research Committee of the Heart Failure Association (HFA) of the European Society of Cardiology (ESC). Eur J Heart Fail 2020;22:2272–2289. 33094495 10.1002/ejhf.2029 PMC7894564

[3] Hasin T , Gerber Y, McNallan SM, Weston SA, Kushwaha SS, Nelson TJ, Cerhan JR, Roger VL. Patients with heart failure have an increased risk of incident cancer. J Am Coll Cardiol 2013;62:881–886. 23810869 10.1016/j.jacc.2013.04.088 PMC3758775

[4] Banke A , Schou M, Videbæk L, Møller JE, Torp-Pedersen C, Gustafsson F, Dahl JS, Køber L, Hildebrandt PR, Gislason GH. Incidence of cancer in patients with chronic heart failure: a long-term follow-up study. Eur J Heart Fail 2016;18:260–266. 26751260 10.1002/ejhf.472

[5] Bertero E , Robusto F, Rulli E, D’Ettorre A, Bisceglia L, Staszewsky L, Maack C, Lepore V, Latini R, Ameri P. Cancer incidence and mortality according to pre-existing heart failure in a community-based cohort. JACC CardioOncol 2022;4:98–109. 35492831 10.1016/j.jaccao.2021.11.007 PMC9040106

[6] Meijers WC , Maglione M, Bakker SJL, Oberhuber R, Kieneker LM, de Jong S, Haubner BJ, Nagengast WB, Lyon AR, van der Vegt B, van Veldhuisen DJ, Westenbrink BD, van der Meer P, Silljé HHW, de Boer RA. Heart failure stimulates tumor growth by circulating factors. Circulation 2018;138:678–691. 29459363 10.1161/CIRCULATIONAHA.117.030816

[7] Avraham S , Abu-Sharki S, Shofti R, Haas T, Korin B, Kalfon R, Friedman T, Shiran A, Saliba W, Shaked Y, Aronheim A. Early cardiac remodeling promotes tumor growth and metastasis. Circulation 2020;142:670–683. 32475164 10.1161/CIRCULATIONAHA.120.046471

[8] Koelwyn GJ , Newman AAC, Afonso MS, van Solingen C, Corr EM, Brown EJ, Albers KB, Yamaguchi N, Narke D, Schlegel M, Sharma M, Shanley LC, Barrett TJ, Rahman K, Mezzano V, Fisher EA, Park DS, Newman JD, Quail DF, Nelson ER, Caan BJ, Jones LW, Moore KJ. Myocardial infarction accelerates breast cancer via innate immune reprogramming. Nat Med 2020:26:1452–1458. 32661390 10.1038/s41591-020-0964-7 PMC7789095

[9] Awwad L , Aronheim A. Cardiac dysfunction promotes cancer progression via multiple secreted factors. Cancer Res 2022;82:1753–1761. 35260887 10.1158/0008-5472.CAN-21-2463 PMC9359722

[10] Awwad L , Goldenberg T, Langier-Goncalves I, Aronheim A. Cardiac remodeling in the absence of cardiac contractile dysfunction is sufficient to promote cancer progression. Cells 2022;11:1108. 35406672 10.3390/cells11071108 PMC8997578

[11] Luedde M , Winkler T, Heinsen FA, Rühlemann MC, Spehlmann ME, Bajrovic A, Lieb W, Franke A, Ott SJ, Frey N. Heart failure is associated with depletion of core intestinal microbiota. ESC Hear Fail 2017;4:282–290. 10.1002/ehf2.12155 PMC5542738 28772054

[12] Beale AL , O’Donnell JA, Nakai ME, Nanayakkara S, Vizi D, Carter K, Dean E, Ribeiro RV, Yiallourou S, Carrington MJ, Marques FZ, Kaye DM. The gut microbiome of heart failure with preserved ejection fraction. J Am Heart Assoc 2021;10:20654. 10.1161/JAHA.120.020654 PMC8403331 34212778

[13] Tang WH , Wang Z, Levison BS, Koeth RA, Britt EB, Fu X, Wu Y, Hazen SL. Intestinal microbial metabolism of phosphatidylcholine and cardiovascular risk. N Engl J Med 2013;368:1575–1584. 23614584 10.1056/NEJMoa1109400 PMC3701945

[14] Li Z , Wu Z, Yan J, Liu H, Liu Q, Deng Y, Ou C, Chen M. Gut microbe-derived metabolite trimethylamine N-oxide induces cardiac hypertrophy and fibrosis. Lab Invest 2019;99:346–357. 30068915 10.1038/s41374-018-0091-y

[15] Wu P , Chen JN, Chen JJ, Tao J, Wu SY, Xu GS, Wang Z, Wei DH, Yin WD. Trimethylamine N-oxide promotes apoE-/- mice atherosclerosis by inducing vascular endothelial cell pyroptosis via the SDHB/ROS pathway. J Cell Physiol 2020;235:6582–6591. 32012263 10.1002/jcp.29518

[16] Wu ZX , Li SF, Chen H, Song JX, Gao YF, Zhang F, Cao CF. The changes of gut microbiota after acute myocardial infarction in rats. PLoS One 2017;12:e0180717. 28686722 10.1371/journal.pone.0180717 PMC5501596

[17] Zheng A , Yi H, Li F, Han L, Yu J, Cheng X, Su H, Hong K, Li J. Changes in gut microbiome structure and function of rats with isoproterenol-induced heart failure. Int Heart J 2019;60:1176–1183. 31564708 10.1536/ihj.18-194

[18] Carrillo-Salinas FJ , Anastasiou M, Ngwenyama N, Kaur K, Tai A, Smolgovsky SA, Jetton D, Aronovitz M, Alcaide P. Gut dysbiosis induced by cardiac pressure overload enhances adverse cardiac remodeling in a T cell-dependent manner. Gut Microbes 2020;12:1–20. 10.1080/19490976.2020.1823801 PMC7588211 33103561

[19] Gagnière J , Raisch J, Veziant J, Barnich N, Bonnet R, Buc E, Bringer MA, Pezet D, Bonnet M. Gut microbiota imbalance and colorectal cancer. World J Gastroenterol 2020;22:501–518. 10.3748/wjg.v22.i2.501 PMC4716055 26811603

[20] Fan X , Jin Y, Chen G, Ma X, Zhang L. Gut microbiota dysbiosis drives the development of colorectal cancer. Digestion 2021;102:508–515. 32932258 10.1159/000508328

[21] Koliarakis I , Messaritakis I, Nikolouzakis TK, Hamilos G, Souglakos J, Tsiaoussis J. Oral bacteria and intestinal dysbiosis in colorectal cancer. Int J Mol Sci 2019;20:4146. 31450675 10.3390/ijms20174146 PMC6747549

[22] Mo Z , Huang P, Yang C, Xiao S, Zhang G, Ling F, Li L. Meta-analysis of 16S rRNA microbial data identified distinctive and predictive microbiota dysbiosis in colorectal carcinoma adjacent tissue. mSystems 2020;5:e00138–e00120. 32291348 10.1128/mSystems.00138-20 PMC7159898

[23] Wong SH , Zhao L, Zhang X, Nakatsu G, Han J, Xu W, Xiao X, Kwong TNY, Tsoi H, Wu WKK, Zeng B, Chan FKL, Sung JJY, Wei H, Yu J. Gavage of fecal samples from patients with colorectal cancer promotes intestinal carcinogenesis in germ-free and conventional mice. Gastroenterology 2017;153:1621–1633.e6. 28823860 10.1053/j.gastro.2017.08.022

[24] Li L , Li X, Zhong W, Yang M, Xu M, Sun Y, Ma J, Liu T, Song X, Dong W, Liu X, Chen Y, Liu Y, Abla Z, Liu W, Wang B, Jiang K, Cao H. Gut microbiota from colorectal cancer patients enhances the progression of intestinal adenoma in Apcmin/+ mice. EBioMedicine 2019;48:301–315. 31594750 10.1016/j.ebiom.2019.09.021 PMC6838415

[25] Melleby AO , Romaine A, Aronsen JM, Veras I, Zhang L, Sjaastad I, Lunde IG, Christensen G. A novel method for high precision aortic constriction that allows for generation of specific cardiac phenotypes in mice. Cardiovasc Res 2018;114:1680–1690. 29878127 10.1093/cvr/cvy141

[26] Becker C , Fantini MC, Wirtz S, Nikolaev A, Kiesslich R, Lehr HA, Galle PR, Neurath MF. In vivo imaging of colitis and colon cancer development in mice using high resolution chromoendoscopy. Gut 2005;54:950–954. 15951540 10.1136/gut.2004.061283 PMC1774595

[27] maaslin2—The Huttenhower Lab. https://huttenhower.sph.harvard.edu/maaslin/. (17 December 2023).

[28] Ge SX , Jung D, Yao R. ShinyGO: a graphical gene-set enrichment tool for animals and plants. Bioinformatics 2020;36:2628–2629. 31882993 10.1093/bioinformatics/btz931 PMC7178415

[29] Brandsma E , Kloosterhuis NJ, Koster M, Dekker DC, Gijbels MJJ, van der Velden S, Ríos-Morales M, van Faassen MJR, Loreti MG, de Bruin A, Fu J, Kuipers F, Bakker BM, Westerterp M, de Winther MPJ, Hofker MH, van de Sluis B, Koonen DPY. A proinflammatory gut Microbiota increases systemic inflammation and accelerates atherosclerosis. Circ Res 2019;124:94–100. 30582442 10.1161/CIRCRESAHA.118.313234 PMC6325767

[30] Aboumsallem JP , Shi C, De Wit S, Markousis-Mavrogenis G, Bracun V, Eijgenraam TR, Hoes MF, Meijers WC, Screever EM, Schouten ME, Voors AA, Silljé HHW, De Boer R. Multi-omics analyses identify molecular signatures with prognostic values in different heart failure aetiologies. J Mol Cell Cardiol 2023;175:13–28. 36493852 10.1016/j.yjmcc.2022.12.001

[31] Takahashi K , Nishida A, Fujimoto T, Fujii M, Shioya M, Imaeda H, Inatomi O, Bamba S, Sugimoto M, Andoh A. Reduced abundance of butyrate-producing bacteria species in the fecal microbial community in Crohn’s disease. Digestion 2016;93:59–65. 26789999 10.1159/000441768

[32] Vacca M , Celano G, Calabrese FM, Portincasa P, Gobbetti M, De Angelis M. The controversial role of human gut Lachnospiraceae. Microorganisms 2020;8:573. 32326636 10.3390/microorganisms8040573 PMC7232163

[33] Tang WHW , Kitai T, Hazen SL. Gut microbiota in cardiovascular health and disease. Circ Res 2017;120:1183–1196. 28360349 10.1161/CIRCRESAHA.117.309715 PMC5390330

[34] Karbach SH , Schönfelder T, Brandão I, Wilms E, Hörmann N, Jäckel S, Schüler R, Finger S, Knorr M, Lagrange J, Brandt M, Waisman A, Kossmann S, Schäfer K, Münzel T, Reinhardt C, Wenzel P. Gut microbiota promote angiotensin II–induced arterial hypertension and vascular dysfunction. J Am Heart Assoc 2016;5:e003698. 27577581 10.1161/JAHA.116.003698 PMC5079031

[35] Edwards JM , Roy S, Tomcho JC, Schreckenberger ZJ, Chakraborty S, Bearss NR, Saha P, McCarthy CG, Vijay-Kumar M, Joe B, Wenceslau CF. Microbiota are critical for vascular physiology: germ-free status weakens contractility and induces sex-specific vascular remodeling in mice. Vascul Pharmacol 2020;125–126:106633. 10.1016/j.vph.2019.106633 PMC7036036 31843471

[36] Karlsson FH , Fåk F, Nookaew I, Tremaroli V, Fagerberg B, Petranovic D, Bäckhed F, Nielsen J. Symptomatic atherosclerosis is associated with an altered gut metagenome. Nat Commun 2012;3:1245. 23212374 10.1038/ncomms2266 PMC3538954

[37] Jie Z , Xia H, Zhong SL, Feng Q, Li S, Liang S, Zhong H, Liu Z, Gao Y, Zhao H, Zhang D, Su Z, Fang Z, Lan Z, Li J, Xiao L, Li J, Li R, Li X, Li F, Ren H, Huang Y, Peng Y, Li G, Wen B, Dong B, Chen JY, Geng QS, Zhang ZW, Yang H, Wang J, Wang J, Zhang X, Madsen L, Brix S, Ning G, Xu X, Liu X, Hou Y, Jia H, He K, Kristiansen K. The gut microbiome in atherosclerotic cardiovascular disease. Nat Commun 2017;8:845. 29018189 10.1038/s41467-017-00900-1 PMC5635030

[38] Kamo T , Akazawa H, Suda W, Saga-Kamo A, Shimizu Y, Yagi H, Liu Q, Nomura S, Naito AT, Takeda N, Harada M, Toko H, Kumagai H, Ikeda Y, Takimoto E, Suzuki JI, Honda K, Morita H, Hattori M, Komuro I. Dysbiosis and compositional alterations with aging in the gut microbiota of patients with heart failure. PLoS One 2017;12:e0174099. 28328981 10.1371/journal.pone.0174099 PMC5362204

[39] Katsimichas T , Ohtani T, Motooka D, Tsukamoto Y, Kioka H, Nakamoto K, Konishi S, Chimura M, Sengoku K, Miyawaki H, Sakaguchi T, Okumura R, Theofilis K, Iida T, Takeda K, Nakamura S, Sakata Y. Non-ischemic heart failure with reduced ejection fraction is associated with altered intestinal microbiota. Circ J 2018;82:1640–1650. 29607983 10.1253/circj.CJ-17-1285

[40] Zhu Q , Gao R, Zhang Y, Pan D, Zhu Y, Zhang X, Yang R, Jiang R, Xu Y, Qin H. Dysbiosis signatures of gut microbiota in coronary artery disease. Physiol Genomics 2018;50:893–903. 30192713 10.1152/physiolgenomics.00070.2018

[41] Kummen M , Mayerhofer CCK, Vestad B, Broch K, Awoyemi A, Storm-Larsen C, Ueland T, Yndestad A, Hov JR, Trøseid M. Gut microbiota signature in heart failure defined from profiling of 2 independent cohorts. J Am Coll Cardiol 2018;71:1184–1186. 29519360 10.1016/j.jacc.2017.12.057

[42] Cui X , Ye L, Li J, Jin L, Wang W, Li S, Bao M, Wu S, Li L, Geng B, Zhou X, Zhang J, Cai J. Metagenomic and metabolomic analyses unveil dysbiosis of gut microbiota in chronic heart failure patients. Sci Rep 2018;8:635. 29330424 10.1038/s41598-017-18756-2 PMC5766622

[43] Boccella N , Paolillo R, Coretti L, D’Apice S, Lama A, Giugliano G, Schiattarella GG, Cuomo M, d’Aquino I, Cavaliere G, Paciello O, Mollica MP, Mattace Raso G, Esposito G, Lembo F, Perrino C. Transverse aortic constriction induces gut barrier alterations, microbiota remodeling and systemic inflammation. Sci Rep 2021;11:7404. 33795775 10.1038/s41598-021-86651-y PMC8016915

[44] Spehlmann ME , Rangrez AY, Dhotre DP, Schmiedel N, Chavan N, Bang C, Müller OJ, Shouche YS, Franke A, Frank D, Frey N. Heart failure severity closely correlates with intestinal dysbiosis and subsequent metabolomic alterations. Biomedicines 2022;10:809. 35453559 10.3390/biomedicines10040809 PMC9033061

[45] Chen W , Liu F, Ling Z, Tong X, Xiang C. Human intestinal lumen and mucosa-associated microbiota in patients with colorectal cancer. PLoS One 2012;7:e39743. 22761885 10.1371/journal.pone.0039743 PMC3386193

[46] Wang T , Cai G, Qiu Y, Fei N, Zhang M, Pang X, Jia W, Cai S, Zhao L. Structural segregation of gut microbiota between colorectal cancer patients and healthy volunteers. ISME J 2012;6:320–329. 21850056 10.1038/ismej.2011.109 PMC3260502

[47] Wu N , Yang X, Zhang R, Li J, Xiao X, Hu Y, Chen Y, Yang F, Lu N, Wang Z, Luan C, Liu Y, Wang B, Xiang C, Wang Y, Zhao F, Gao GF, Wang S, Li L, Zhang H, Zhu B. Dysbiosis signature of fecal Microbiota in colorectal cancer patients. Microb Ecol 2013;66:462–470. 23733170 10.1007/s00248-013-0245-9

[48] Mori G , Rampelli S, Orena BS, Rengucci C, De Maio G, Barbieri G, Passardi A, Casadei Gardini A, Frassineti GL, Gaiarsa S, Albertini AM, Ranzani GN, Calistri D, Pasca MR. Shifts of faecal Microbiota during sporadic colorectal carcinogenesis. Sci Reports 2018;8:10329. 10.1038/s41598-018-28671-9 PMC6037773 29985435

[49] Yachida S , Mizutani S, Shiroma H, Shiba S, Nakajima T, Sakamoto T, Watanabe H, Masuda K, Nishimoto Y, Kubo M, Hosoda F, Rokutan H, Matsumoto M, Takamaru H, Yamada M, Matsuda T, Iwasaki M, Yamaji T, Yachida T, Soga T, Kurokawa K, Toyoda A, Ogura Y, Hayashi T, Hatakeyama M, Nakagama H, Saito Y, Fukuda S, Shibata T, Yamada T. Metagenomic and metabolomic analyses reveal distinct stage-specific phenotypes of the gut microbiota in colorectal cancer. Nat Med 2019;25:968–976. 31171880 10.1038/s41591-019-0458-7

[50] Yang Y , Du L, Shi D, Kong C, Liu J, Liu G, Li X, Ma Y. Dysbiosis of human gut microbiome in young-onset colorectal cancer. Nat Commun 2021;12:6757. 34799562 10.1038/s41467-021-27112-y PMC8604900

[51] Park J , Kim NE, Yoon H, Shin CM, Kim N, Lee DH, Park JY, Choi CH, Kim JG, Kim YK, Shin TS, Yang J, Park YS. Fecal microbiota and gut microbe-derived extracellular vesicles in colorectal cancer. Front Oncol 2021;11:650026. 34595105 10.3389/fonc.2021.650026 PMC8477046

[52] Du X , Li Q, Tang Z, Yan L, Zhang L, Zheng Q, Zeng X, Chen G, Yue H, Li J, Zhao M, Han YP, Fu X. Alterations of the gut microbiome and fecal metabolome in colorectal cancer: implication of intestinal metabolism for tumorigenesis. Front Physiol 2022;13:854545. 35600308 10.3389/fphys.2022.854545 PMC9116530

[53] Coker OO , Liu C, Wu WKK, Wong SH, Jia W, Sung JJY, Yu J. Altered gut metabolites and microbiota interactions are implicated in colorectal carcinogenesis and can be non-invasive diagnostic biomarkers. Microbiome 2022;10:35. 35189961 10.1186/s40168-021-01208-5 PMC8862353

[54] Zhang H , Chang Y, Zheng Q, Zhang R, Hu C, Jia W. Altered intestinal microbiota associated with colorectal cancer. Front Med 2019;13:461–470. 31250341 10.1007/s11684-019-0695-7

[55] Cullin N , Azevedo Antunes C, Straussman R, Stein-Thoeringer CK, Elinav E. Microbiome and cancer. Cancer Cell 2021;39:1317–1341. 34506740 10.1016/j.ccell.2021.08.006

[56] Privitera G , Rana N, Scaldaferri F, Armuzzi A, Pizarro TT. Novel insights into the interactions between the gut microbiome, inflammasomes, and gasdermins during colorectal cancer. Front Cell Infect Microbiol 2022;11:806680. 35111698 10.3389/fcimb.2021.806680 PMC8801609

[57] Miguchi M , Hinoi T, Shimomura M, Adachi T, Saito Y, Niitsu H, Kochi M, Sada H, Sotomaru Y, Ikenoue T, Shigeyasu K, Tanakaya K, Kitadai Y, Sentani K, Oue N, Yasui W, Ohdan H. Gasdermin C is upregulated by inactivation of transforming growth factor β receptor type II in the presence of mutated Apc, promoting colorectal cancer proliferation. PLoS One 2016;11:e0166422. 27835699 10.1371/journal.pone.0166422 PMC5105946

[58] Hou J , Zhao R, Xia W, Chang CW, You Y, Hsu JM, Nie L, Chen Y, Wang YC, Liu C, Wang WJ, Wu Y, Ke B, Hsu JL, Huang K, Ye Z, Yang Y, Xia X, Li Y, Li CW, Shao B, Tainer JA, Hung MC. PD-L1-mediated gasdermin C expression switches apoptosis to pyroptosis in cancer cells and facilitates tumour necrosis. Nat Cell Biol 2020;22:1264–1275. 32929201 10.1038/s41556-020-0575-z PMC7653546

[59] Wei J , Xu Z, Chen X, Wang X, Zeng S, Qian L, Yang X, Ou C, Lin W, Gong Z, Yan Y. Overexpression of GSDMC is a prognostic factor for predicting a poor outcome in lung adenocarcinoma. Mol Med Rep 2020;21:360–370. 31939622 10.3892/mmr.2019.10837 PMC6896373

[60] Hou J , Hsu J-M, Hung M-C. Molecular mechanisms and functions of pyroptosis in inflammation and antitumor immunity. Mol Cell 2021;81:4579–4590. 34562371 10.1016/j.molcel.2021.09.003 PMC8604761

[61] Gao Y , Zou T, Xu P, Wang Y, Jiang Y, Chen YX, Chen H, Hong J, Fang JY. Fusobacterium nucleatum stimulates cell proliferation and promotes PD-L1 expression via IFIT1-related signal in colorectal cancer. Neoplasia 2023;35:100850. 36371909 10.1016/j.neo.2022.100850 PMC9664554

[62] Pidugu VK , Wu MM, Yen AH, Pidugu HB, Chang KW, Liu CJ, Lee TC. IFIT1 and IFIT3 promote oral squamous cell carcinoma metastasis and contribute to the anti-tumor effect of gefitinib via enhancing p-EGFR recycling. Oncogene 2019;38:3232–3247. 30626937 10.1038/s41388-018-0662-9

[63] Zan X , Li S, Wei S, Gao L, Zhao L, Yan X, Zhao Y, Shi J, Wang Y, Liu R, Zhang Y, Wan Y, Zhou Y. COL8A1 promotes NSCLC progression through IFIT1/IFIT3-mediated EGFR activation. Front Oncol 2022;12:707525. 35280763 10.3389/fonc.2022.707525 PMC8907630

[64] Liu X , Li Y, Wu K, Shi Y, Chen M. Fecal microbiota transplantation as therapy for treatment of active ulcerative colitis: a systematic review and meta-analysis. Gastroenterol Res Pract 2021;2021:6612970. 33981340 10.1155/2021/6612970 PMC8088370

[65] Antushevich H . Fecal microbiota transplantation in disease therapy. Clin Chim Acta 2020;503:90–98. 31968211 10.1016/j.cca.2019.12.010

