# Supplementary material for: Heart failure-induced microbial dysbiosis contributes to colonic tumour formation in mice
Source: Cardiovasc Res. 2024 Feb 24;120(6):612–22. doi: 10.1093/cvr/cvae038 (PMC11074794; doi:10.1093/cvr/cvae038)
Supplement: cvae038_Supplementary_Data [file cvae038_supplementary_data.zip › Supplemental Figure legends - revised (2).docx]

# Supplemental figure legends

**Supplemental Figure 1. Body weight, food intake and colonic function of HF mice. A.** Body weight changes **B.** Weekly food intake. **C.** SCFA levels in the caecum. **D.** Gene expression levels, normalized for 36B4 expression. **E.** Representative images of Claudin-3 immunofluorescence staining on colon tissue (counterstained with DAPI and WGA staining), scalebar: 100µm. **F.** Gene expression levels of Claudin-3, normalized for 36B4 expression. **G.** Representative images of ZO-1 immunofluorescence staining on colon tissue (counterstained with DAPI and WGA staining), scalebar: 100µm. **H.** Gene expression levels of ZO-1, normalized for 36B4 expression. Data are represented as mean ±SD. Statistical significance was determined using 2-way ANOVA (A-B) or Mann-Whitney U-test C, D, F, H). P<0.05 is considered as statistically significant. Sham n=11, MI n=8, TAC n=9 (TNFα: sham n=11, MI n=7; IL6: sham n=5, MI n=7).

**Supplemental Figure 2**. **Effects of TAC-induced HF on the gut microbial composition. A.** Left ventricular ejection fraction (LVEF) **B.** Heart weight normalized for tibia length **C.** Left ventricular inner diameter systole (LVIDs) **D.** Relative *natriuretic peptide A (Nppa)* expression in LV, normalized for *peptidyl-prolyl cis-trans isomerase (Ppia)*. α-Diversity analysis represented by **E.** the number of observed species and **F.** Shannon diversity index. **G.** β-diversity index represented by principle coordinates analysis (PCoA). **H.** Volcano plot showing differentially abundant bacterial genera, represented by -log10(q-value)(bacterial genera are coloured according to the phylum they belong to). Data from A-D have previously been published by Aboumsallem *et al.* ^69^ Data are represented as mean ±SD. Statistical significance was determined using Mann-Whitney U-test (A-F) or MaAslin2, FDR q<0.25% (H). P<0.05 is considered as statistically significant. Sham n=11, TAC n=9.

**Supplemental Figure 3**. **Microbial composition of the pooled donor samples.** **A.** Left ventricular ejection fraction (LVEF) **B.** Heart weight normalized for tibia length **C.** Left ventricular inner diameter systole (LVIDs) **D.** Relative *natriuretic peptide A (Nppa)* expression in LV, normalized for *peptidyl-prolyl cis-trans isomerase (Ppia)*. Changes in **E.** body weight, and **F.** food and **G.** water intake of the donor mice. α-Diversity analysis represented by **H.** the number of observed species and **I.** Shannon diversity index. **J.** β-diversity index represented by principle coordinates analysis (PCoA). **K.** Volcano plot showing differentially abundant bacterial genera, represented by -log10(q-value)(bacterial genera are coloured according to the phylum they belong to) of the pooled donor faecal samples. Data are represented as mean ±SD. Statistical significance was determined using Mann-Whitney U-test (A-D, H-I), 2-way ANOVA (E-G) or MaAslin2 (K), FDR q<0.25%. P<0.05 is considered as statistically significant. Sham n=8, MI n=9 (A-G), 4 technical replicates per group (H-K).

**Supplemental Figure 4. Effect of DSS on body weight, colon length and stool consistency.** Changes in **A.** Body weight and **B.** Disease severity score of the transplanted mice, during DSS treatment. **C.** Colon length of the transplanted mice. Data are represented as mean ±SD. Statistical significance was determined using 2-way ANOVA (A-B) or Students t-test (C). P<0.05 is considered as statistically significant. N=14 per group.
