# Supplementary material for: Heart failure-induced microbial dysbiosis contributes to colonic tumour formation in mice
Source: Cardiovasc Res. 2024 Feb 24;120(6):612–22. doi: 10.1093/cvr/cvae038 (PMC11074794; doi:10.1093/cvr/cvae038)
Supplement: cvae038_Supplementary_Data [file cvae038_supplementary_data.zip › Supplemental File 2.pdf]

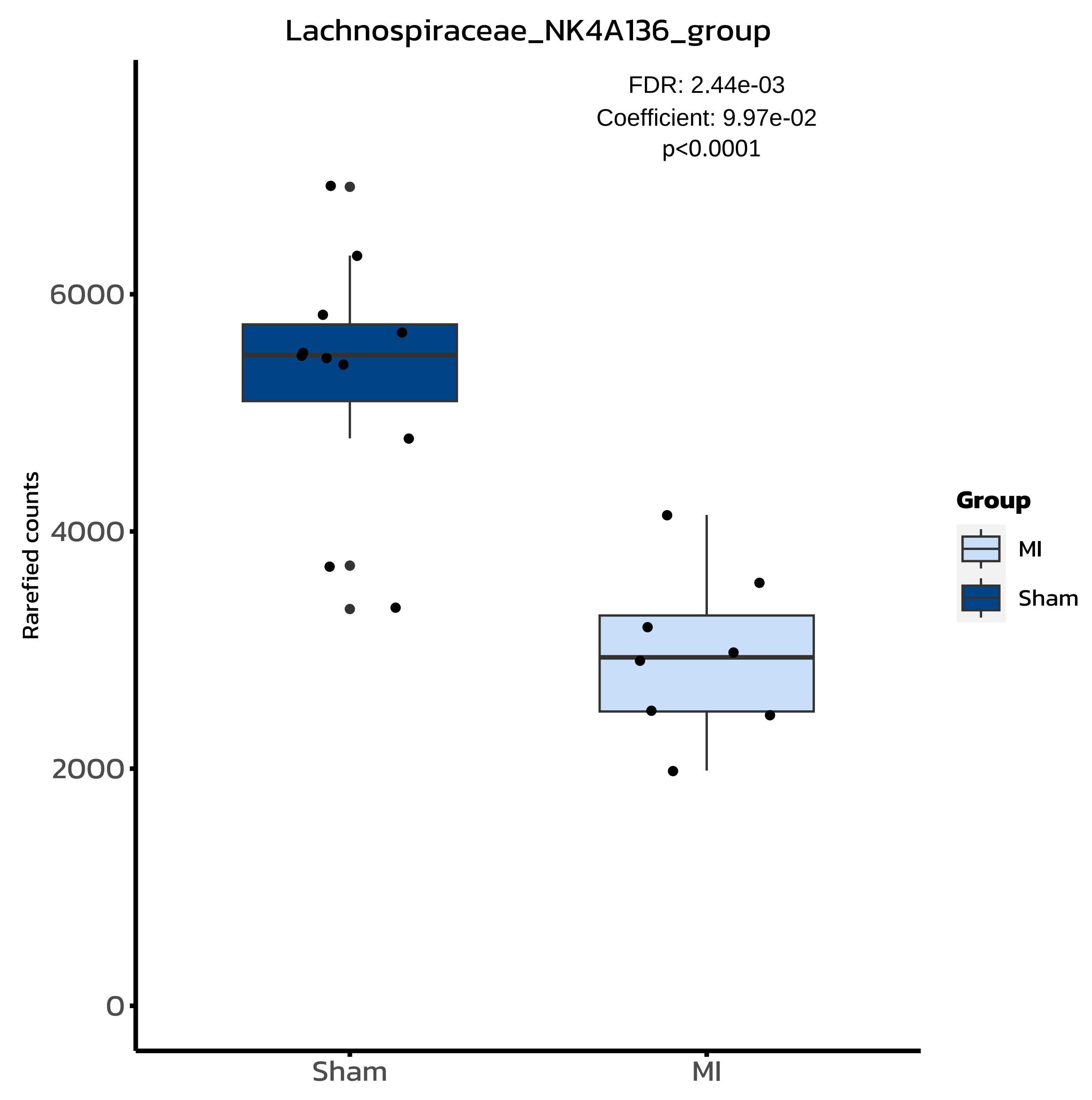
Lachnospiraceae_NK4A136_group
FDR: 2.44e-03
Coefficient: 9.97e-02
p<0.0001
Rarefied counts
6000
4000
2000
0
Sham
MI
Group
MI
Sham

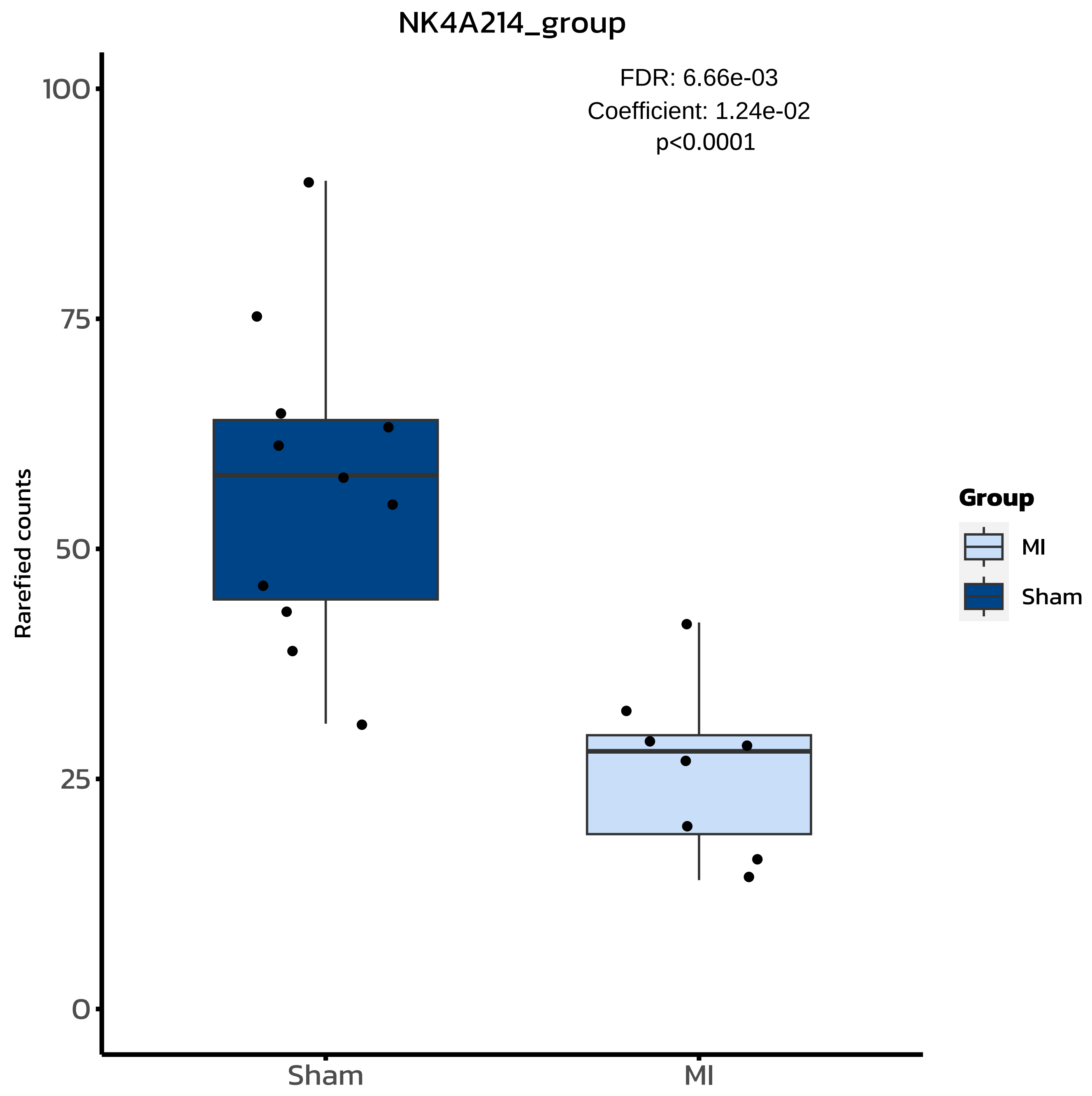
NK4A214_group
FDR: 6.66e-03
Coefficient: 1.24e-02
p<0.0001
Rarefied counts
100
75
50
25
0
Sham
MI
Group
MI
Sham

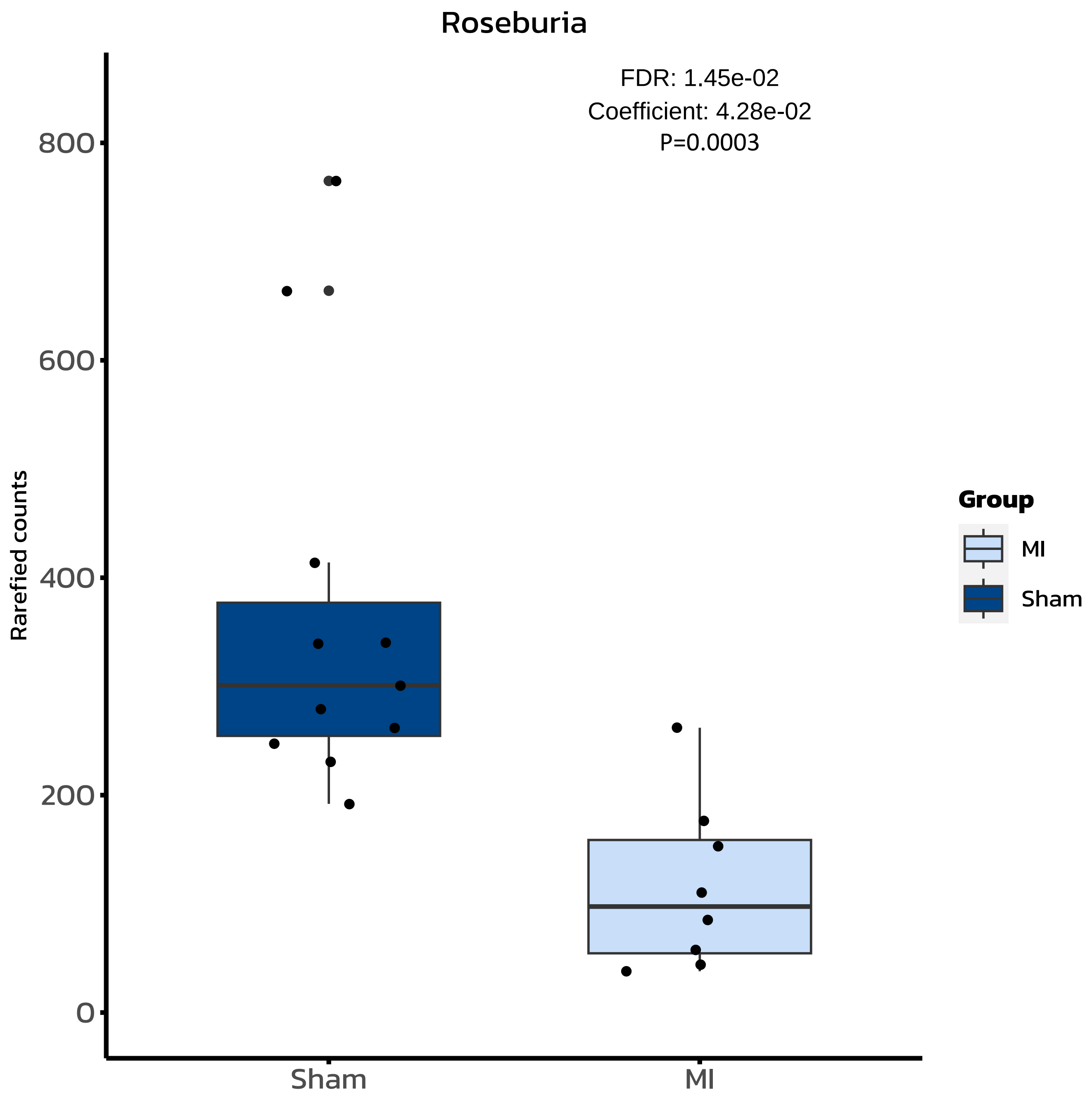

Roseburia
FDR: 1.45e-02
Coefficient: 4.28e-02
P=0.0003
Rarefied counts
800
600
400
200
0
Sham
MI
Group
MI
Sham

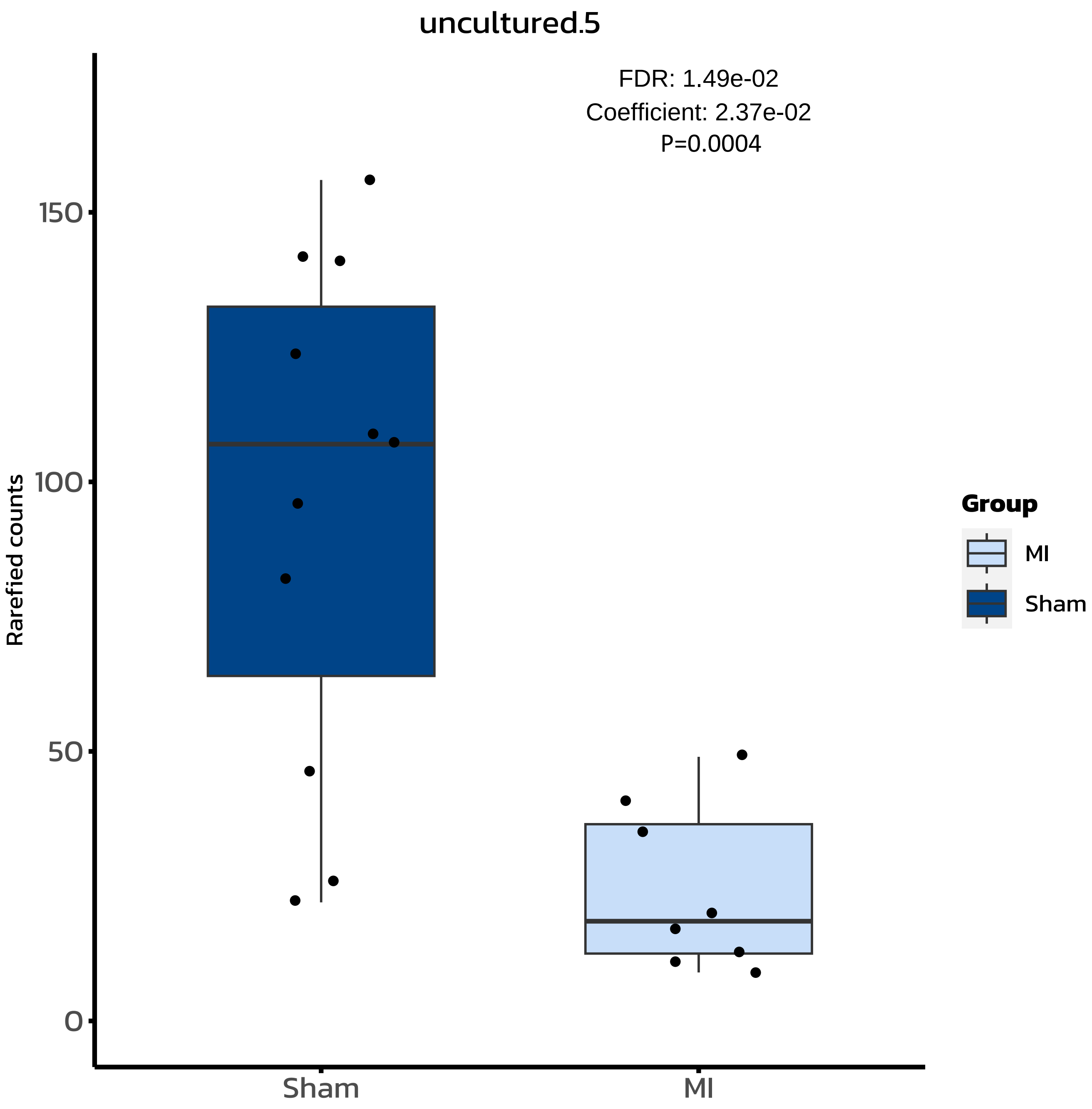
uncultured.5
FDR: 1.49e-02
Coefficient: 2.37e-02
P=0.0004
Rarefied counts
150
100
50
0
Sham
MI
Group
MI
Sham

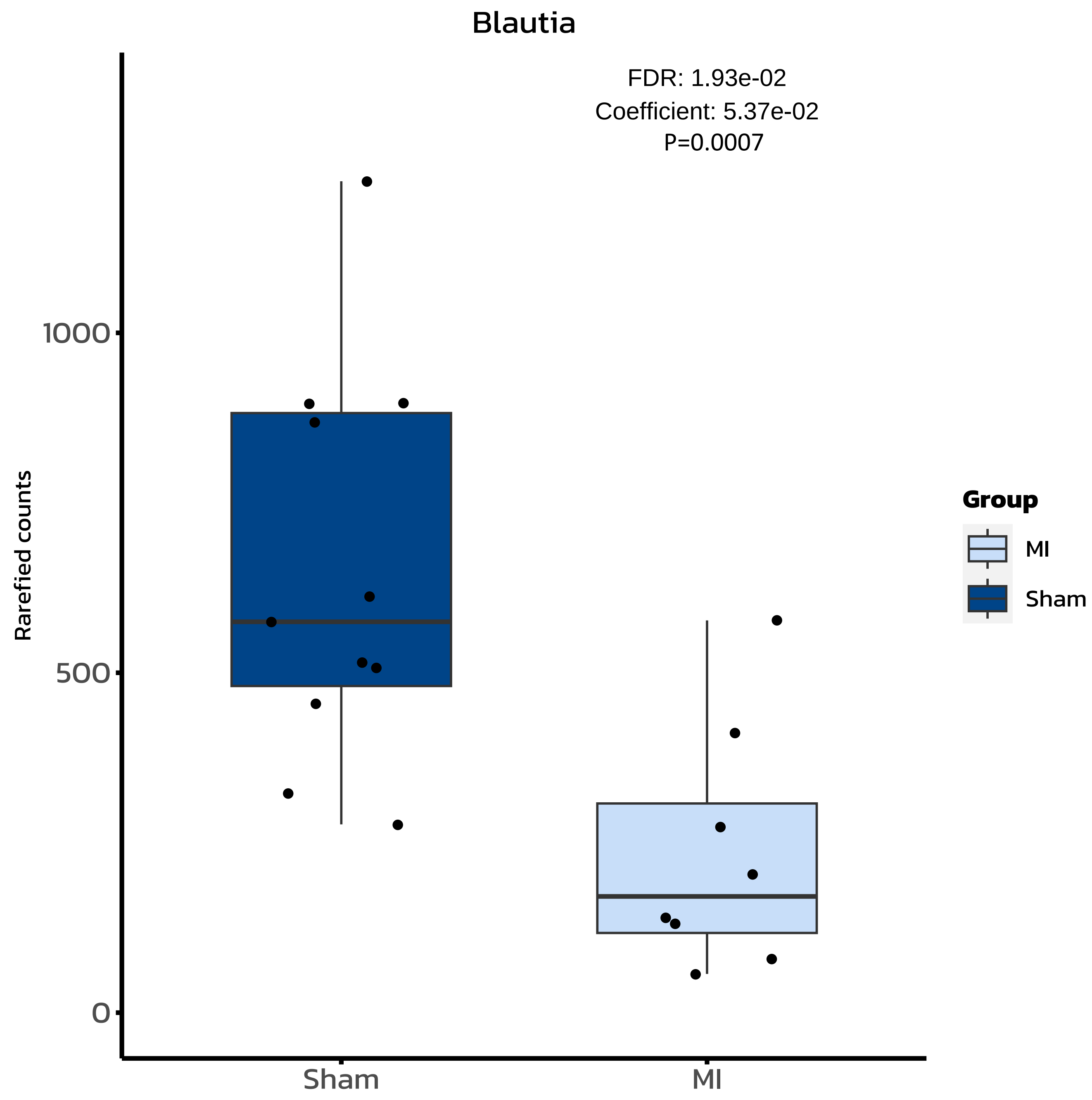
Blautia
FDR: 1.93e-02
Coefficient: 5.37e-02
P=0.0007
Rarefied counts
1000
500
0
Sham
MI
Group
MI
Sham

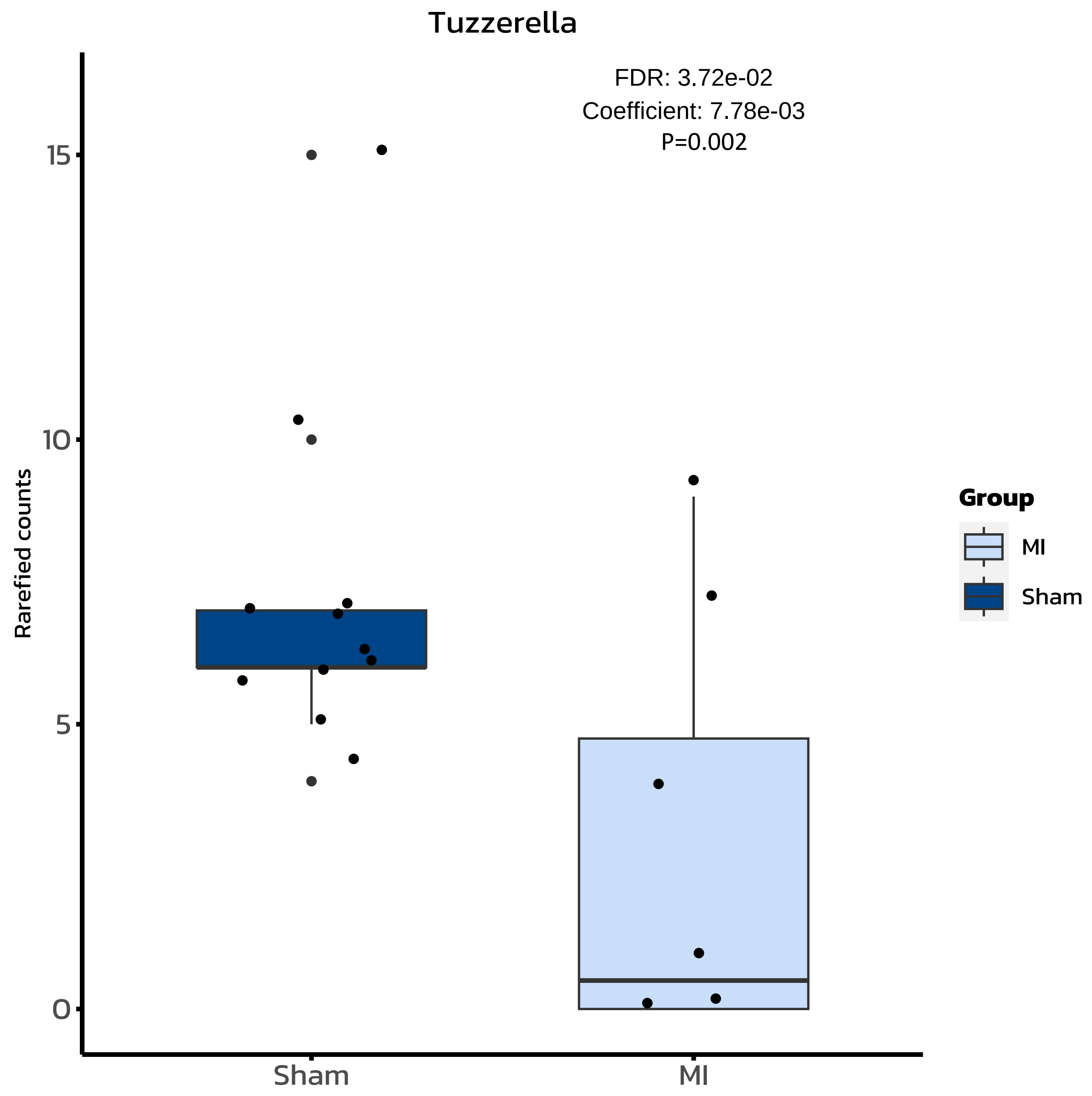
Tuzzerella
FDR: 3.72e-02
Coefficient: 7.78e-03
P=0.002
Rarefied counts
15
10
5
0
Sham
MI
Group
MI
Sham

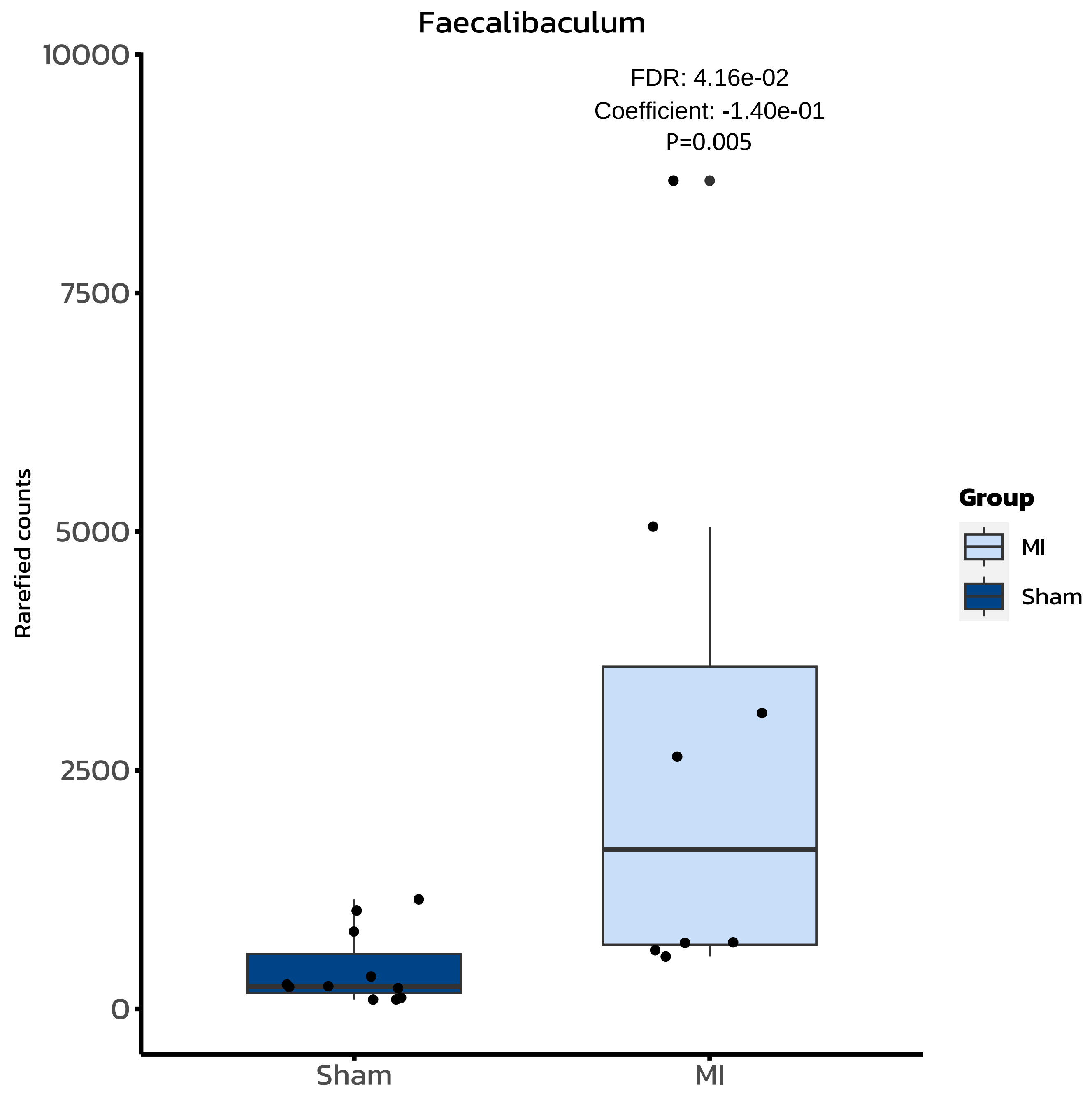
Faecalibaculum
FDR: 4.16e-02
Coefficient: -1.40e-01
P=0.005
10000
7500
5000
2500
0
Rarefied counts
Sham
MI
Group
MI
Sham

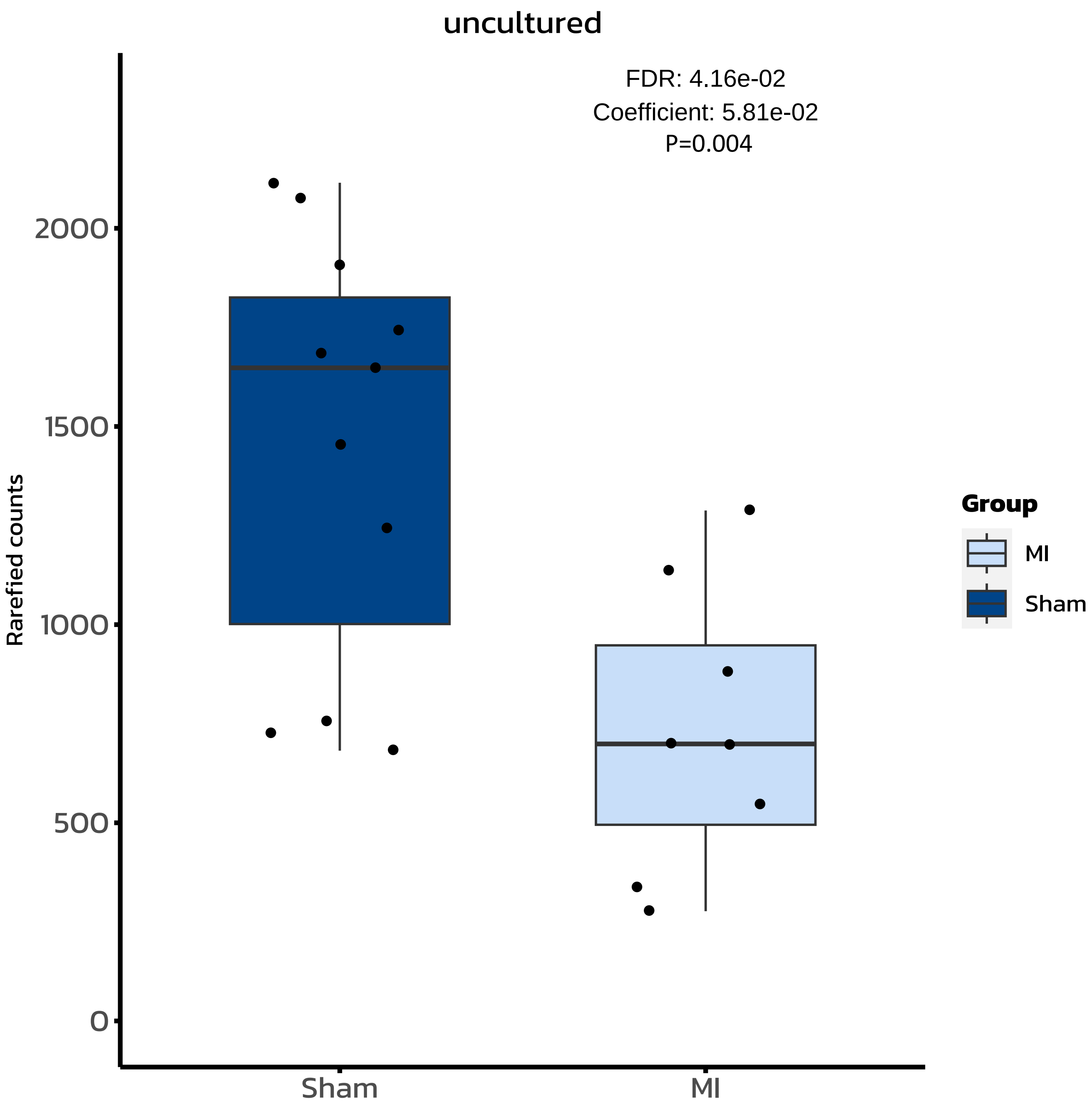

uncultured
FDR: 4.16e-02
Coefficient: 5.81e-02
P=0.004
Rarefied counts
2000
1500
1000
500
0
Sham
MI
Group
MI
Sham

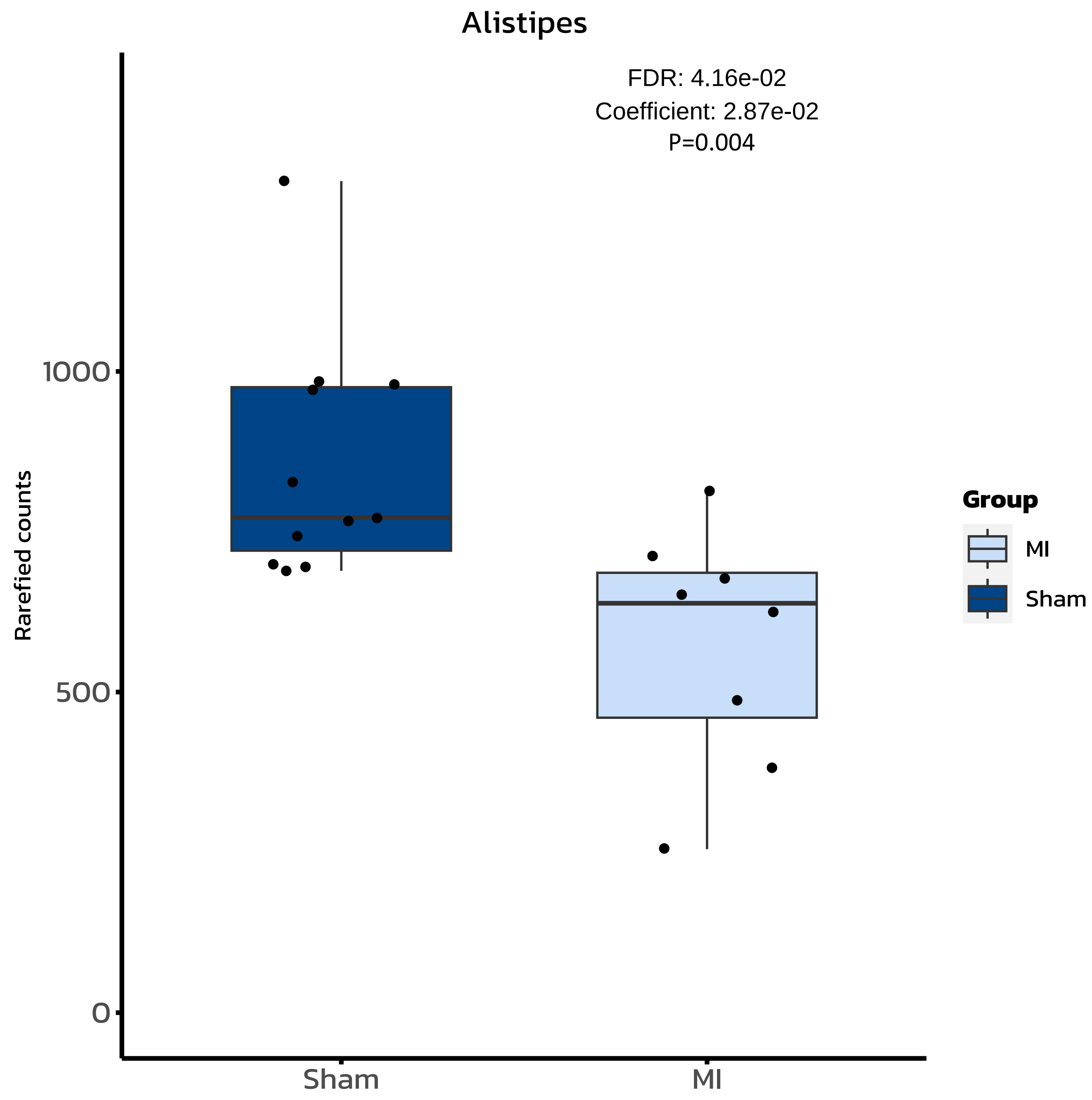
Alistipes
FDR: 4.16e-02
Coefficient: 2.87e-02
P=0.004
Rarefied counts
1000
500
0
Sham
MI
Group
MI
Sham

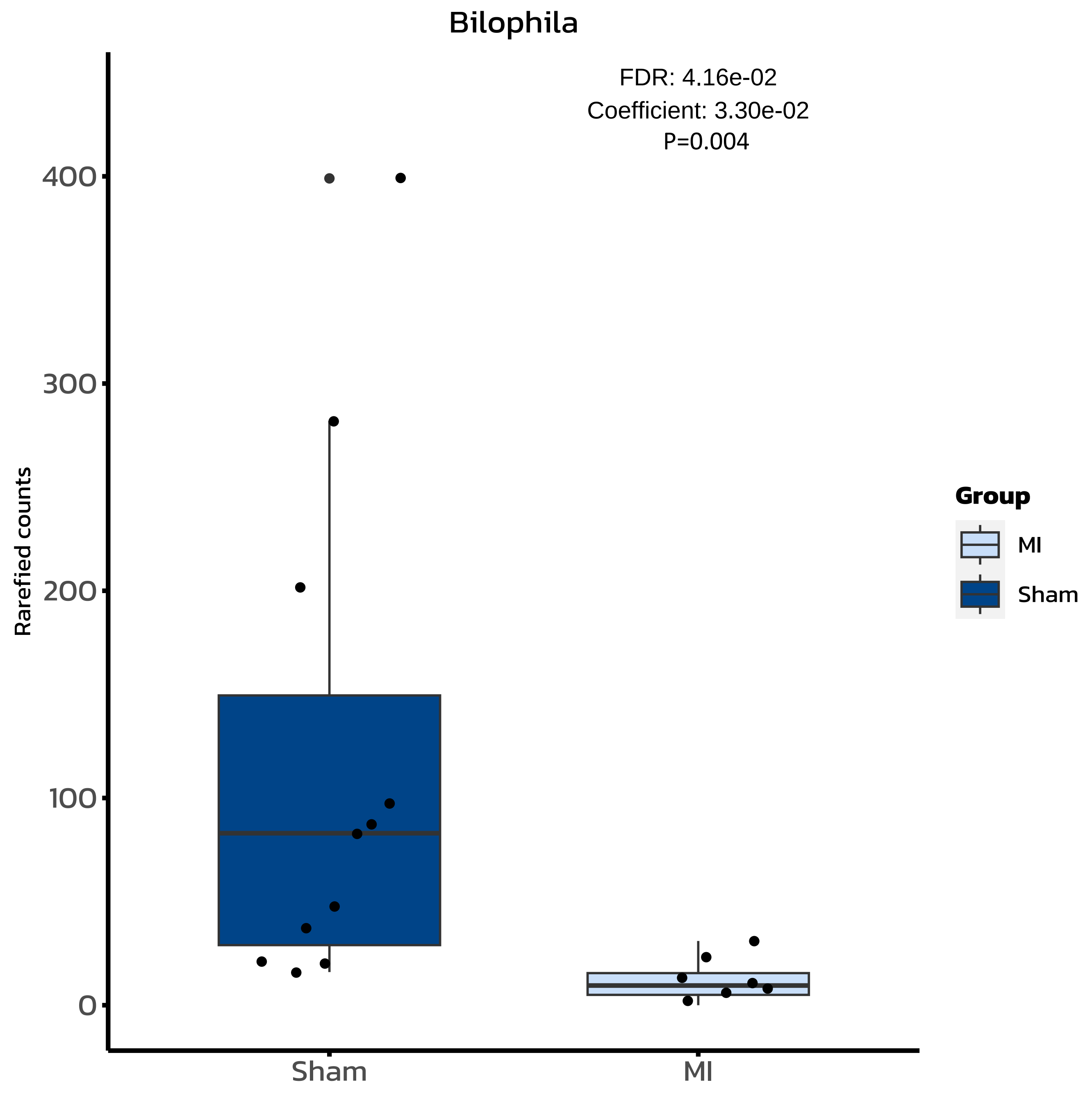

Bilophila
FDR: 4.16e-02
Coefficient: 3.30e-02
P=0.004
Rarefied counts
400
300
200
100
0
Sham
MI
Group
MI
Sham

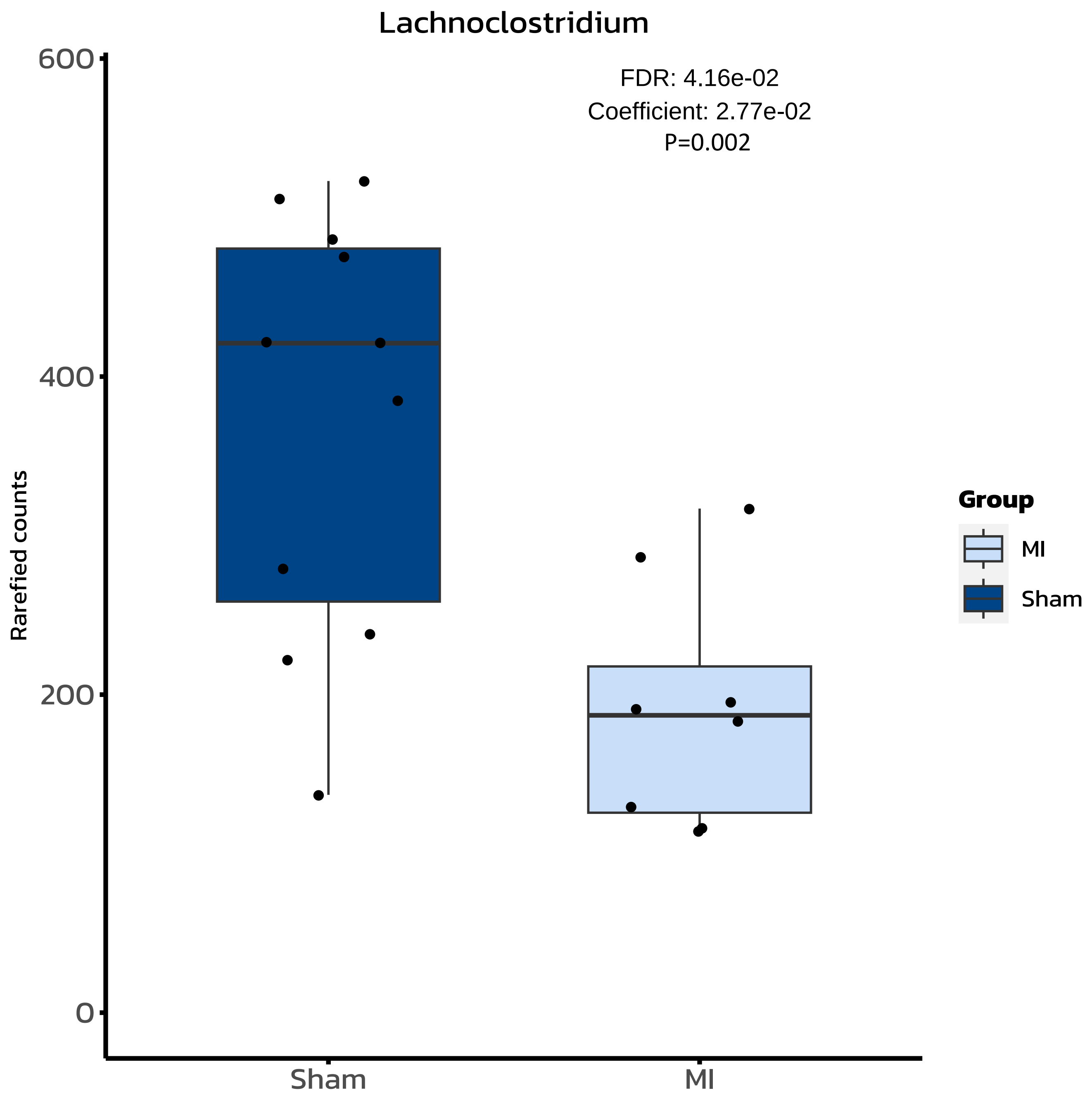
Lachnoclostridium
FDR: 4.16e-02
Coefficient: 2.77e-02
P=0.002
Rarefied counts
600
400
200
0
Sham
MI
Group
MI
Sham

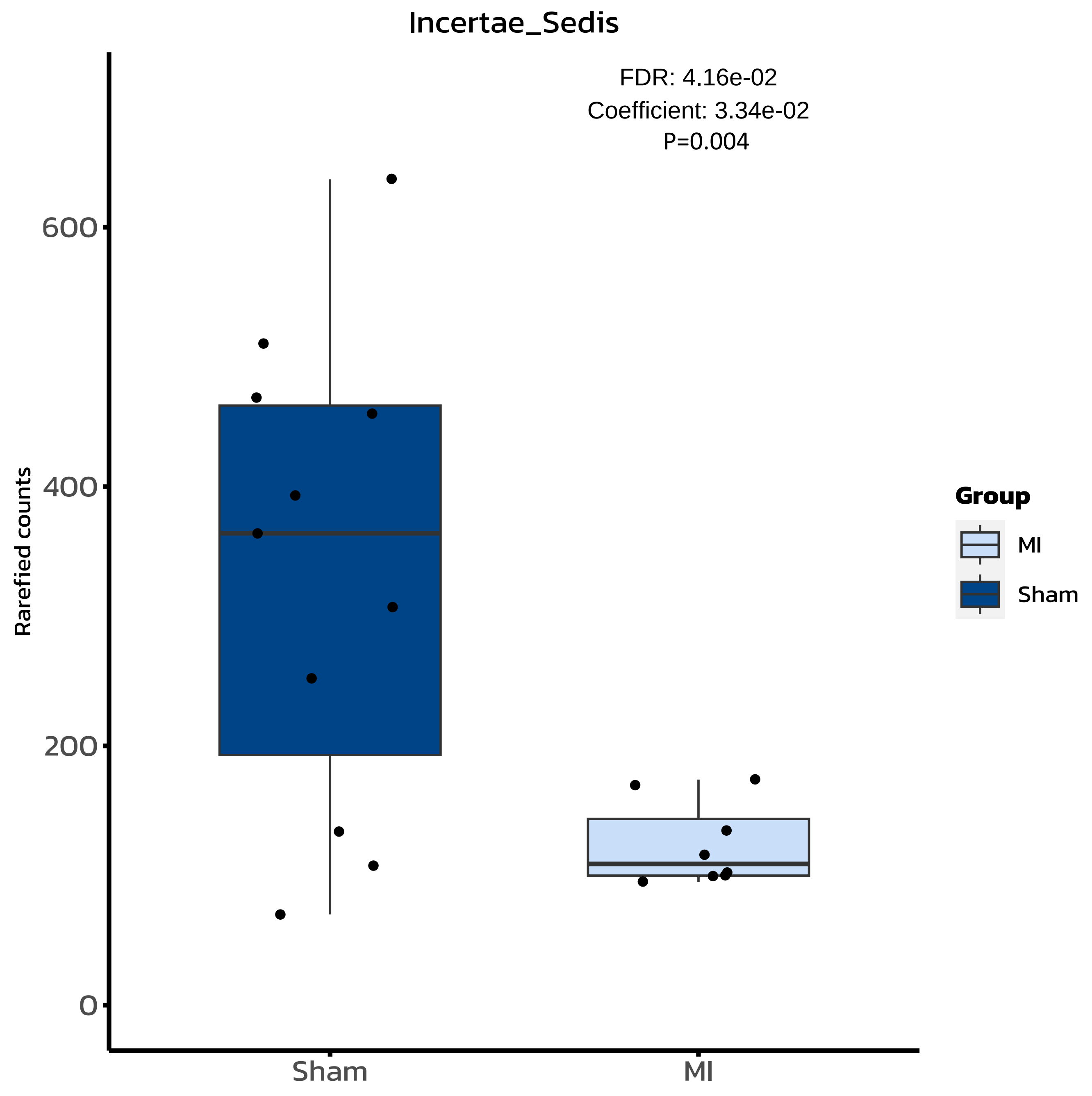
Incertae_Sedis
FDR: 4.16e-02
Coefficient: 3.34e-02
P=0.004
Rarefied counts
600
400
200
0
Sham
MI
Group
MI
Sham

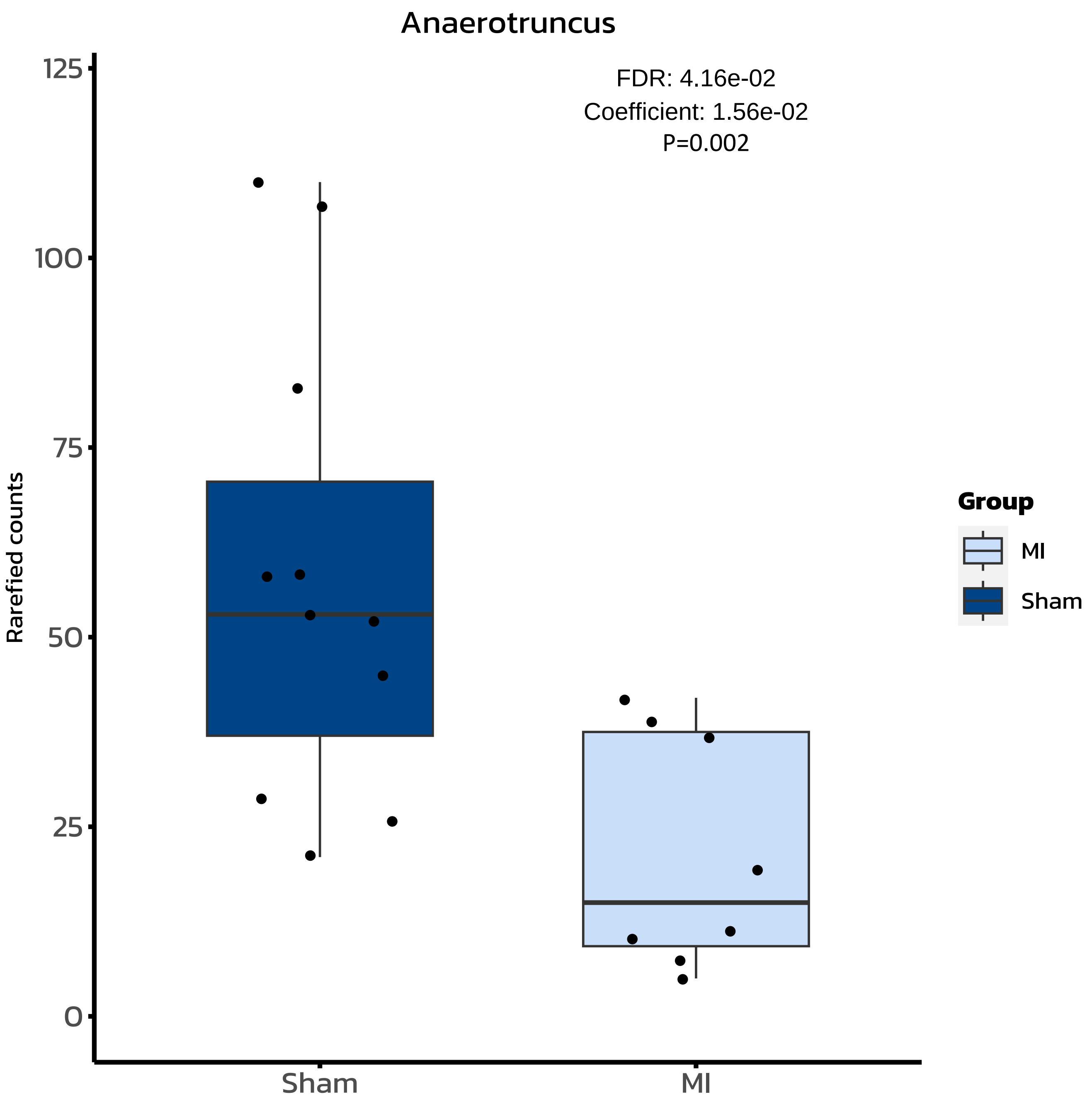

Anaerotruncus
FDR: 4.16e-02
Coefficient: 1.56e-02
P=0.002
Rarefied counts
125
100
75
50
25
0
Sham
MI
Group
MI
Sham

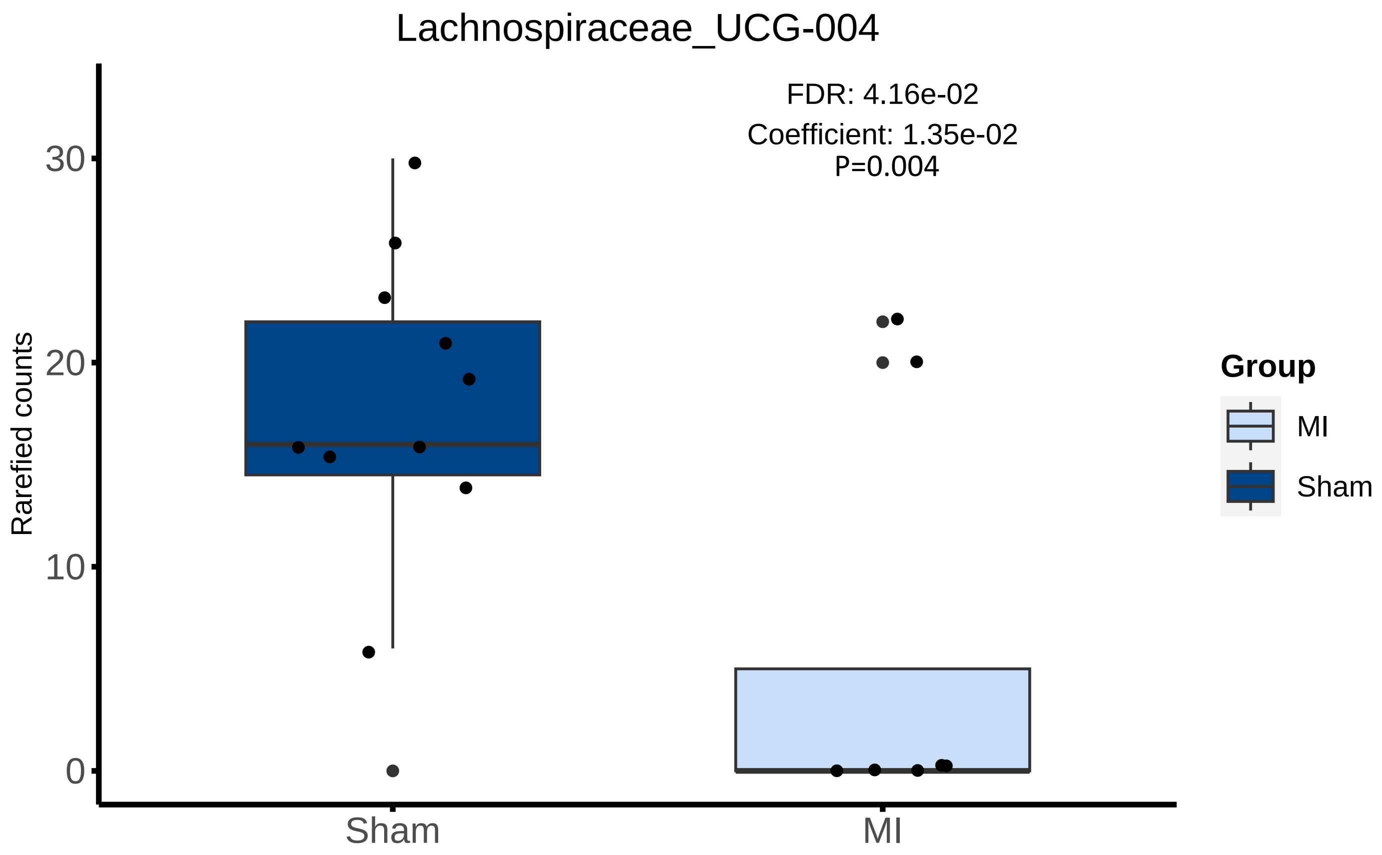

Lachnospiraceae_UCG-004
FDR: 4.16e-02
Coefficient: 1.35e-02
P=0.004
Rarefied counts
30
20
10
0
Sham
MI
Group
MI
Sham

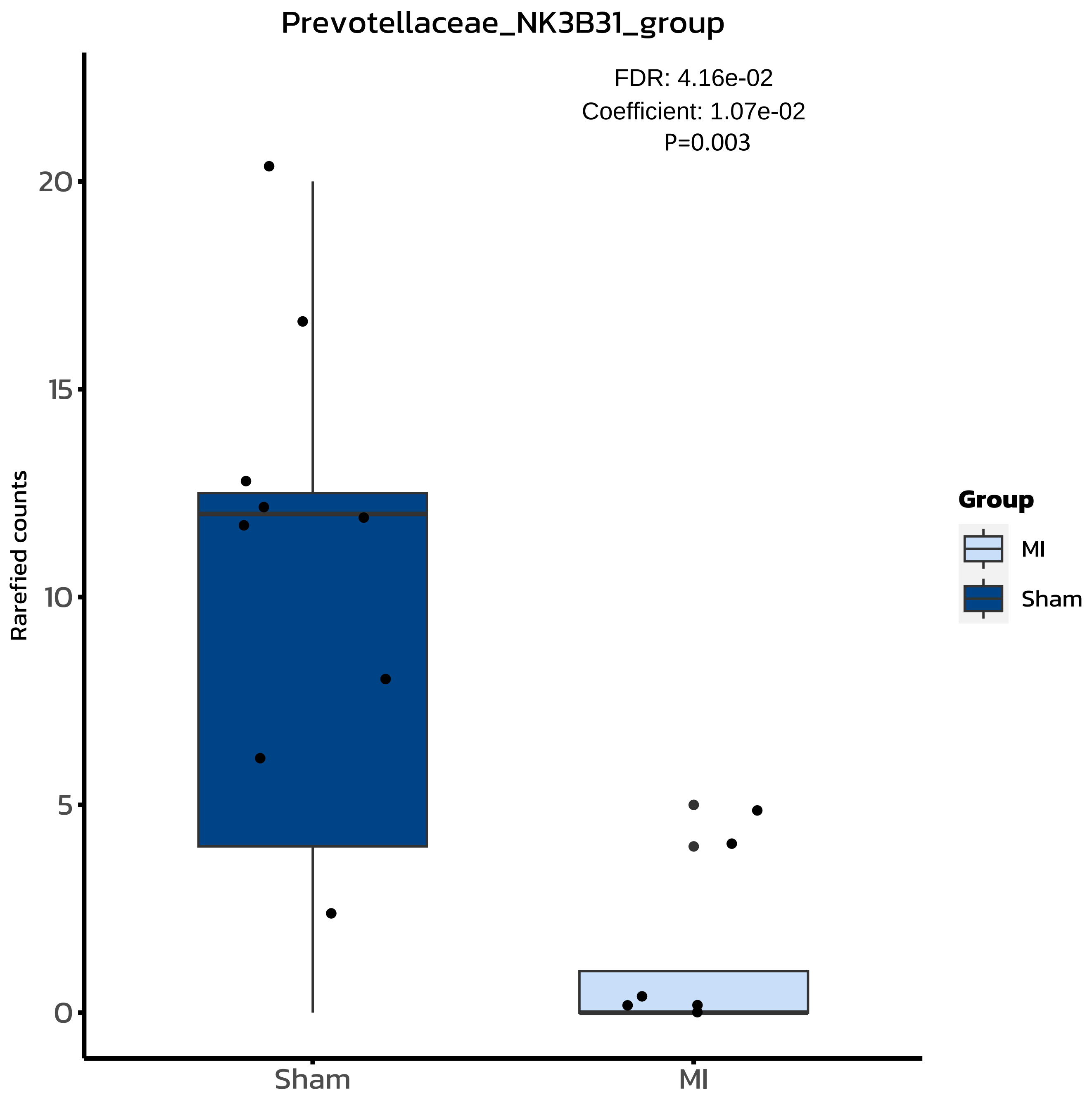

Prevotellaceae_NK3B31_group
FDR: 4.16e-02
Coefficient: 1.07e-02
P=0.003
Rarefied counts
20
15
10
5
0
Sham
MI
Group
MI
Sham

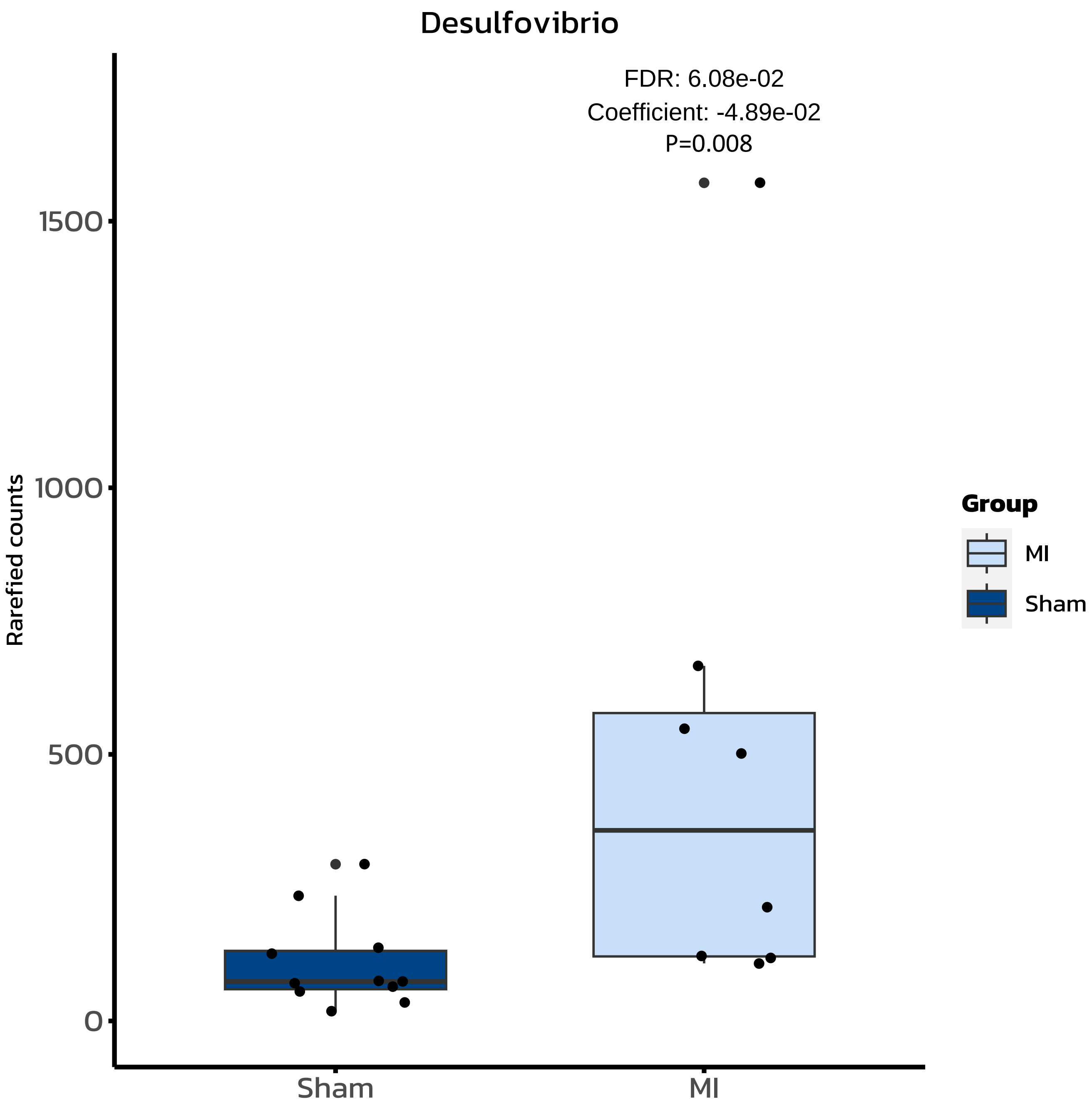
Desulfovibrio
FDR: 6.08e-02
Coefficient: -4.89e-02
P=0.008
Rarefied counts
1500
1000
500
0
Sham
MI
Group
MI
Sham

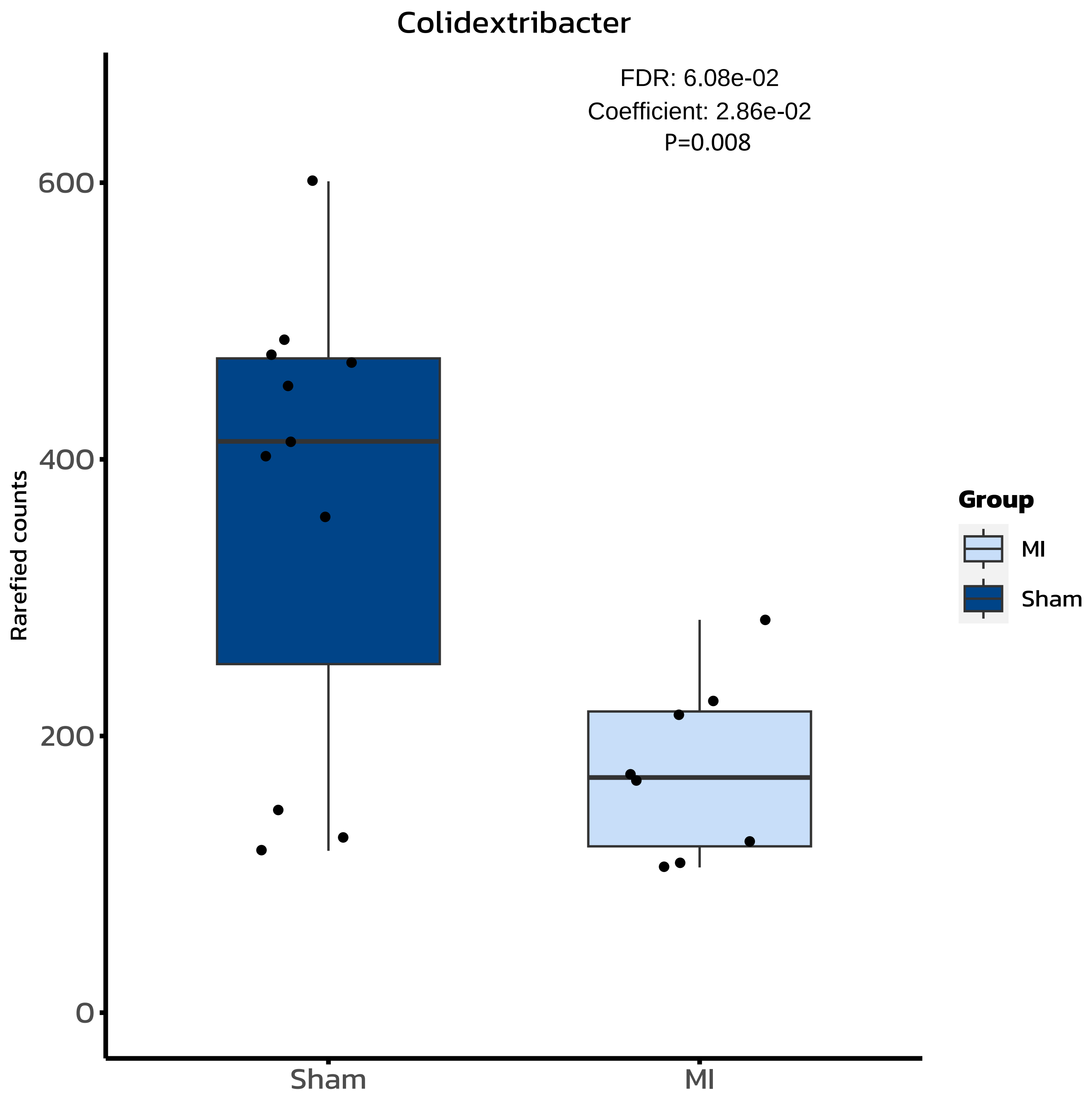
Colidextribacter
FDR: 6.08e-02
Coefficient: 2.86e-02
P=0.008
Rarefied counts
600
400
200
0
Sham
MI
Group
MI
Sham

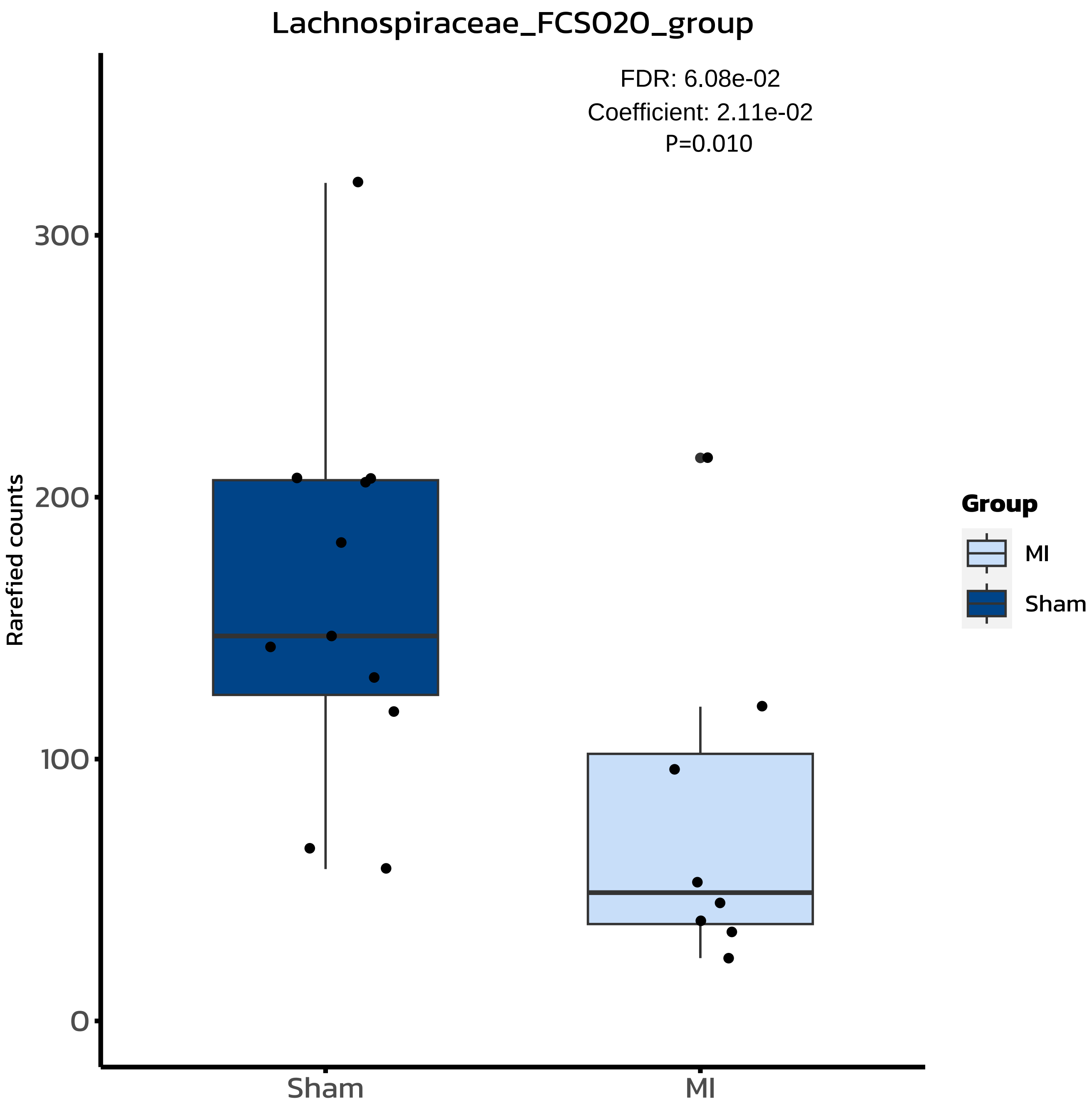

Lachnospiraceae_FCS020_group
FDR: 6.08e-02
Coefficient: 2.11e-02
P=0.010
Rarefied counts
300
200
100
0
Sham
MI
Group
MI
Sham

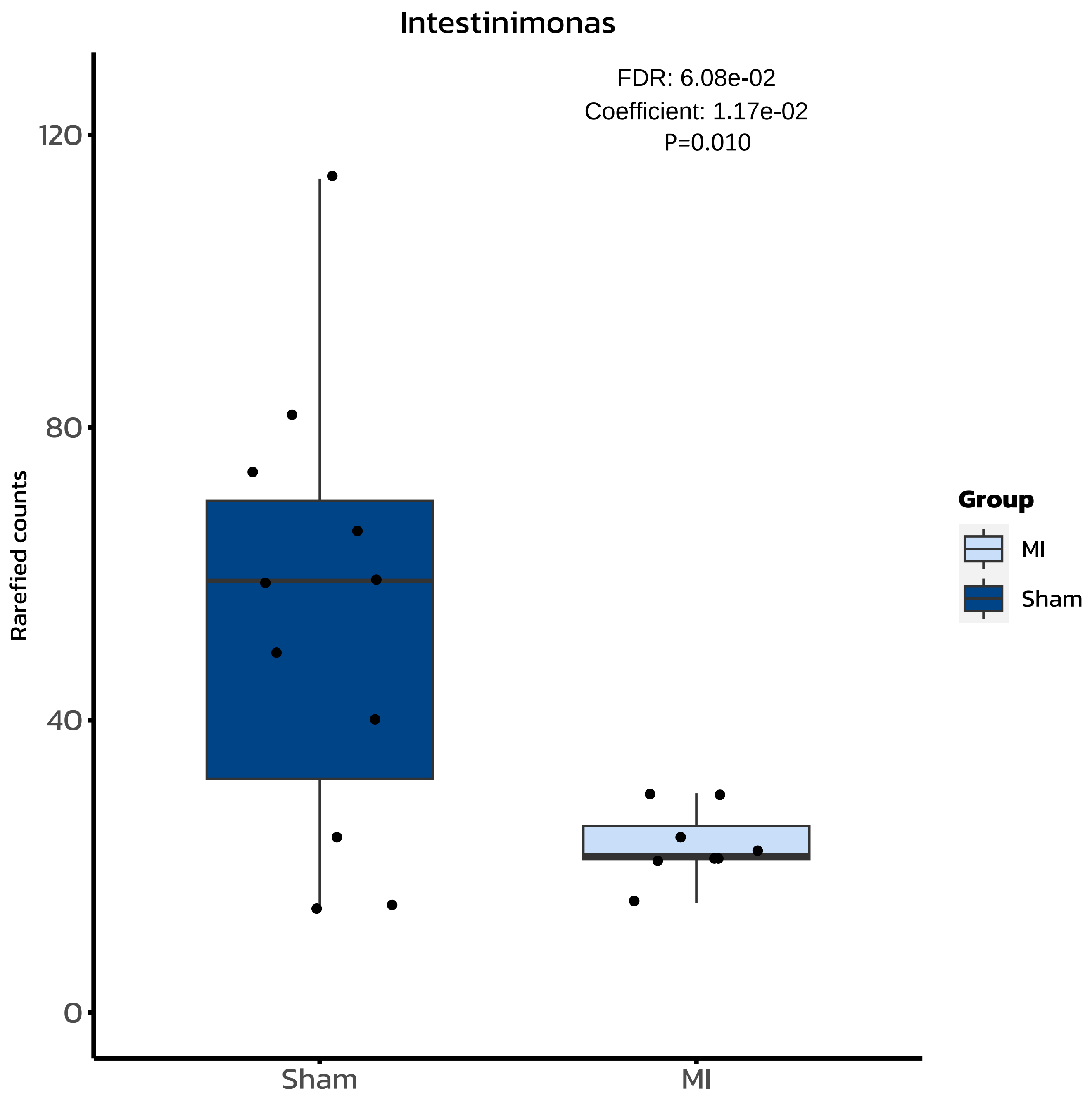
Intestinimonas
FDR: 6.08e-02
Coefficient: 1.17e-02
P=0.010
Rarefied counts
120
80
40
0
Sham
MI
Group
MI
Sham

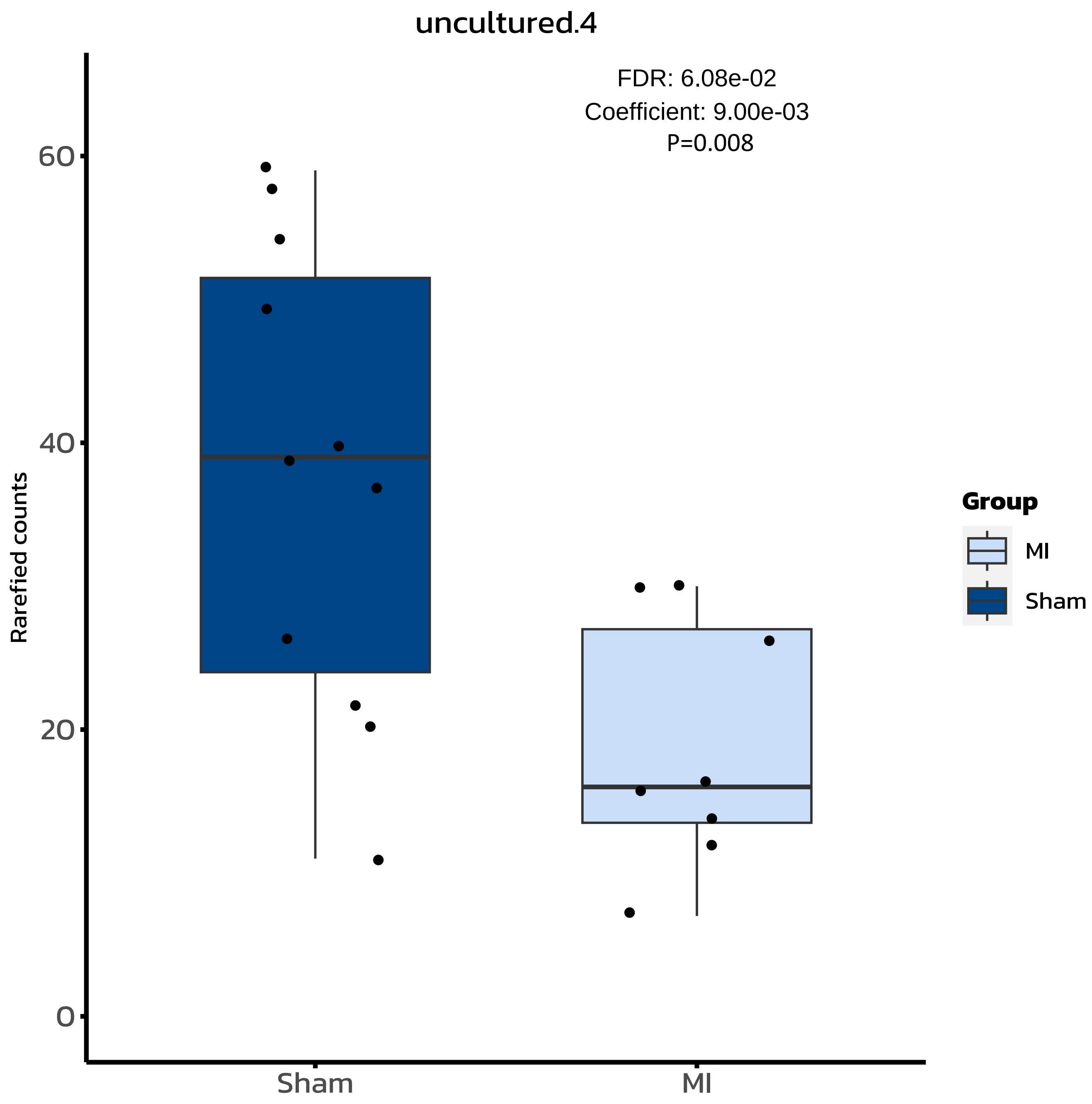
uncultured.4
FDR: 6.08e-02
Coefficient: 9.00e-03
P=0.008
Rarefied counts
0
20
40
60
Sham
MI
Group
MI
Sham

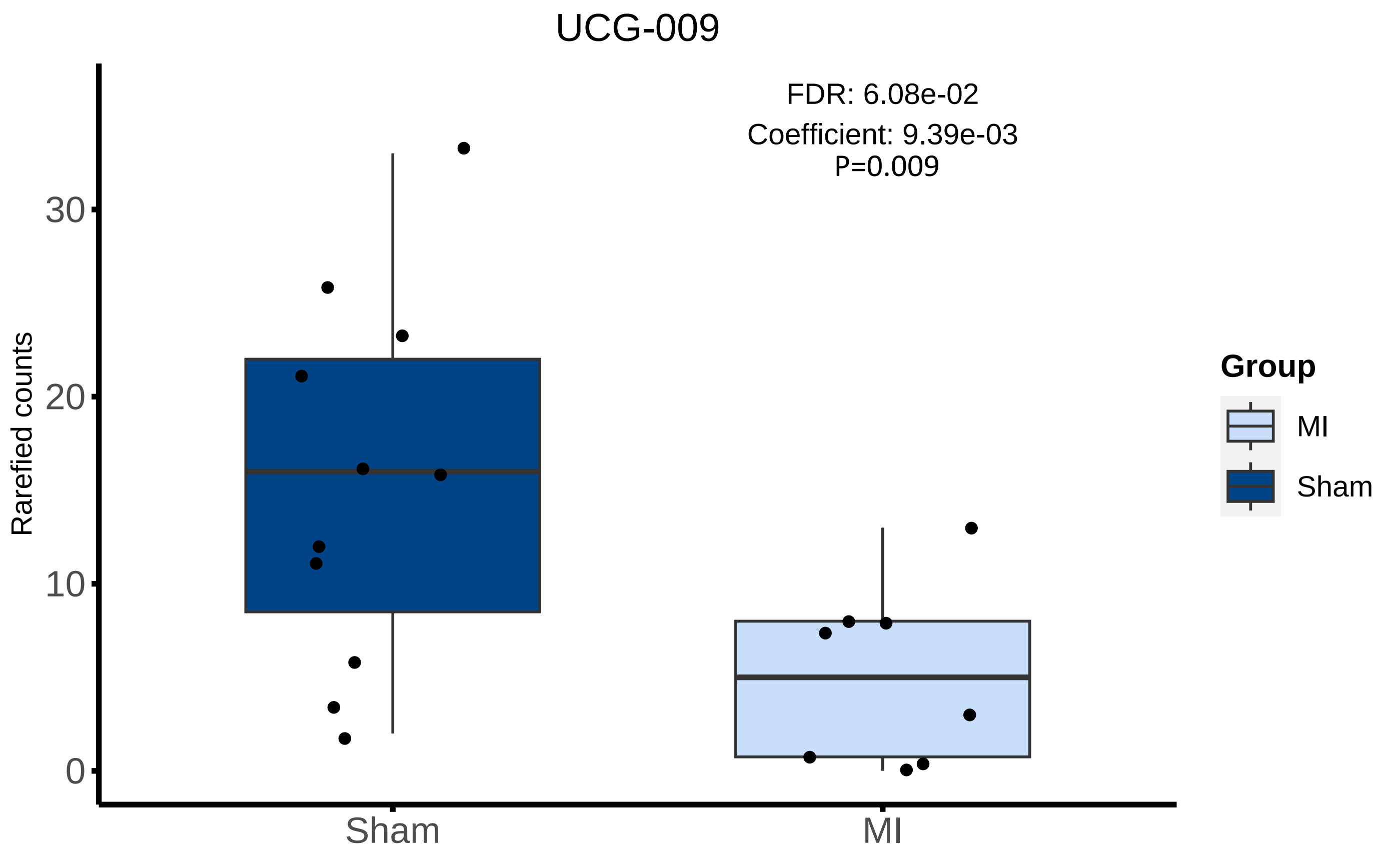
UCG-009
FDR: 6.08e-02
Coefficient: 9.39e-03
P=0.009
Rarefied counts
0
10
20
30
Sham
MI
Group
MI
Sham

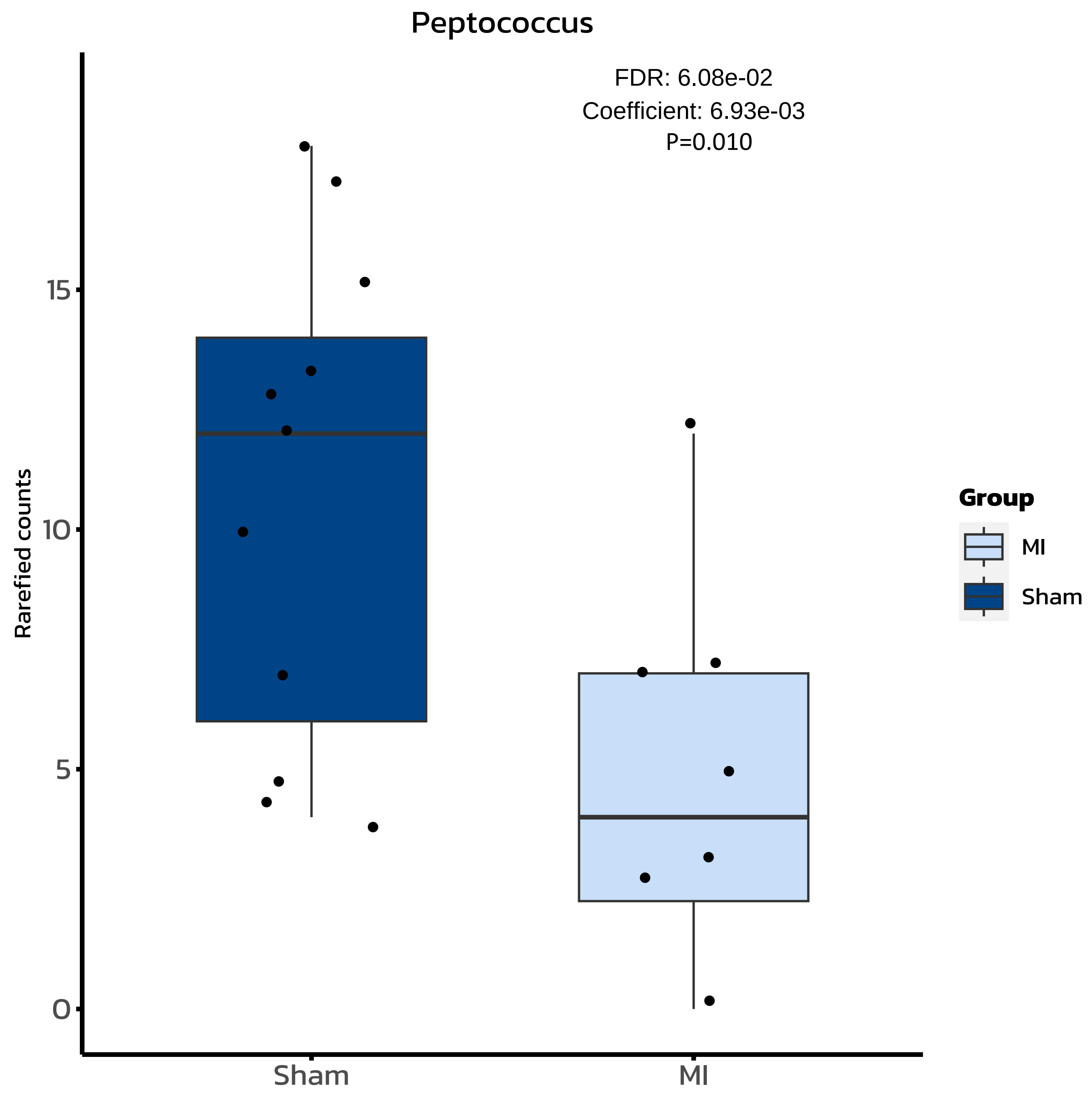

Peptococcus
FDR: 6.08e-02
Coefficient: 6.93e-03
P=0.010
Rarefied counts
0
5
10
15
Sham
MI
Group
MI
Sham

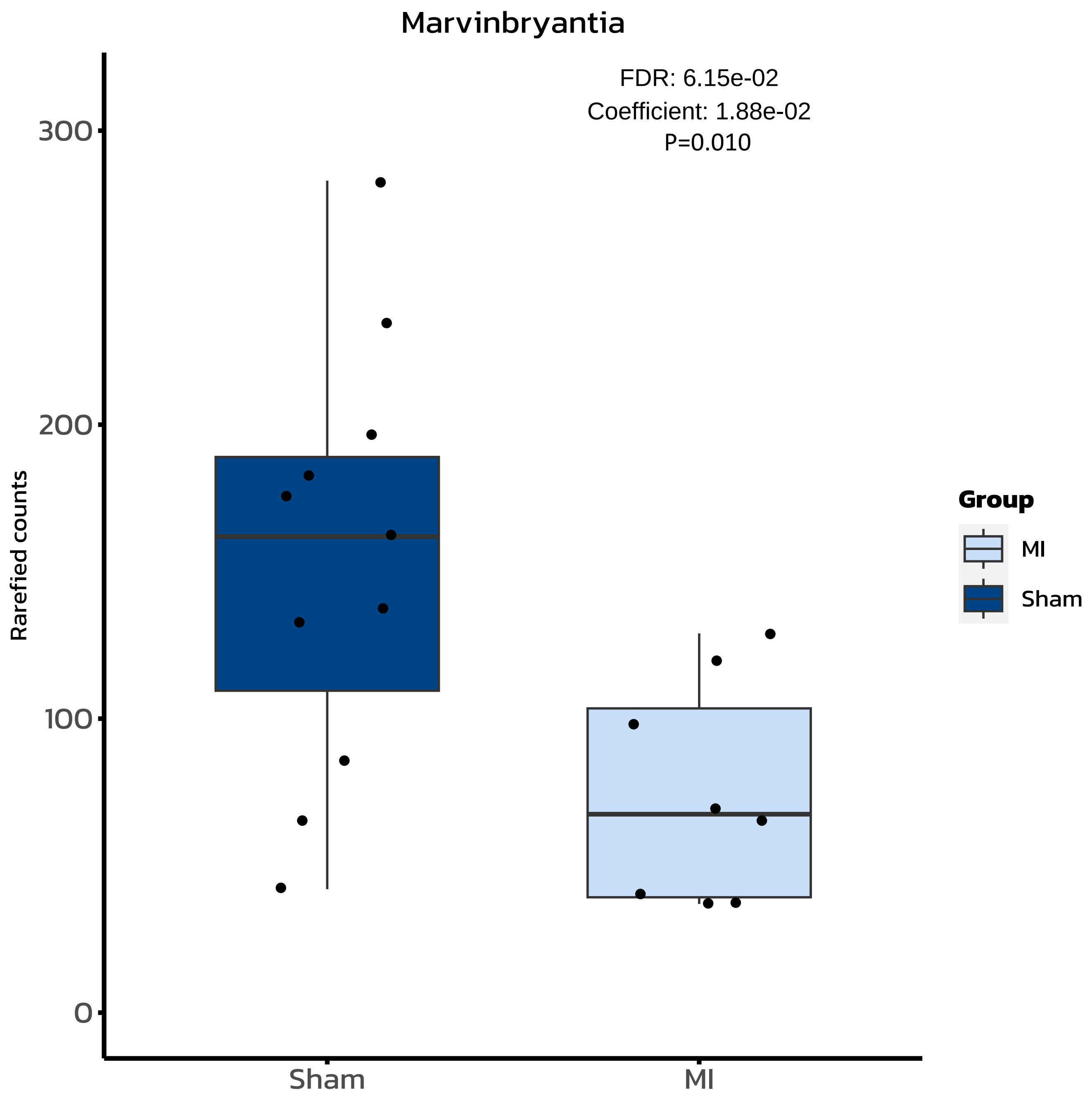
Marvinbryantia
FDR: 6.15e-02
Coefficient: 1.88e-02
P=0.010
300
200
100
0
Rarefied counts
Sham
MI
Group
MI
Sham

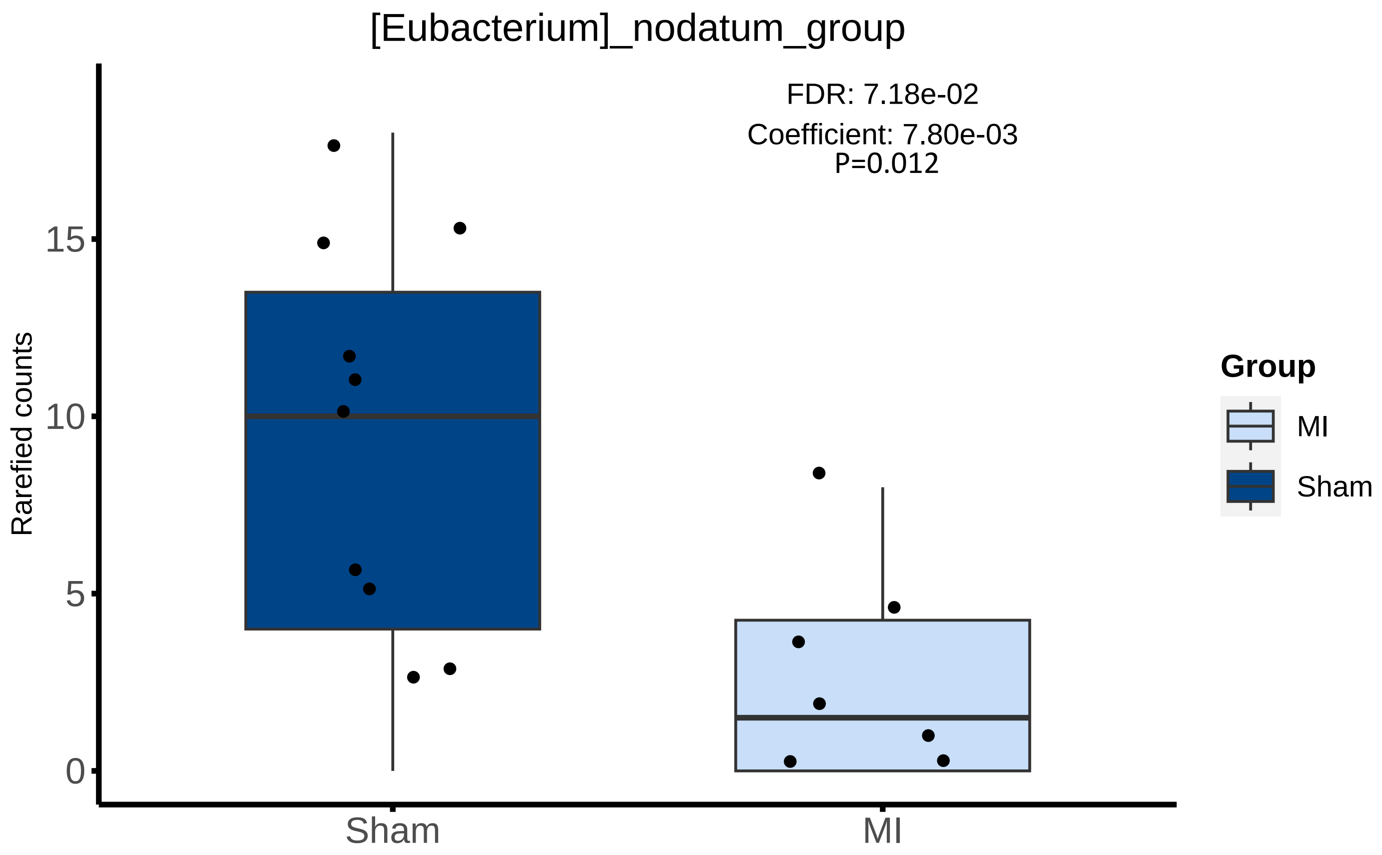

[Eubacterium]_nodatum_group
FDR: 7.18e-02
Coefficient: 7.80e-03
P=0.012
Rarefied counts
0
5
10
15
Sham
MI
Group
MI
Sham

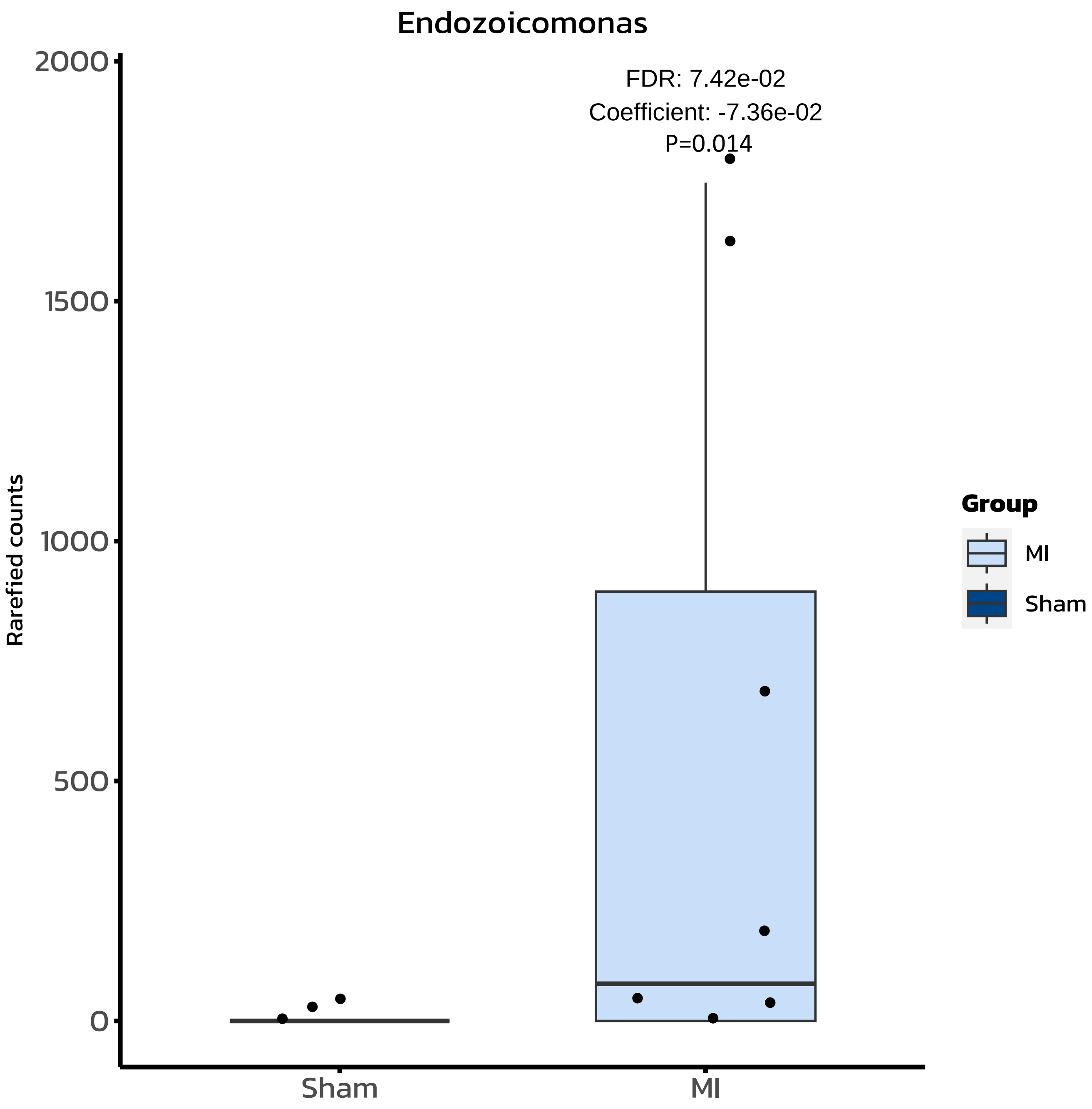

Endozoicomonas
FDR: 7.42e-02
Coefficient: -7.36e-02
P=0.014
Rarefied counts
2000
1500
1000
500
0
Sham
MI
Group
MI
Sham

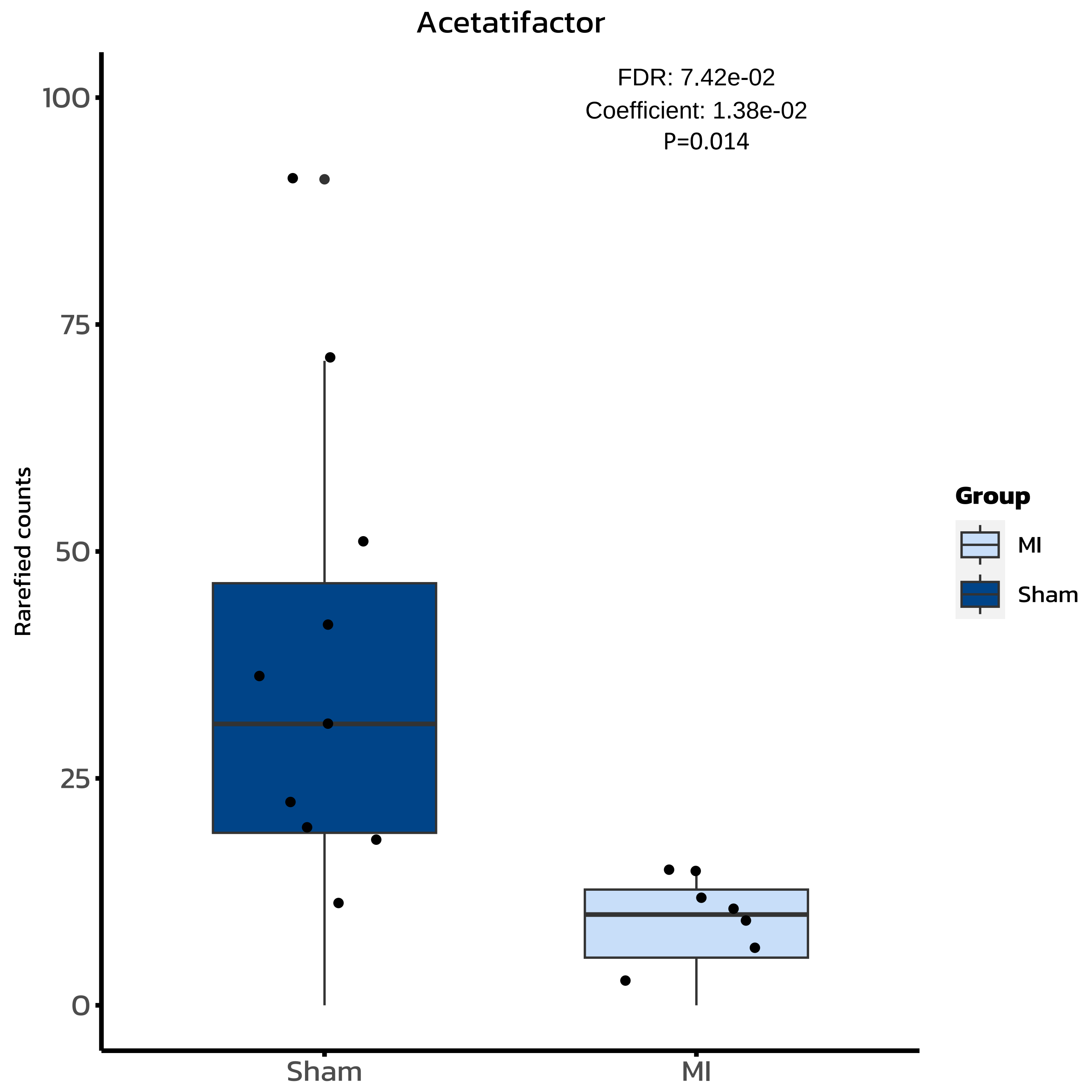
Acetatifactor
FDR: 7.42e-02
Coefficient: 1.38e-02
P=0.014
Rarefied counts
100
75
50
25
0
Sham
MI
Group
MI
Sham

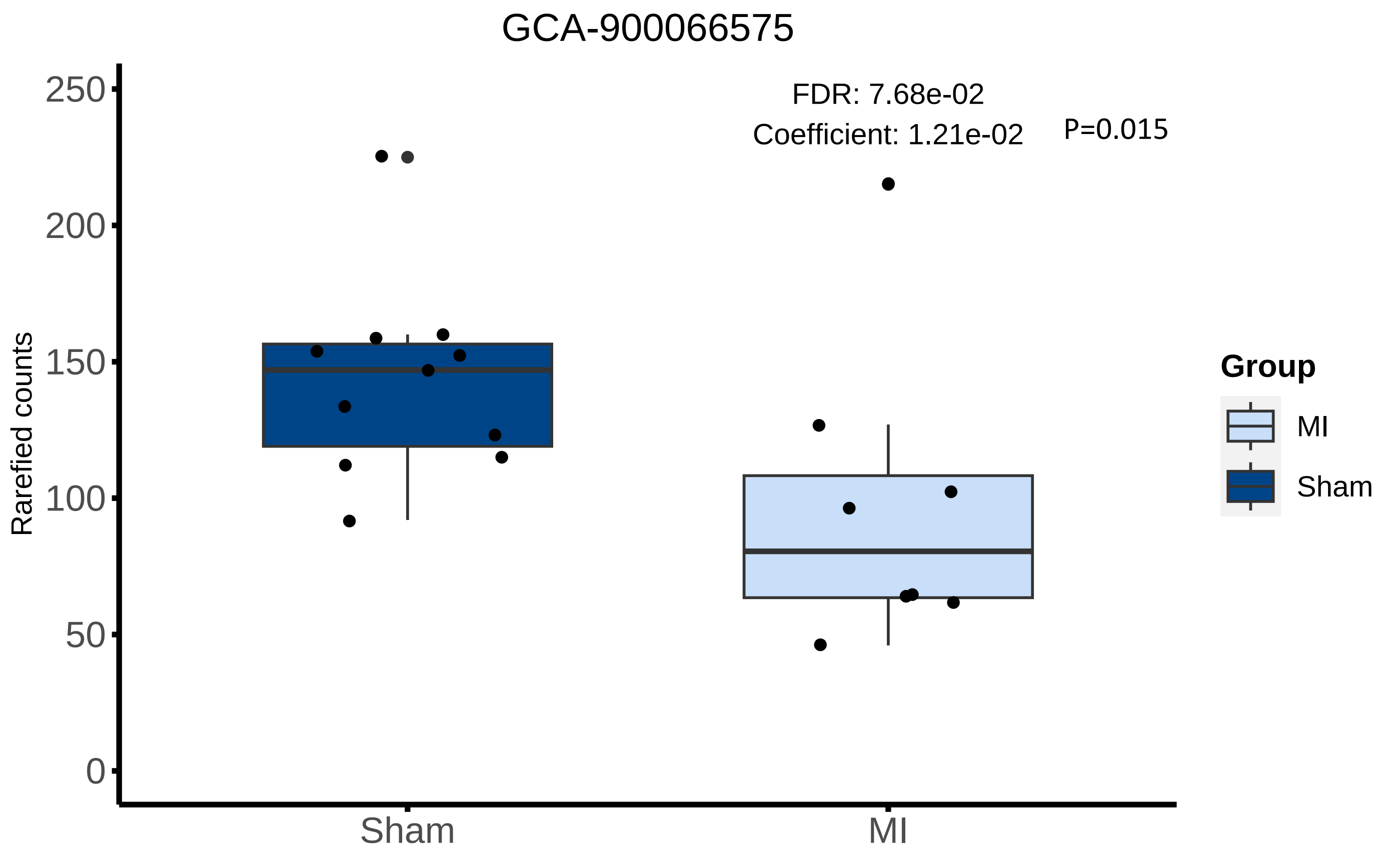
GCA-900066575
FDR: 7.68e-02
Coefficient: 1.21e-02
P=0.015
Rarefied counts
250
200
150
100
50
0
Sham
MI
Group
MI
Sham

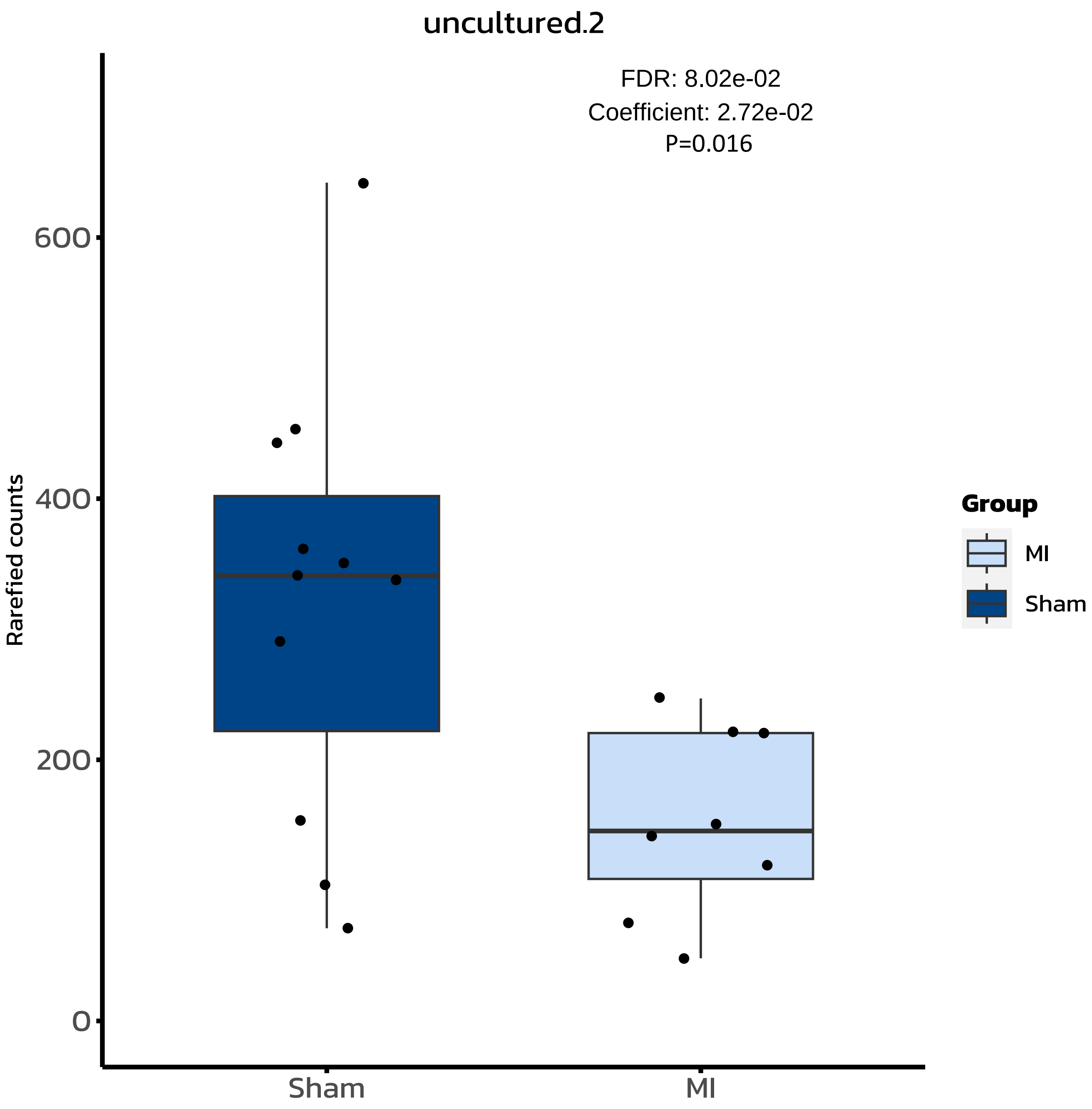
uncultured.2
FDR: 8.02e-02
Coefficient: 2.72e-02
P=0.016
Rarefied counts
600
400
200
0
Sham
MI
Group
MI
Sham

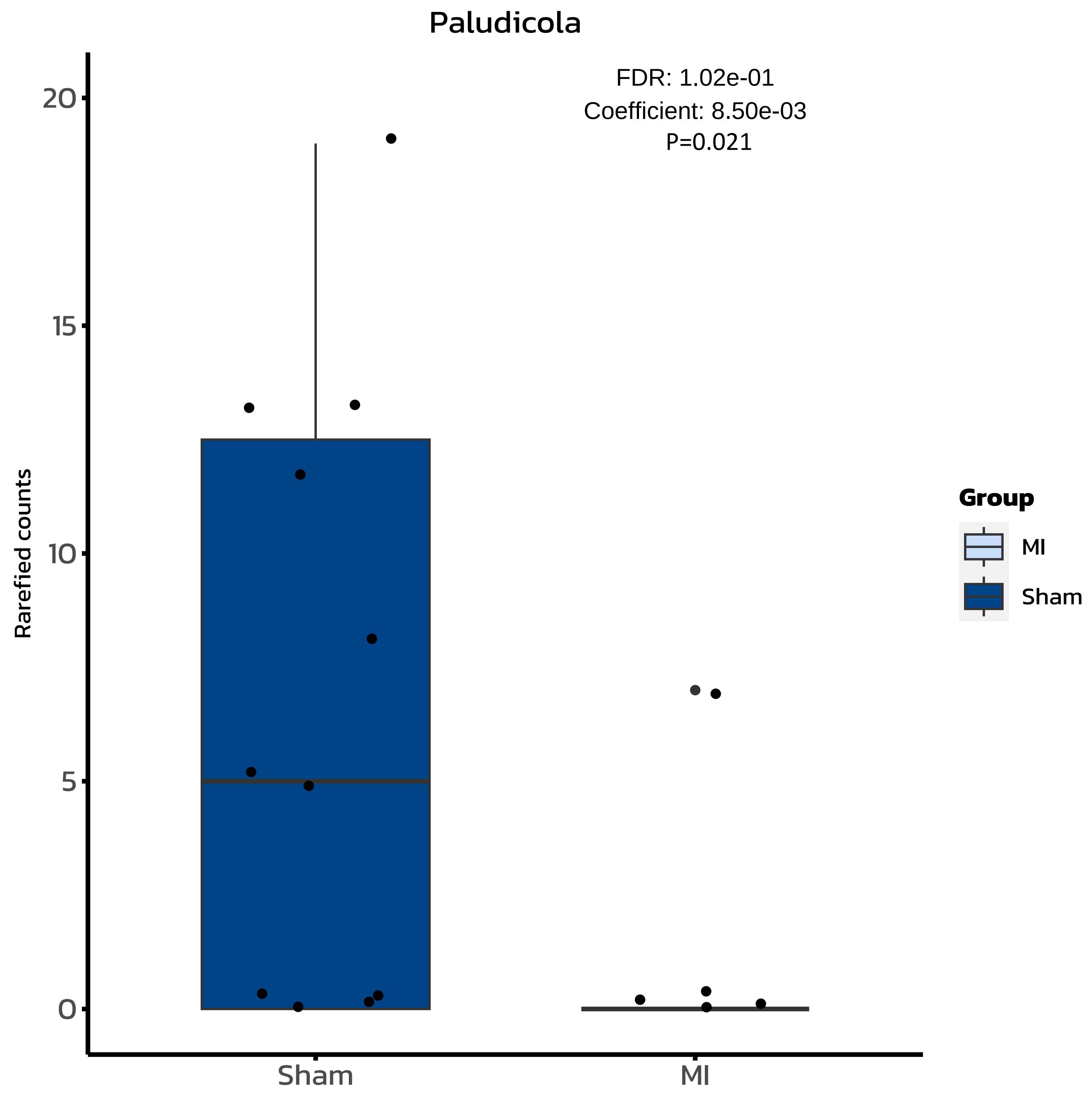
Paludicola
FDR: 1.02e-01
Coefficient: 8.50e-03
P=0.021
Rarefied counts
20
15
10
5
0
Sham
MI
Group
MI
Sham

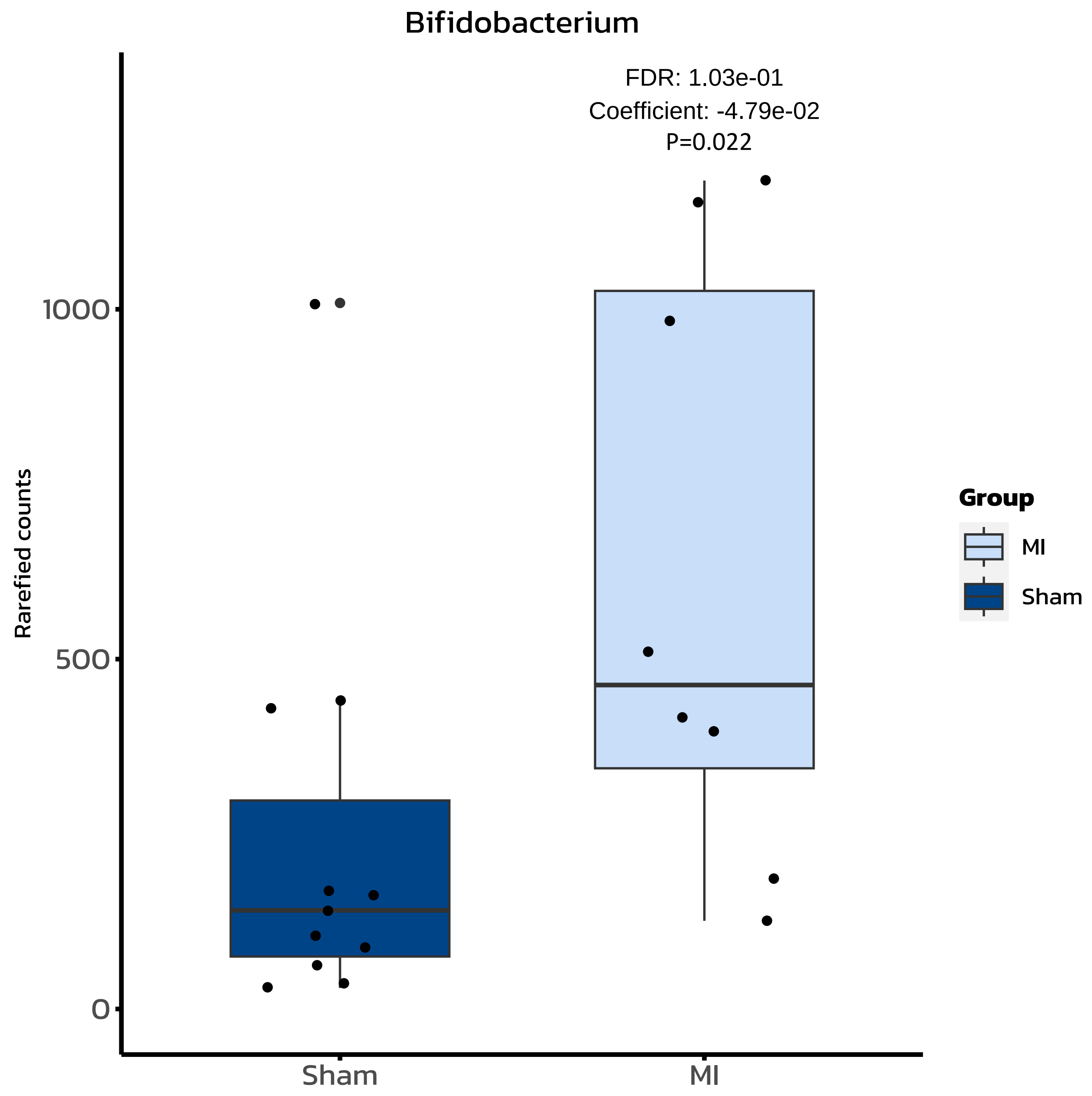
Bifidobacterium
FDR: 1.03e-01
Coefficient: -4.79e-02
P=0.022
Rarefied counts
1000
500
0
Sham
MI
Group
MI
Sham

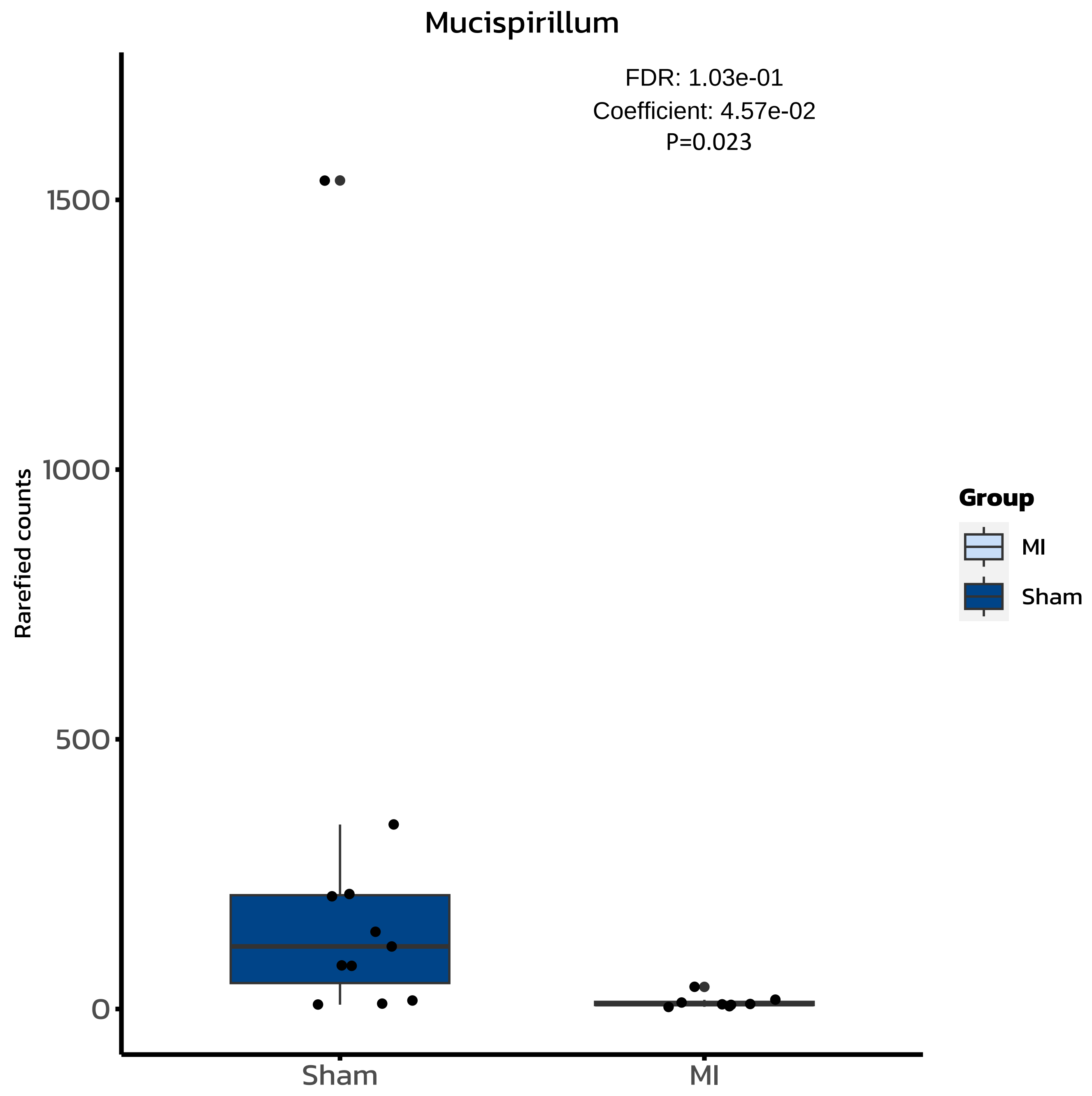
Mucispirillum
FDR: 1.03e-01
Coefficient: 4.57e-02
P=0.023
Rarefied counts
1500
1000
500
0
Sham
MI
Group
MI
Sham

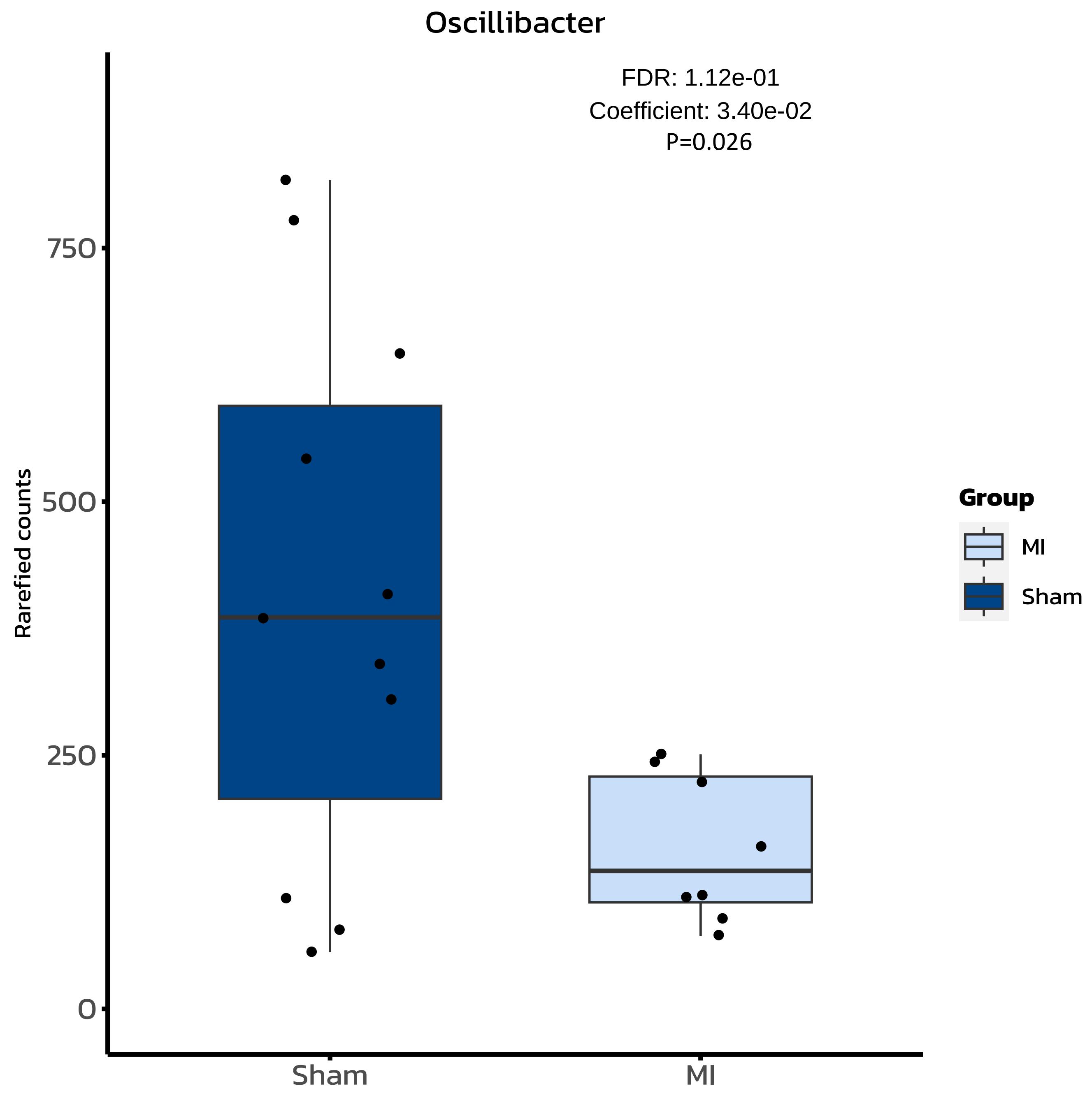

Oscillibacter
FDR: 1.12e-01
Coefficient: 3.40e-02
P=0.026
Rarefied counts
750
500
250
0
Sham
MI
Group
MI
Sham

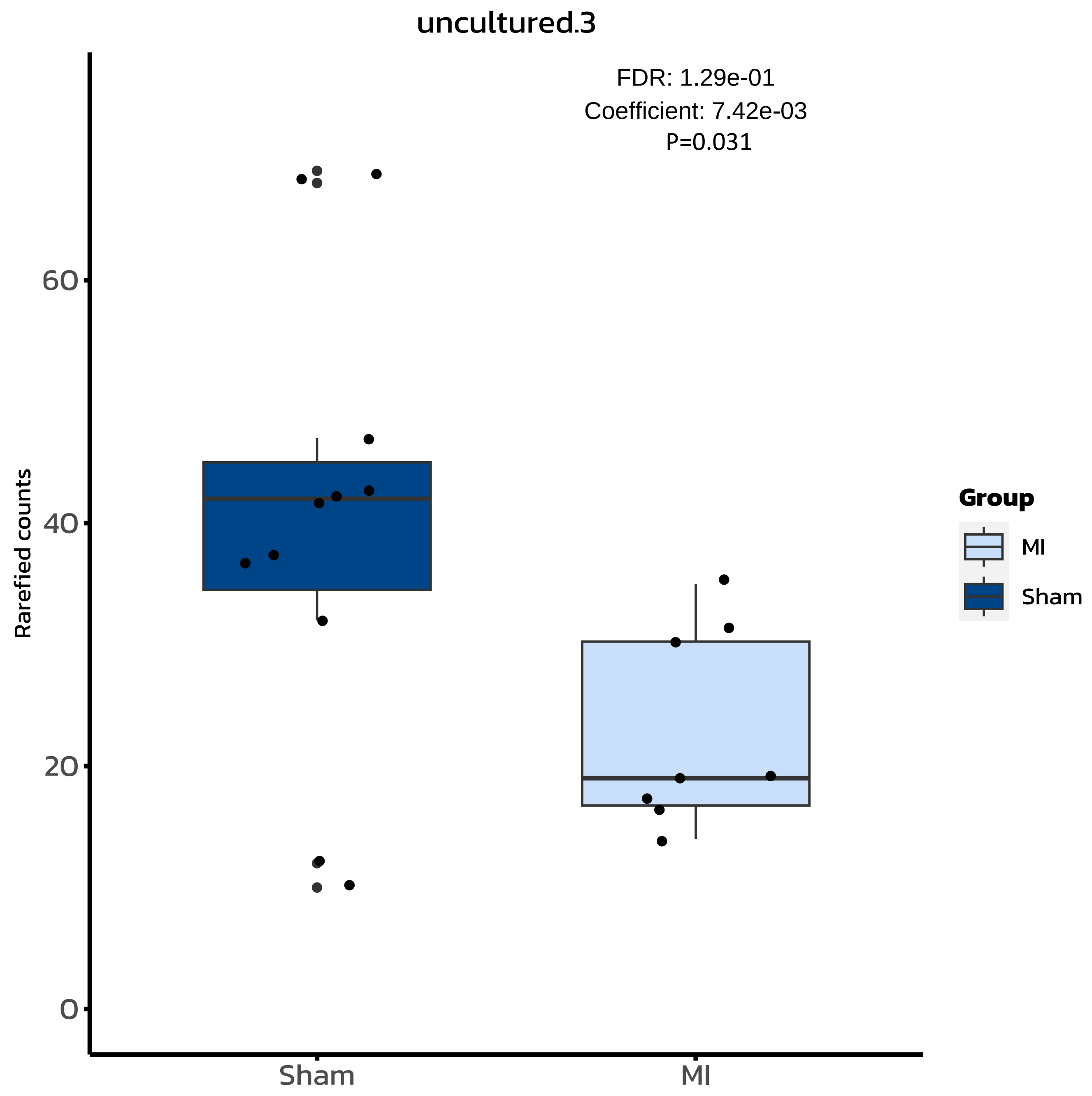

uncultured.3
FDR: 1.29e-01
Coefficient: 7.42e-03
P=0.031
Rarefied counts
60
40
20
0
Sham
MI
Group
MI
Sham

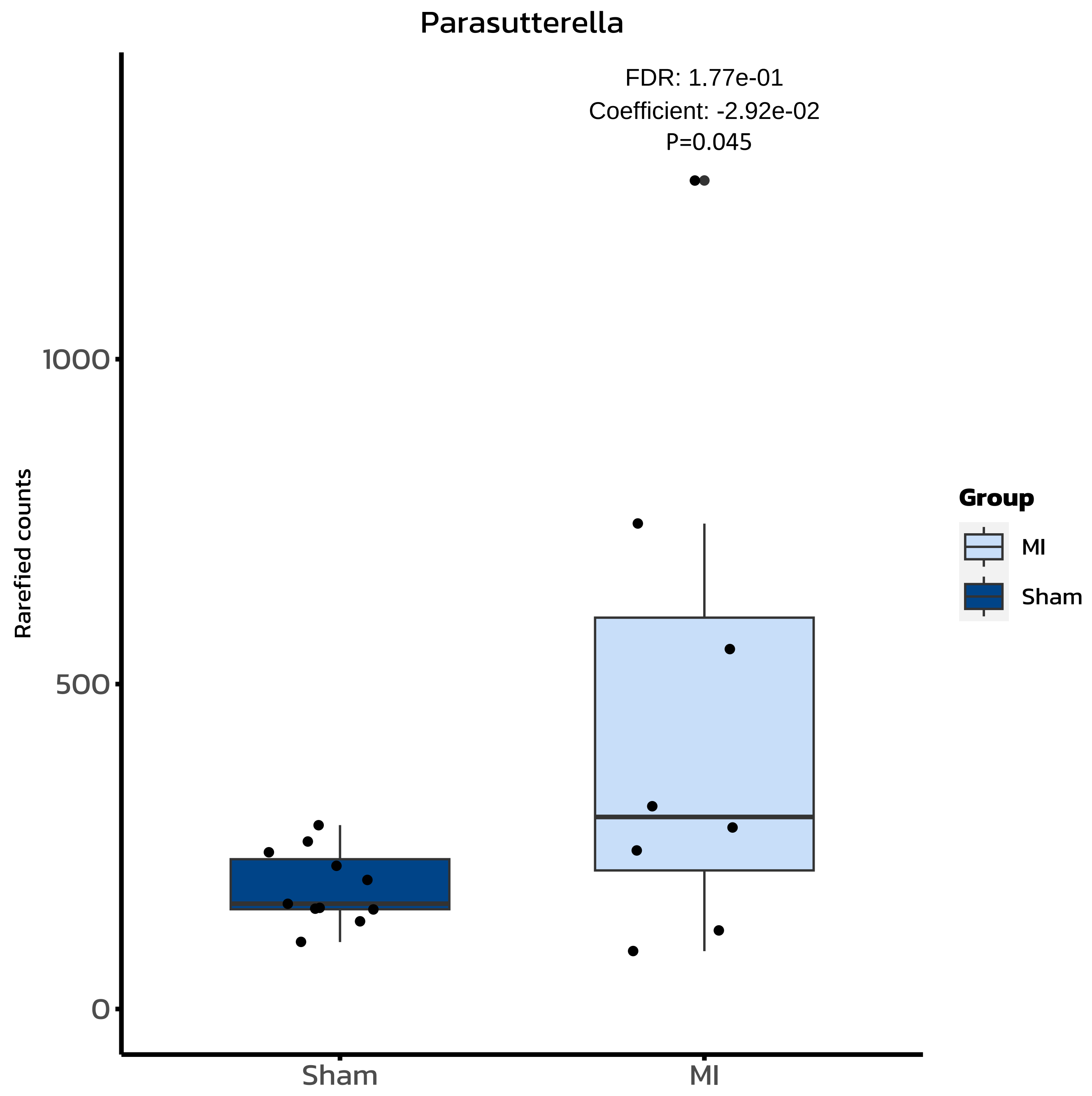
Parasutterella
FDR: 1.77e-01
Coefficient: -2.92e-02
P=0.045
Rarefied counts
1000
500
0
Sham
MI
Group
MI
Sham

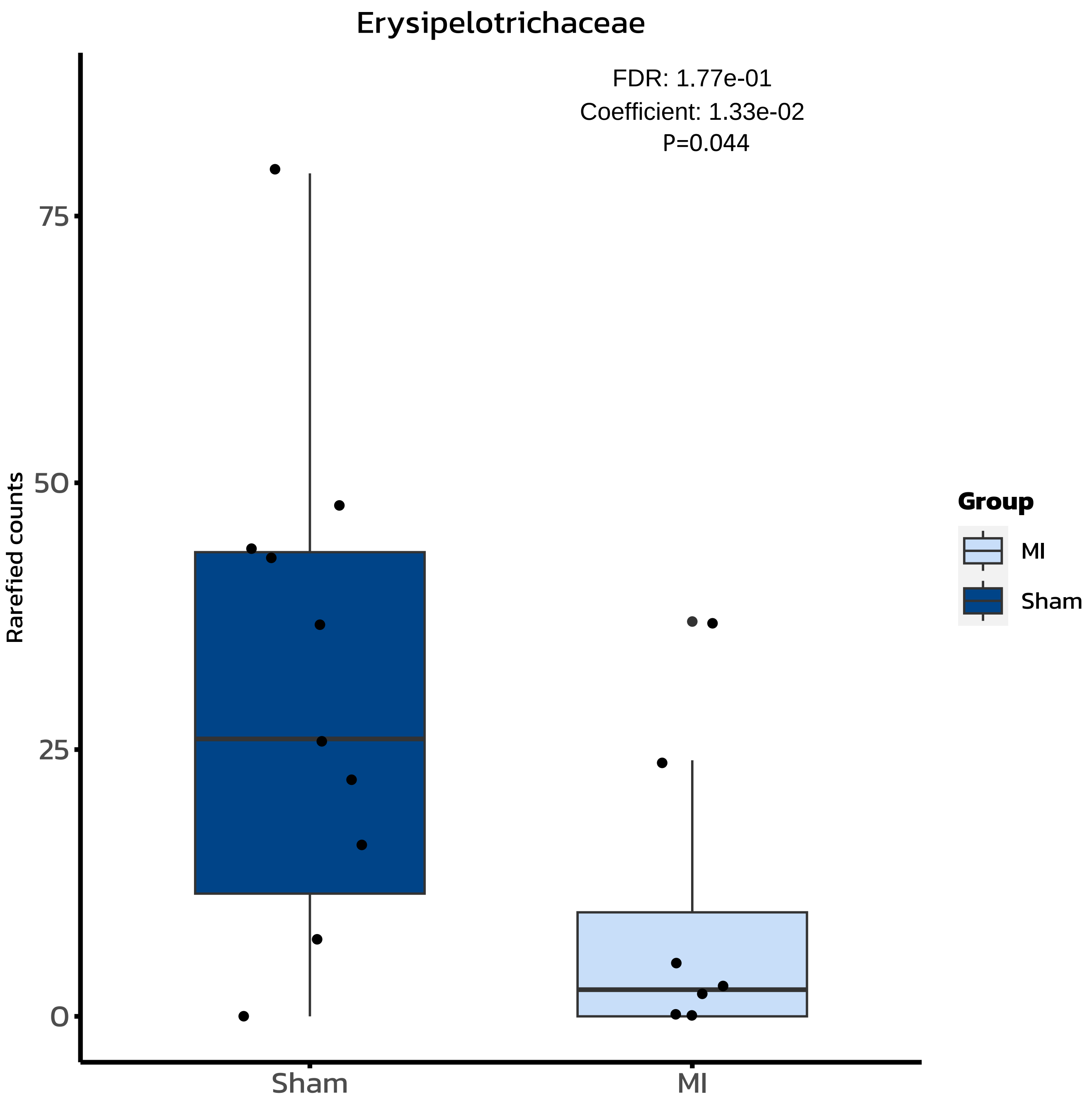

Erysipelotrichaceae
FDR: 1.77e-01
Coefficient: 1.33e-02
P=0.044
Rarefied counts
75
50
25
0
Sham
MI
Group
MI
Sham

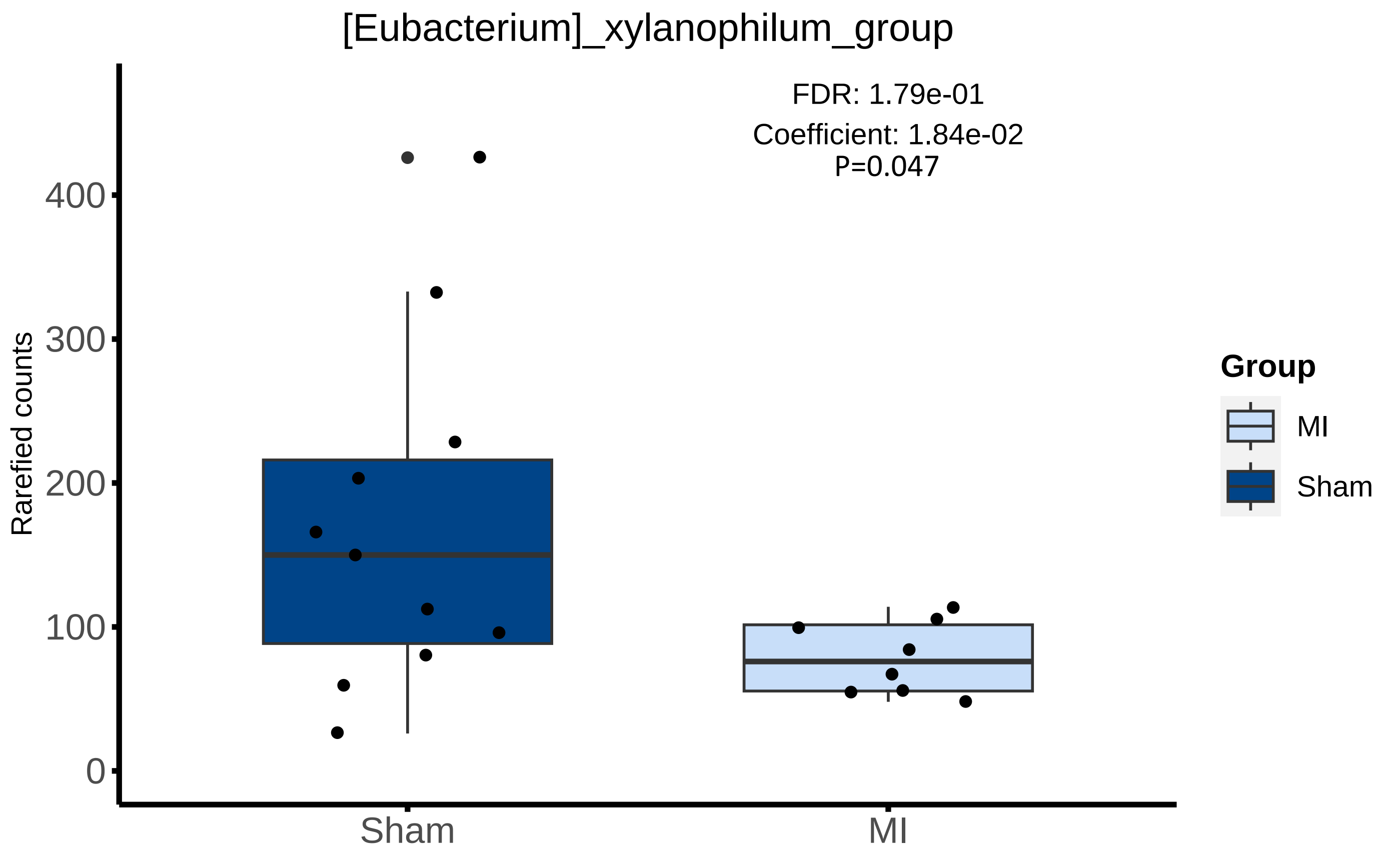

[Eubacterium]_xylanophilum_group
FDR: 1.79e-01
Coefficient: 1.84e-02
P=0.047
Rarefied counts
400
300
200
100
0
Sham
MI
Group
MI
Sham
