# Supplementary material for: Heart failure-induced microbial dysbiosis contributes to colonic tumour formation in mice
Source: Cardiovasc Res. 2024 Feb 24;120(6):612–22. doi: 10.1093/cvr/cvae038 (PMC11074794; doi:10.1093/cvr/cvae038)
Supplement: cvae038_Supplementary_Data [file cvae038_supplementary_data.zip › Supplemental File 6.pdf]

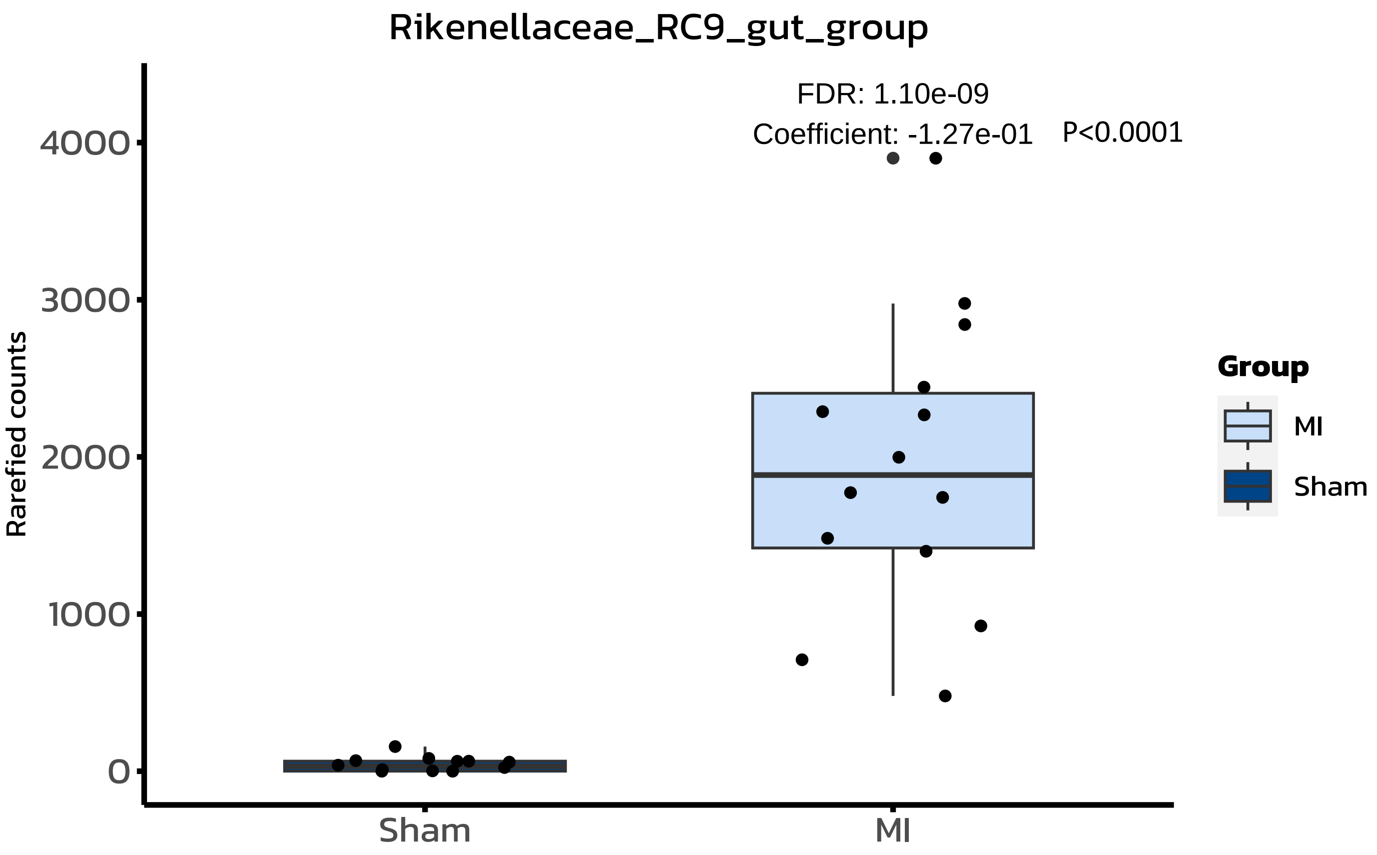
Rikenellaceae_RC9_gut_group
FDR: 1.10e-09
Coefficient: -1.27e-01
P<0.0001
Rarefied counts
4000
3000
2000
1000
0
Sham
MI
Group
MI
Sham

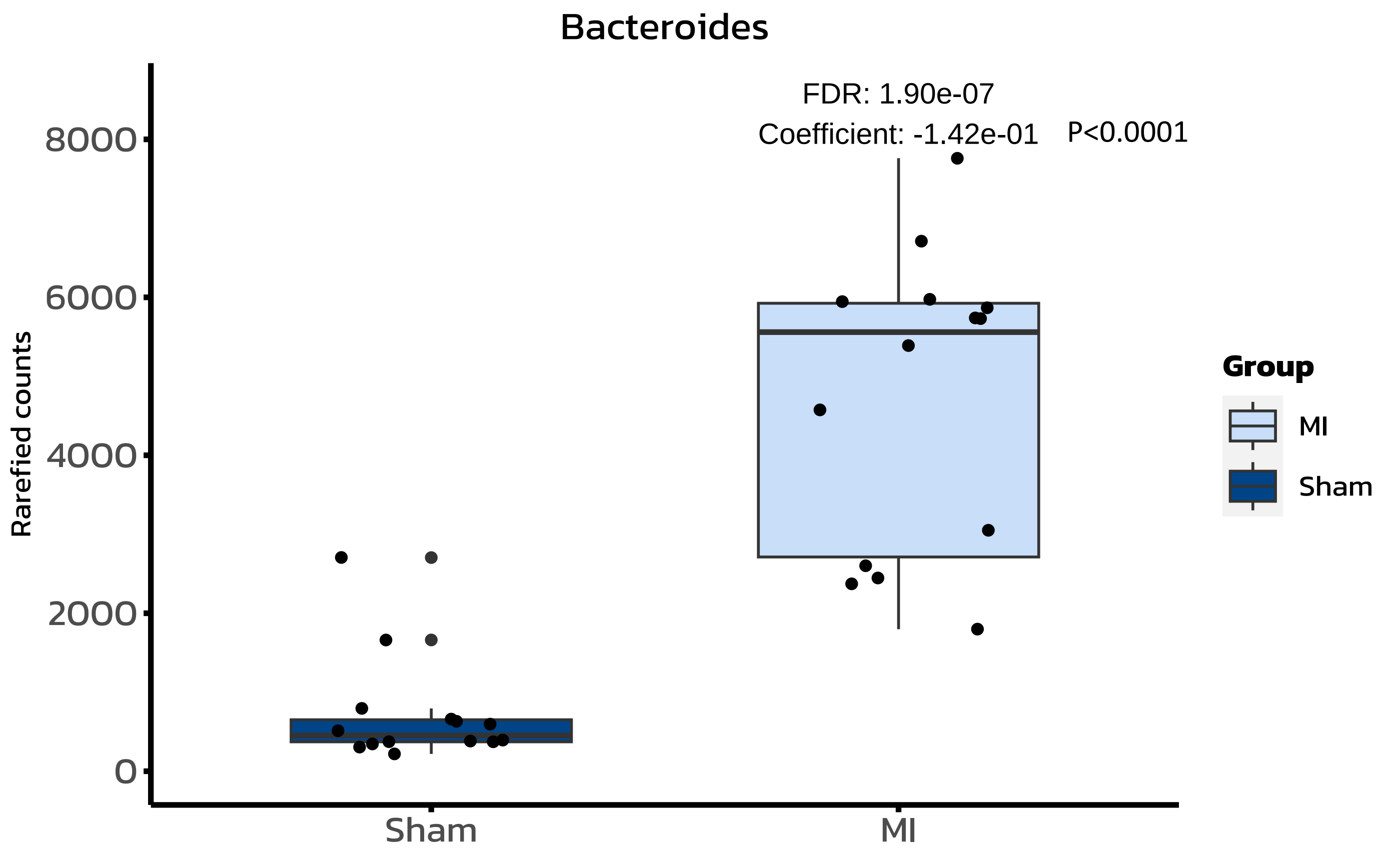

Bacteroides
FDR: 1.90e-07
Coefficient: -1.42e-01
P<0.0001
Rarefied counts
8000
6000
4000
2000
0
Sham
MI
Group
MI
Sham

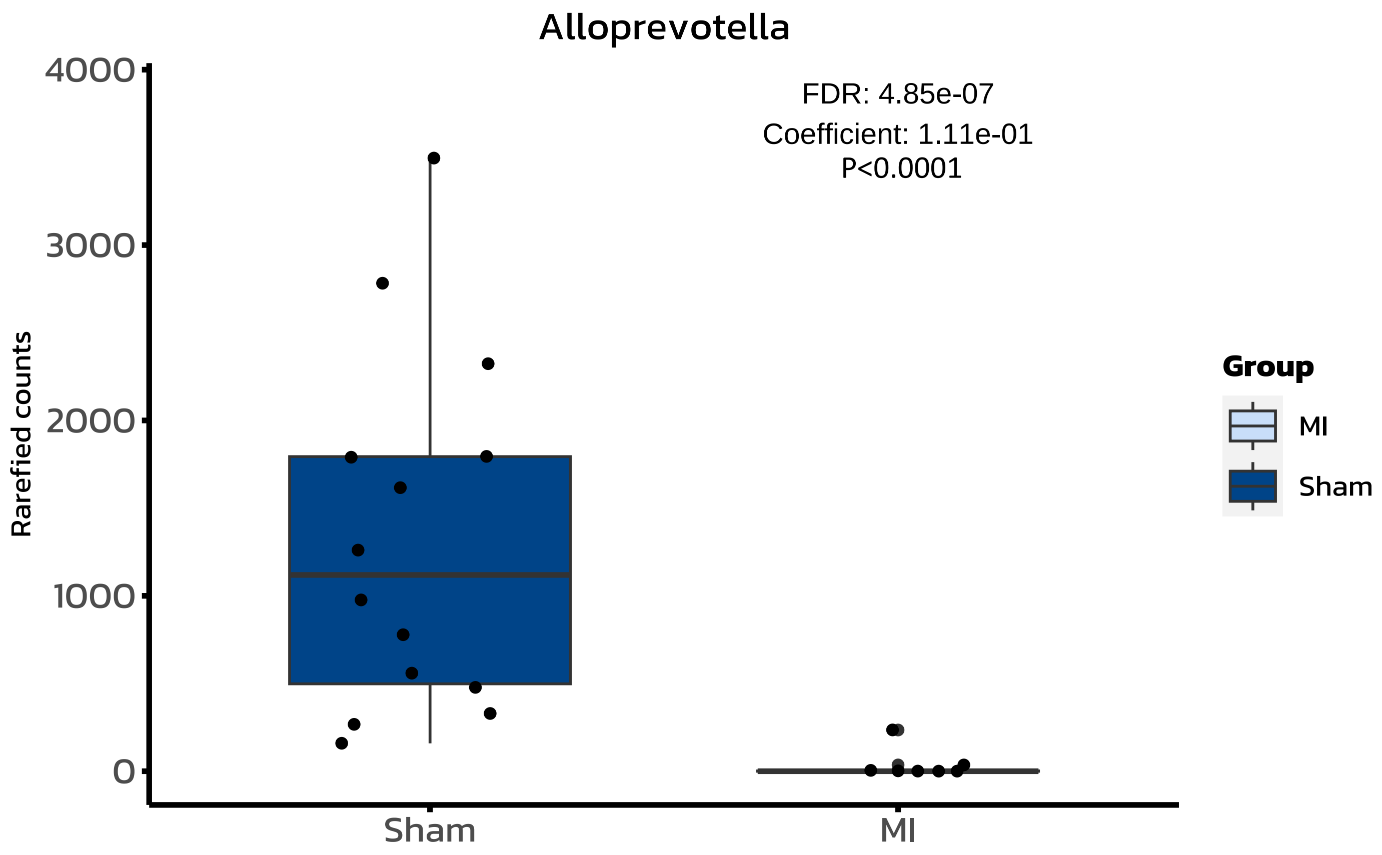
Alloprevotella
FDR: 4.85e-07
Coefficient: 1.11e-01
P<0.0001
Rarefied counts
4000
3000
2000
1000
0
Sham
MI
Group
MI
Sham

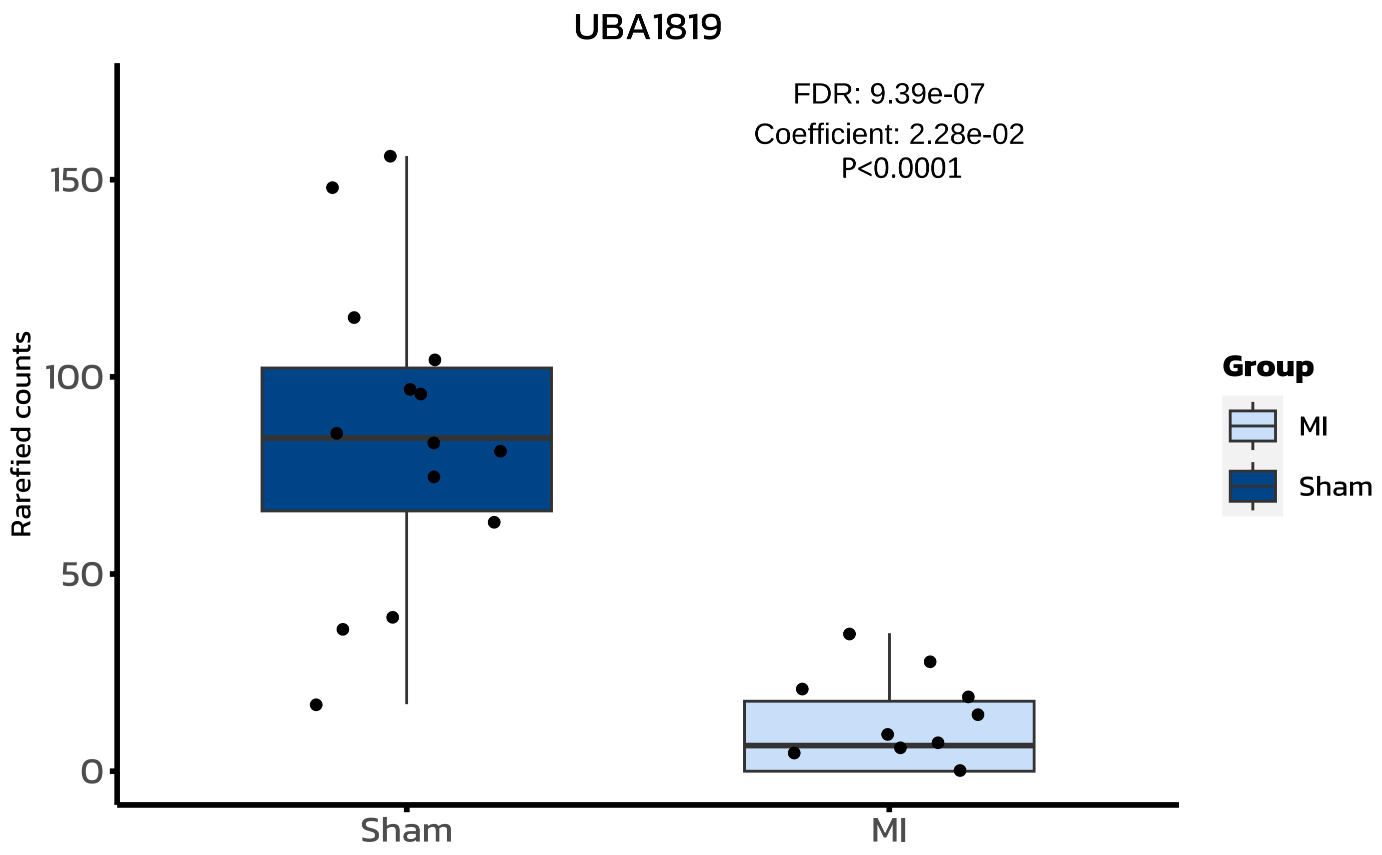
UBA1819
FDR: 9.39e-07
Coefficient: 2.28e-02
P<0.0001
Rarefied counts
150
100
50
0
Sham
MI
Group
MI
Sham

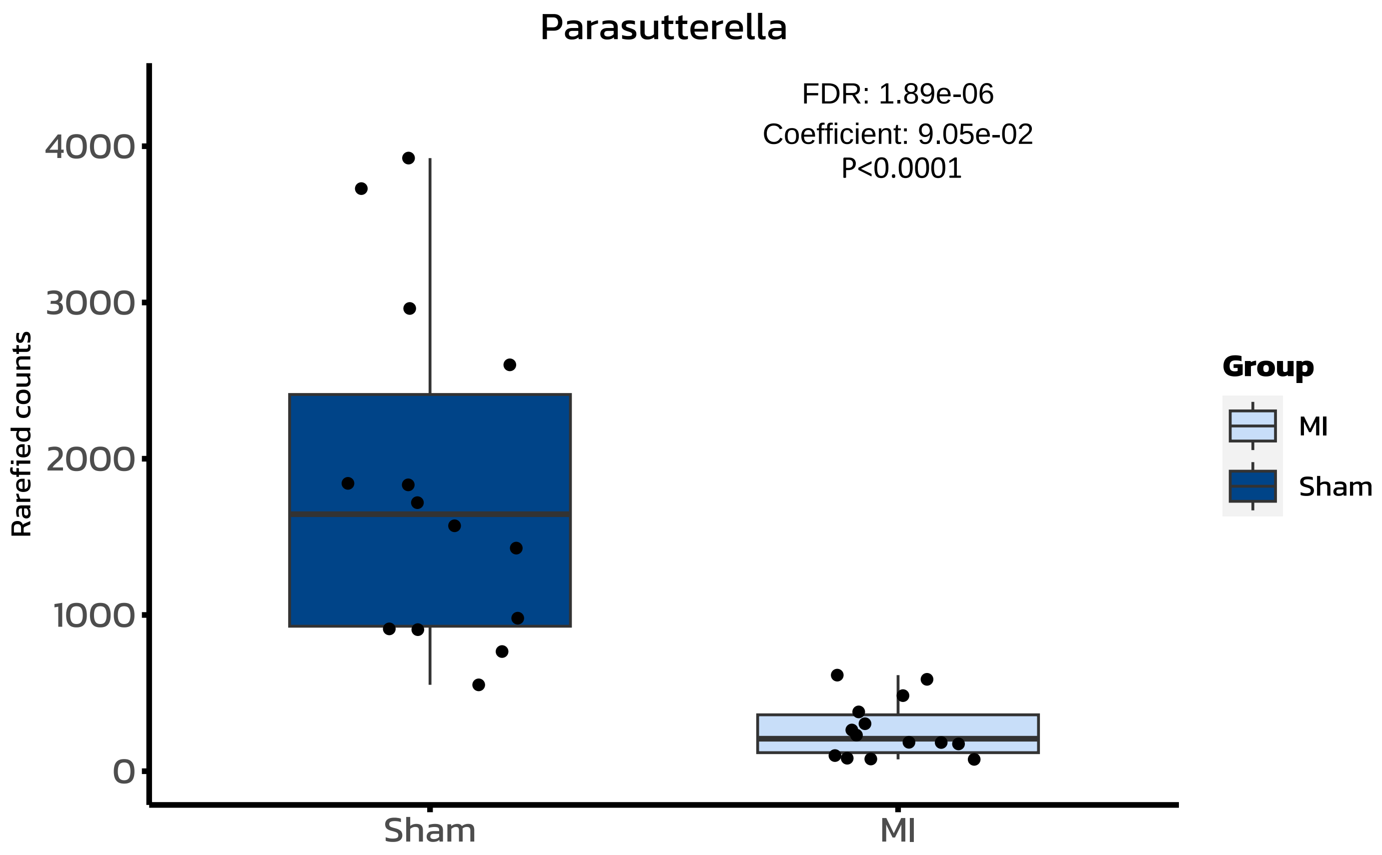

Parasutterella
FDR: 1.89e-06
Coefficient: 9.05e-02
P<0.0001
Rarefied counts
4000
3000
2000
1000
0
Sham
MI
Group
MI
Sham

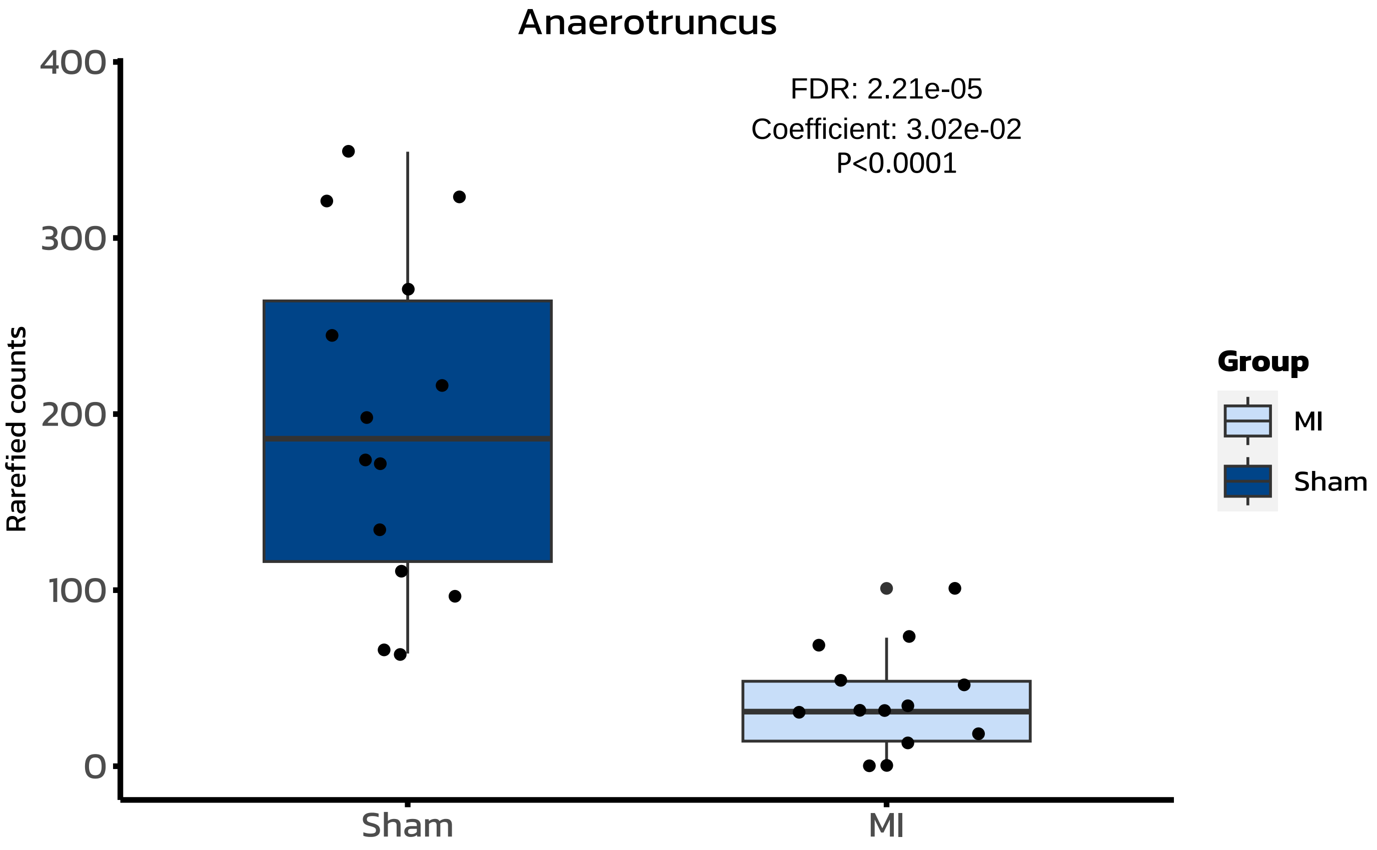
Anaerotruncus
FDR: 2.21e-05
Coefficient: 3.02e-02
P<0.0001
400
300
200
100
0
Rarefied counts
Sham
MI
Group
MI
Sham

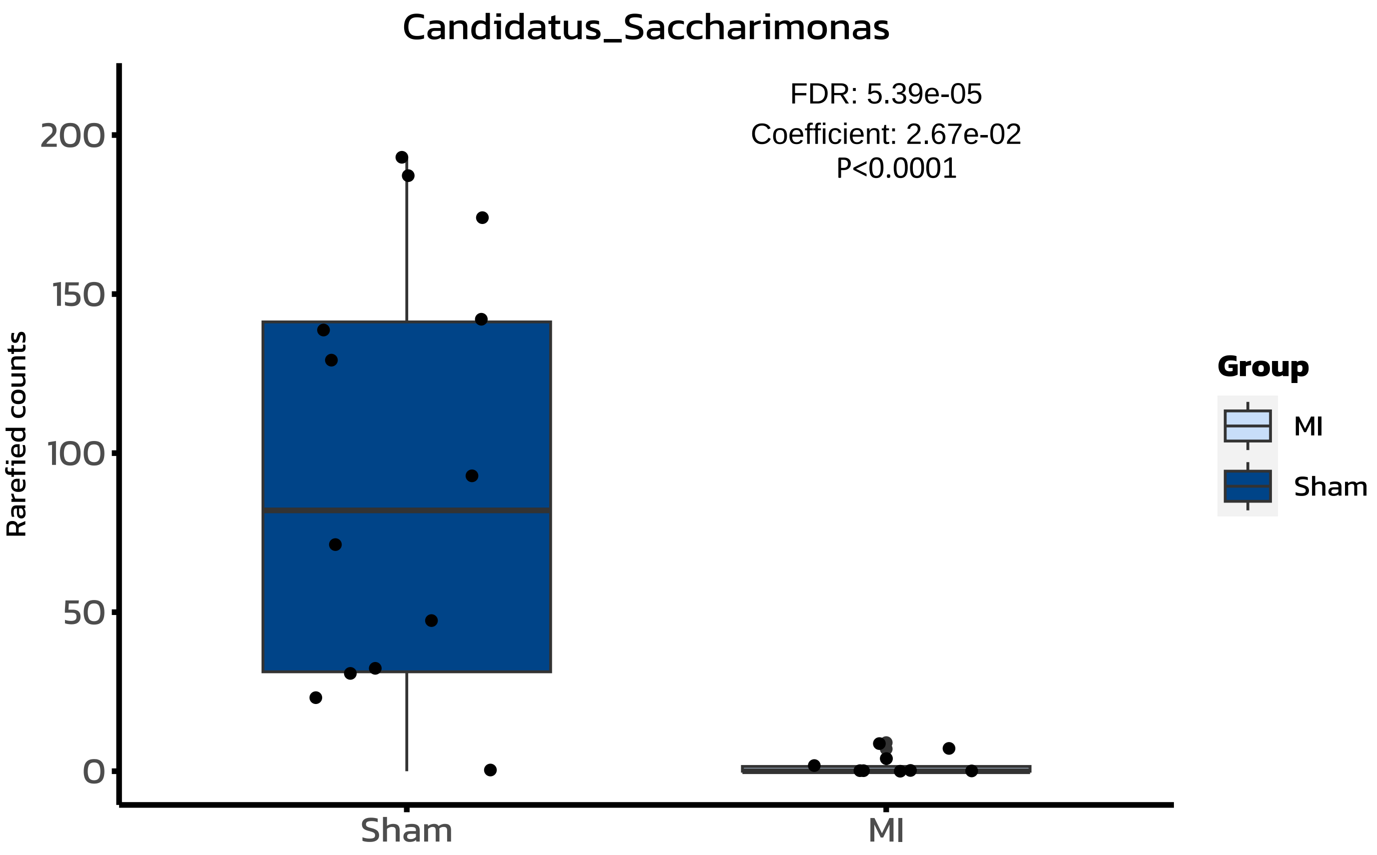
Candidatus_Saccharimonas
FDR: 5.39e-05
Coefficient: 2.67e-02
P<0.0001
Rarefied counts
200
150
100
50
0
Sham
MI
Group
MI
Sham

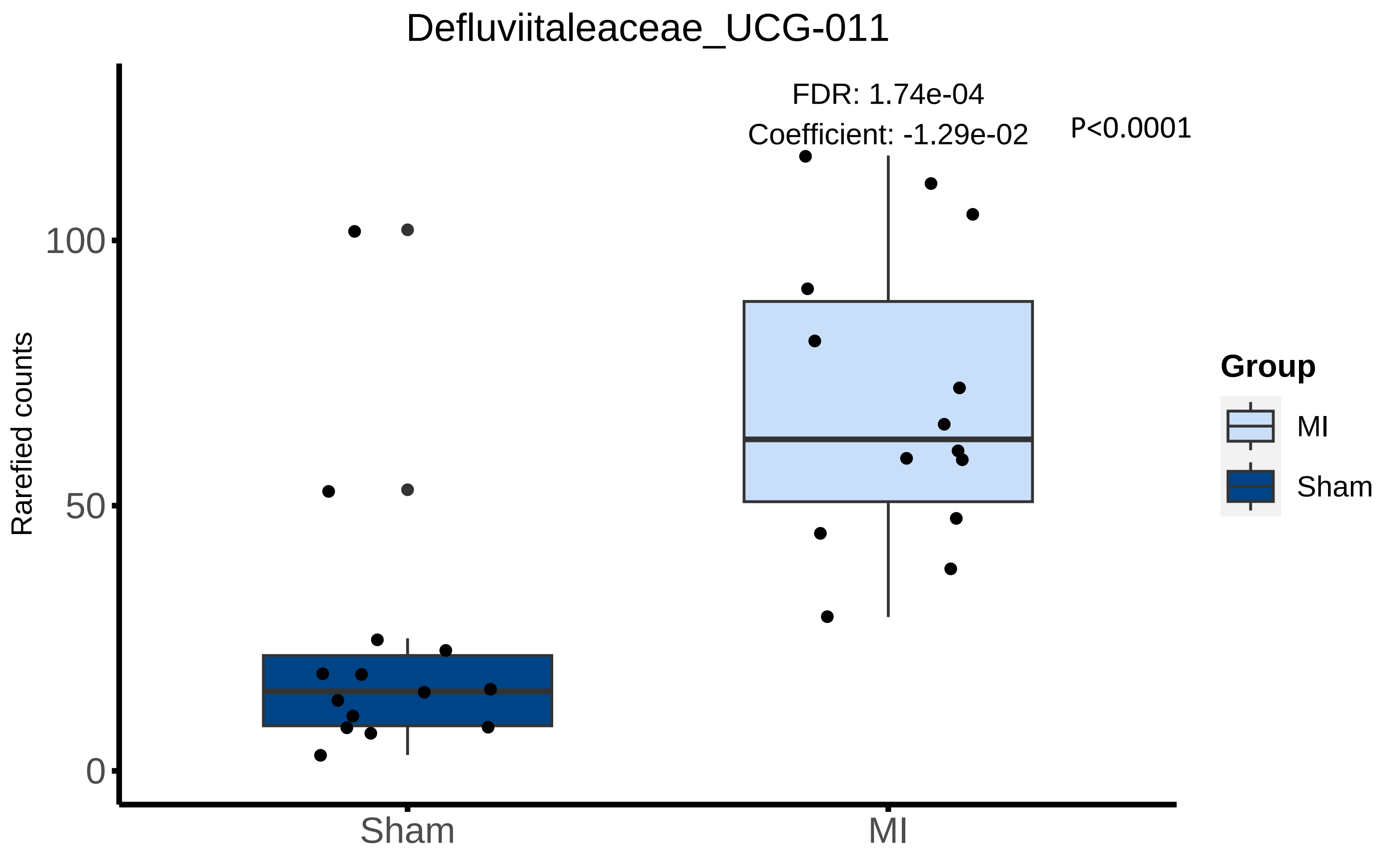
Defluviitaleaceae_UCG-011
FDR: 1.74e-04
Coefficient: -1.29e-02
P<0.0001
Rarefied counts
100
50
0
Sham
MI
Group
MI
Sham

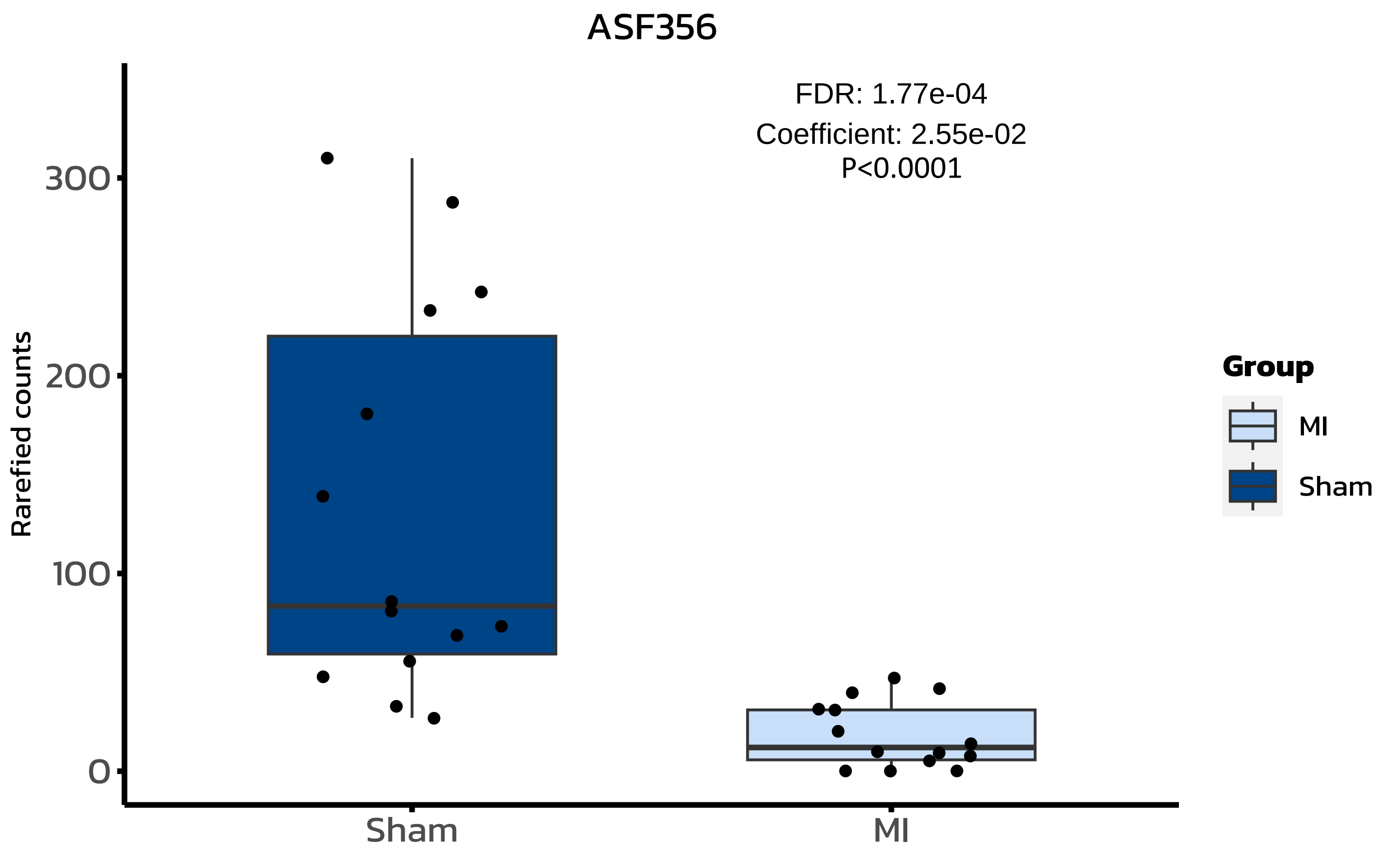

ASF356
FDR: 1.77e-04
Coefficient: 2.55e-02
P<0.0001
Rarefied counts
300
200
100
0
Sham
MI
Group
MI
Sham

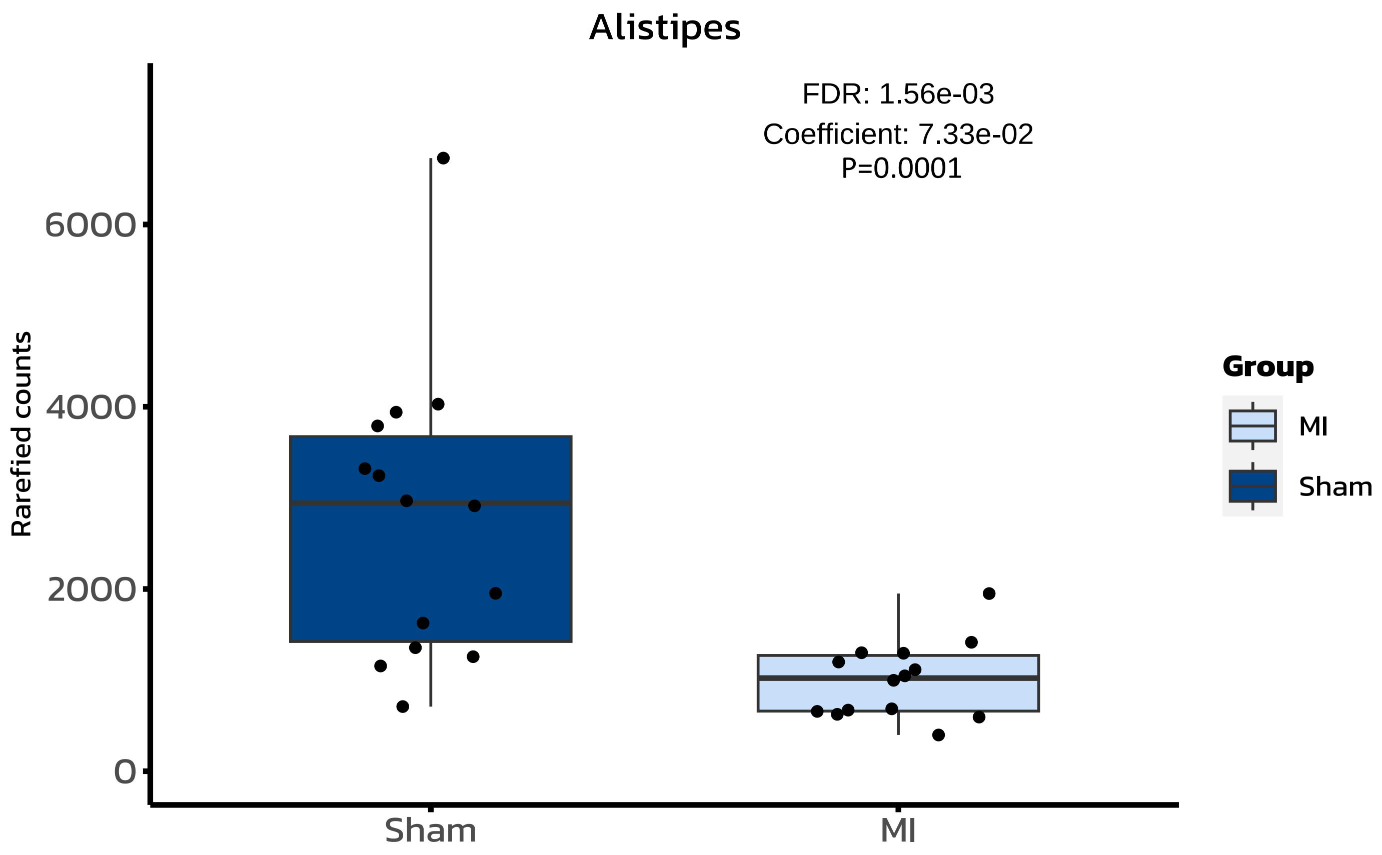
Alistipes
FDR: 1.56e-03
Coefficient: 7.33e-02
P=0.0001
Rarefied counts
6000
4000
2000
0
Sham
MI
Group
MI
Sham

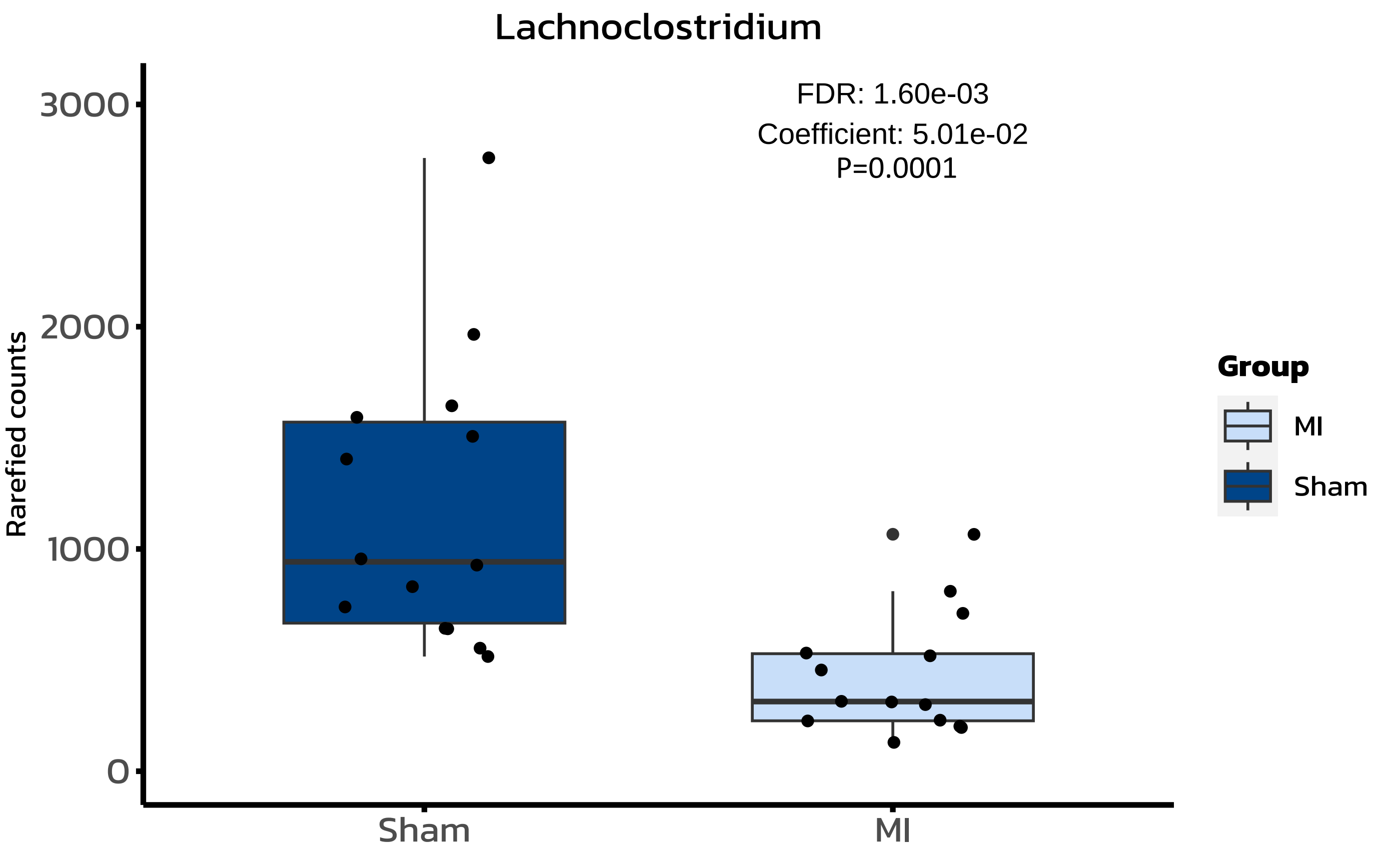

Lachnoclostridium
FDR: 1.60e-03
Coefficient: 5.01e-02
P=0.0001
Rarefied counts
3000
2000
1000
0
Sham
MI
Group
MI
Sham

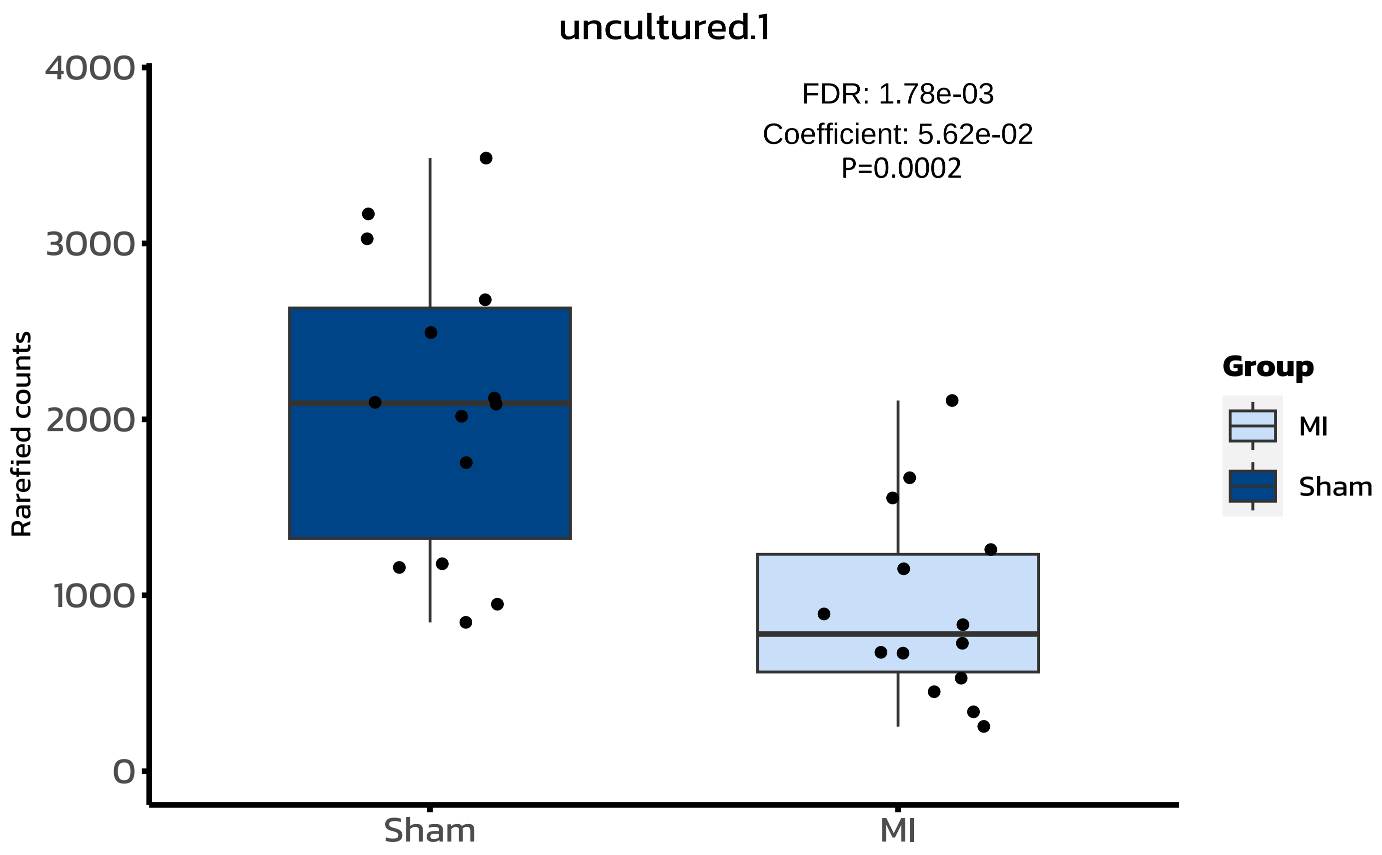
uncultured.1
FDR: 1.78e-03
Coefficient: 5.62e-02
P=0.0002
Rarefied counts
4000
3000
2000
1000
0
Sham
MI
Group
MI
Sham

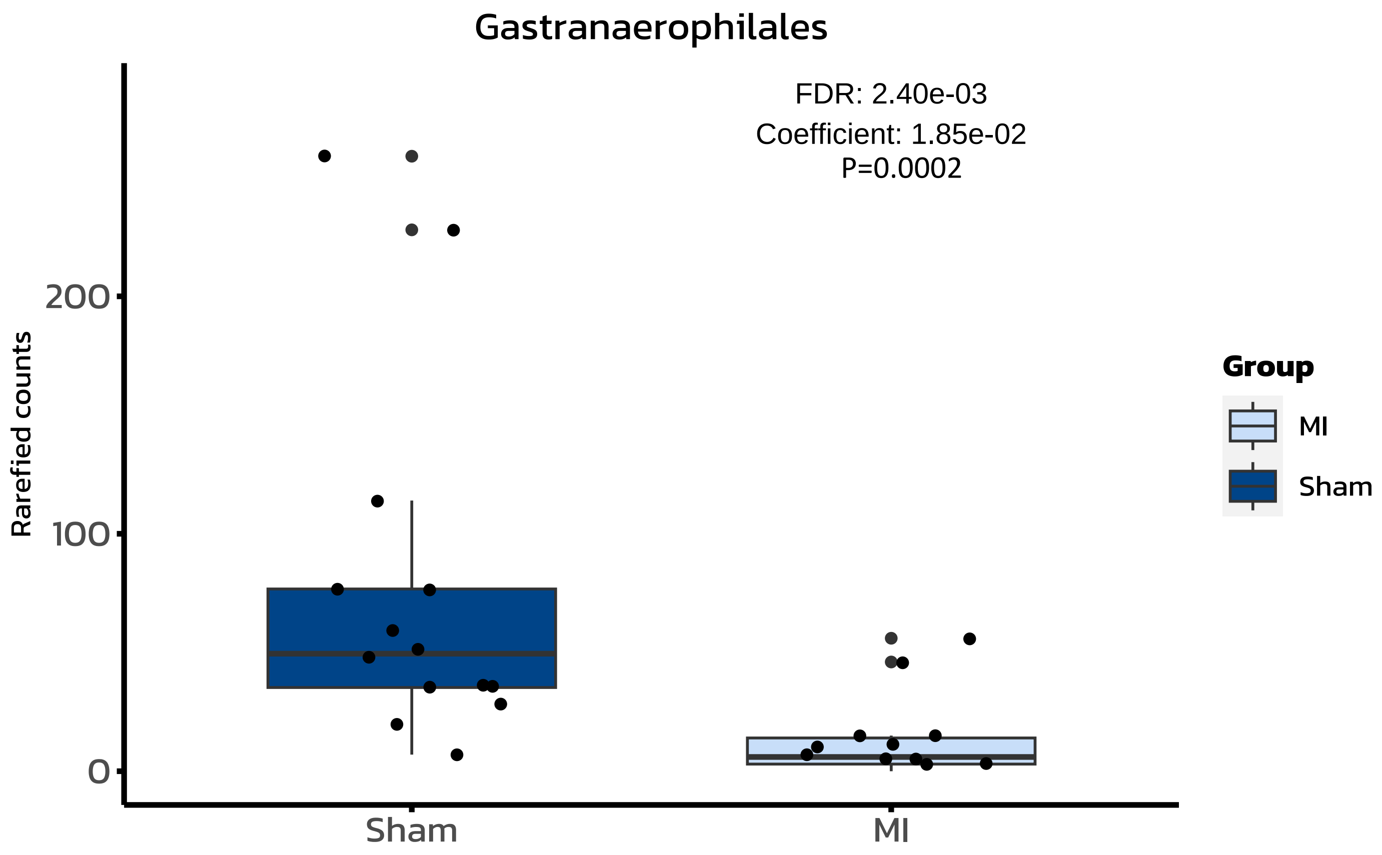
Gastranaerophilales
FDR: 2.40e-03
Coefficient: 1.85e-02
P=0.0002
Rarefied counts
200
100
0
Sham
MI
Group
MI
Sham

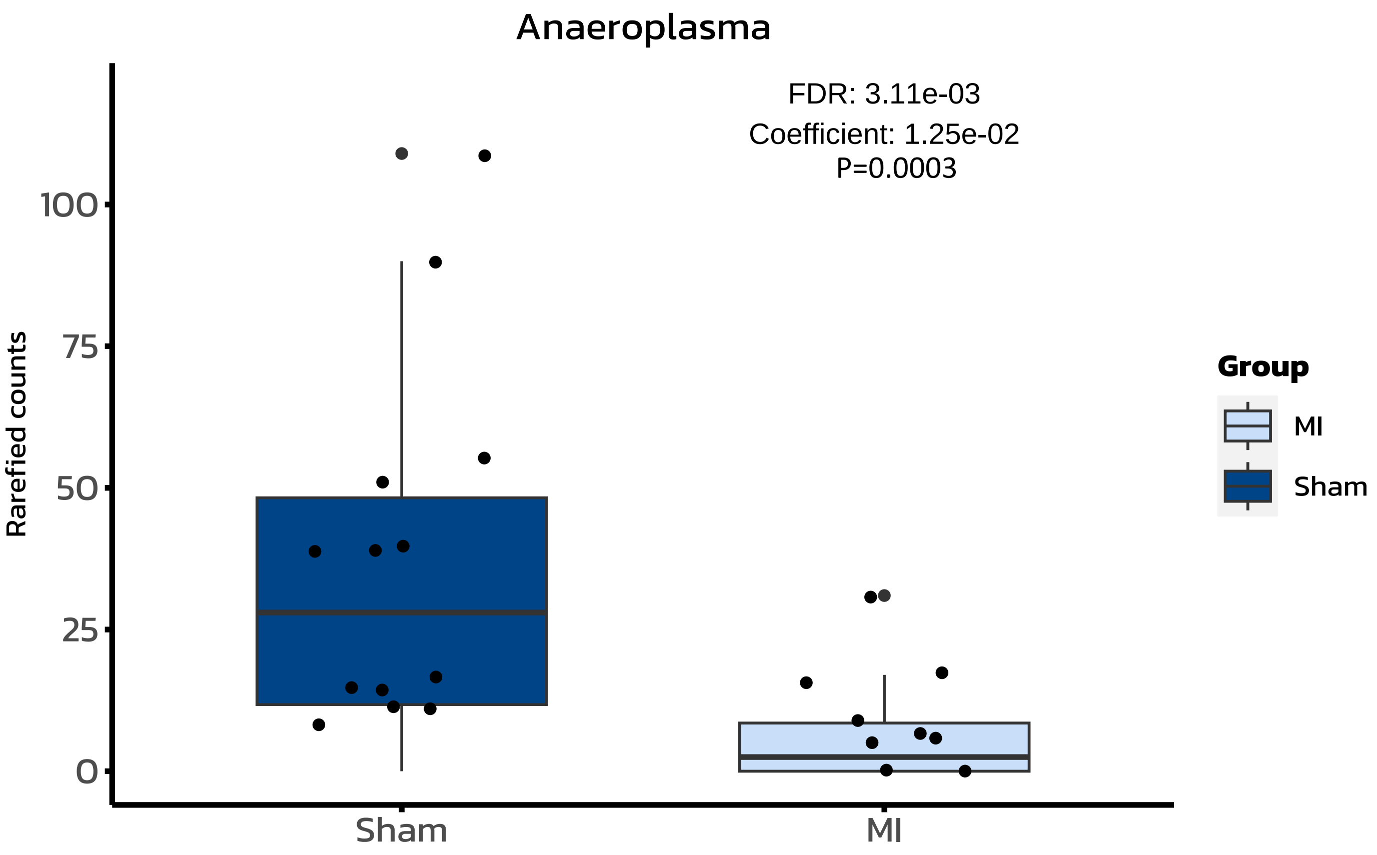

Anaeroplasma
FDR: 3.11e-03
Coefficient: 1.25e-02
P=0.0003
Rarefied counts
100
75
50
25
0
Sham
MI
Group
MI
Sham

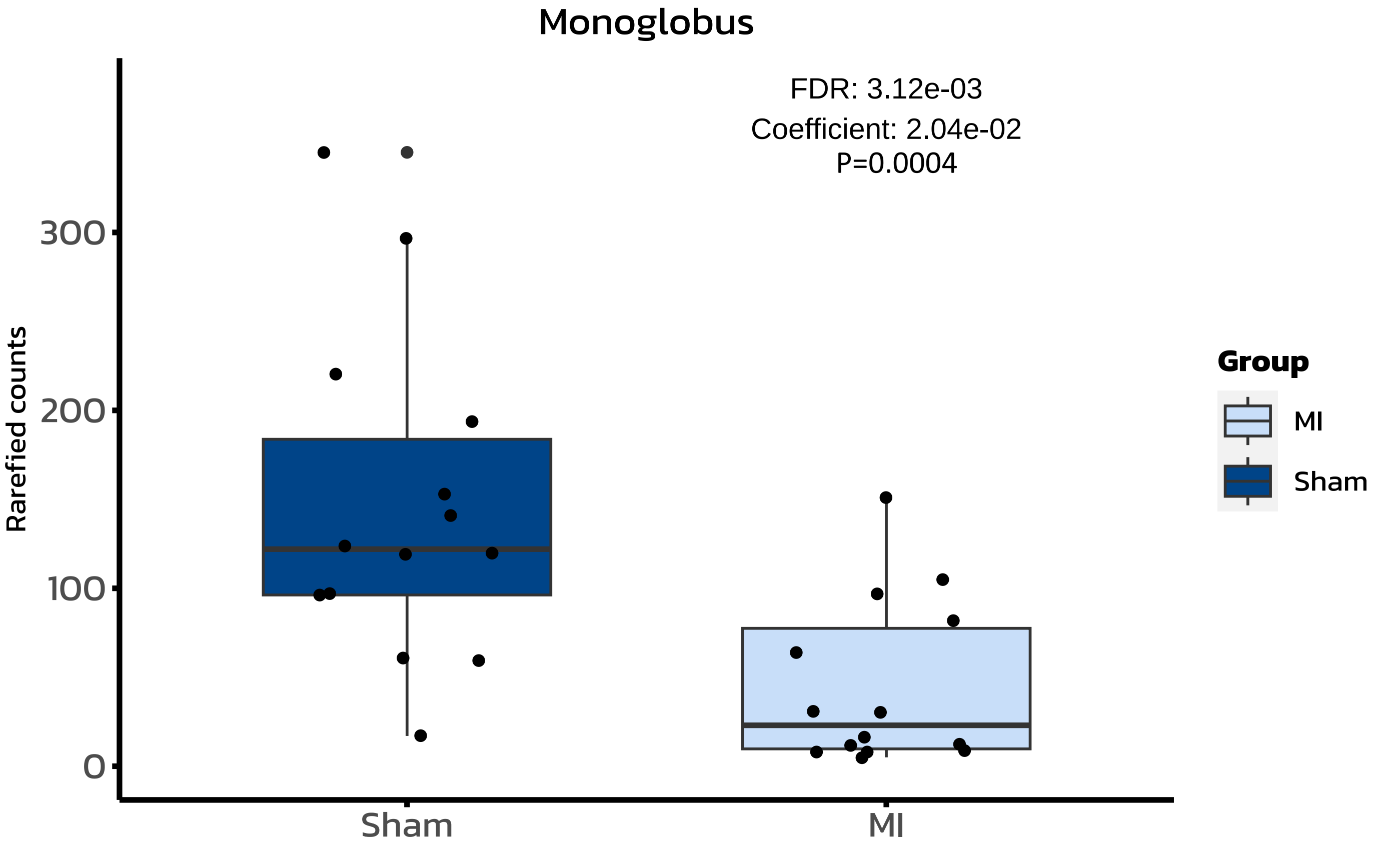

Monoglobus
FDR: 3.12e-03
Coefficient: 2.04e-02
P=0.0004
Rarefied counts
300
200
100
0
Sham
MI
Group
MI
Sham

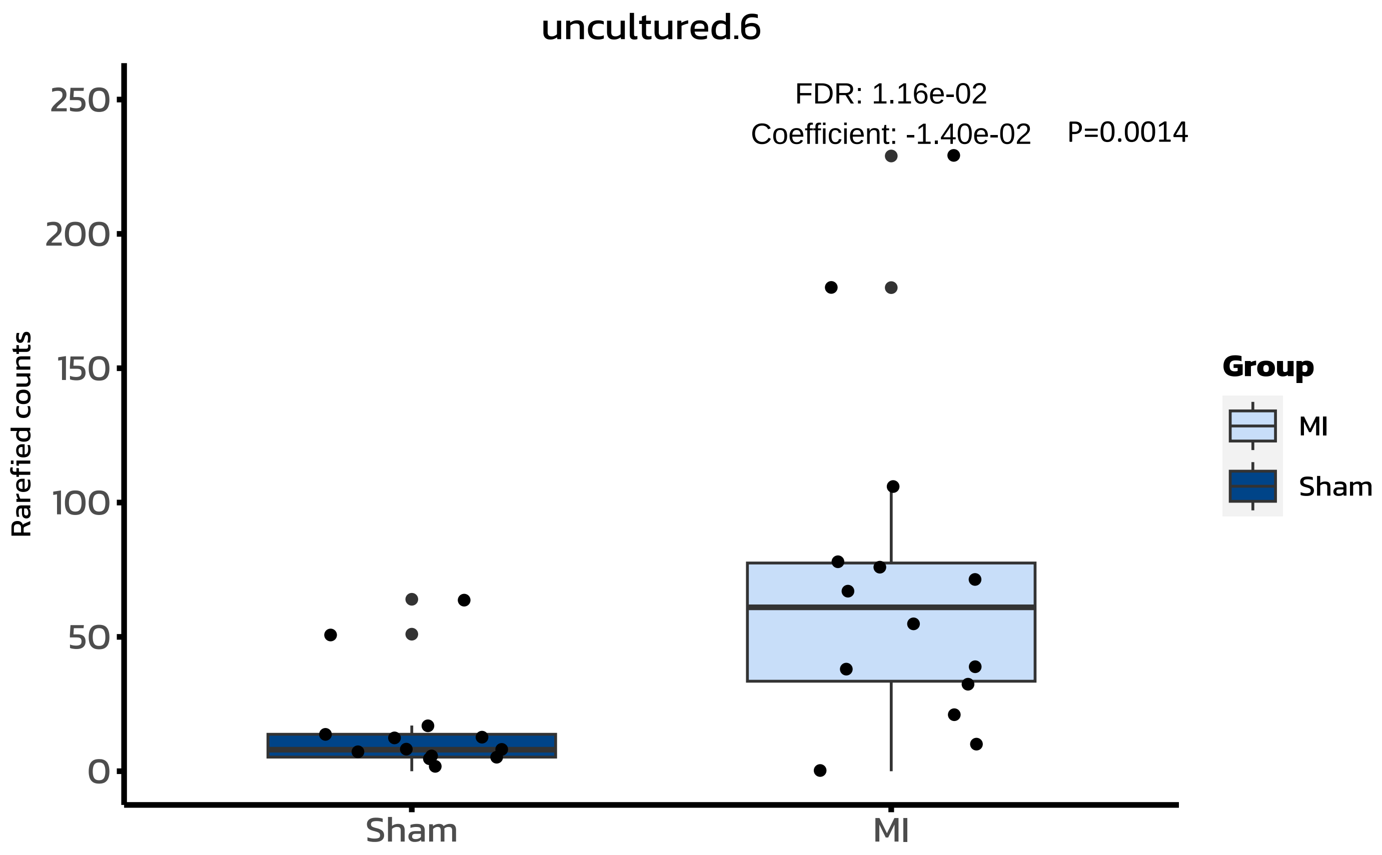
uncultured.6
FDR: 1.16e-02
Coefficient: -1.40e-02
P=0.0014
Rarefied counts
250
200
150
100
50
0
Sham
MI
Group
MI
Sham

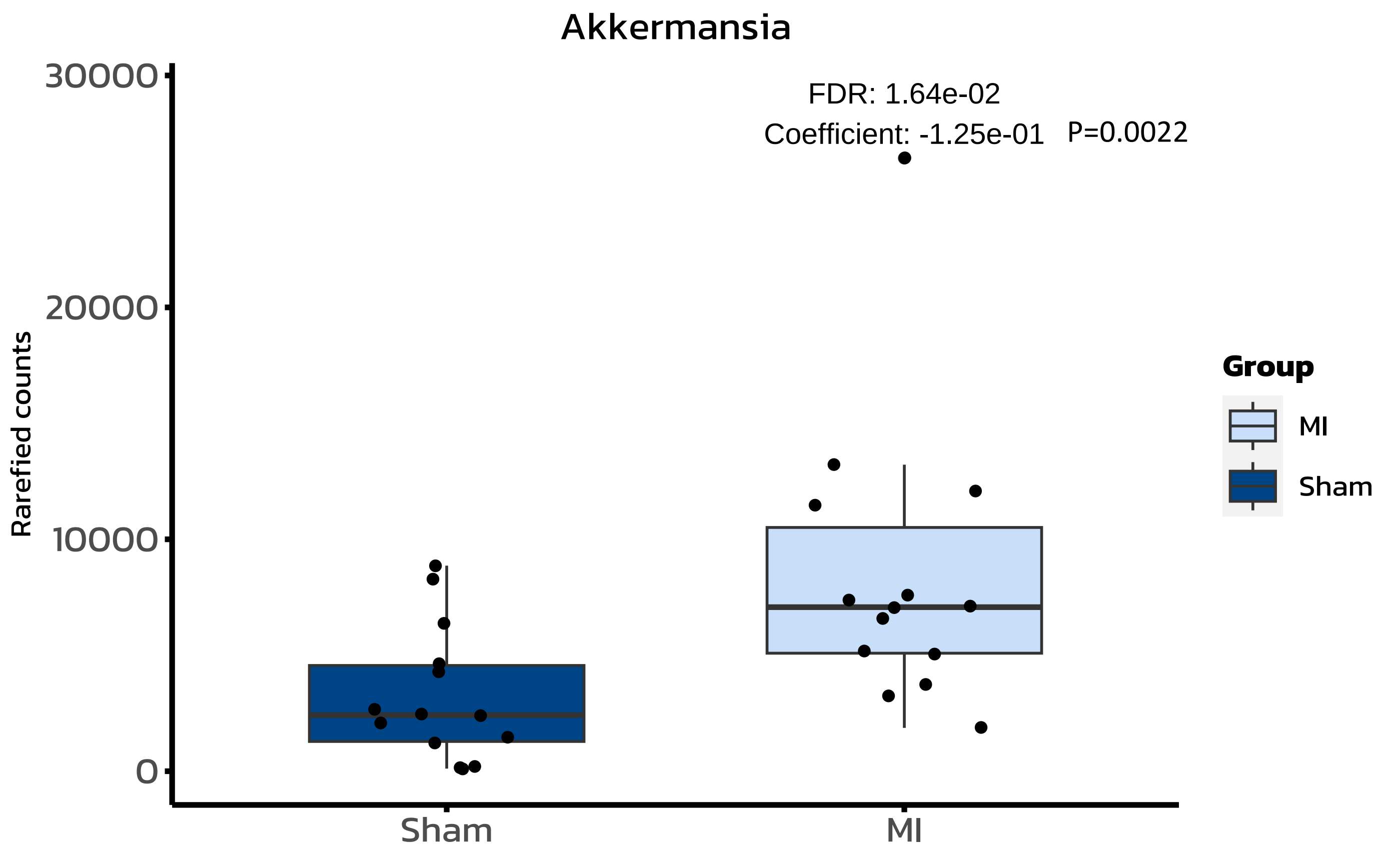
Akkermansia
FDR: 1.64e-02
Coefficient: -1.25e-01
P=0.0022
30000
20000
10000
0
Rarefied counts
Sham
MI
Group
MI
Sham

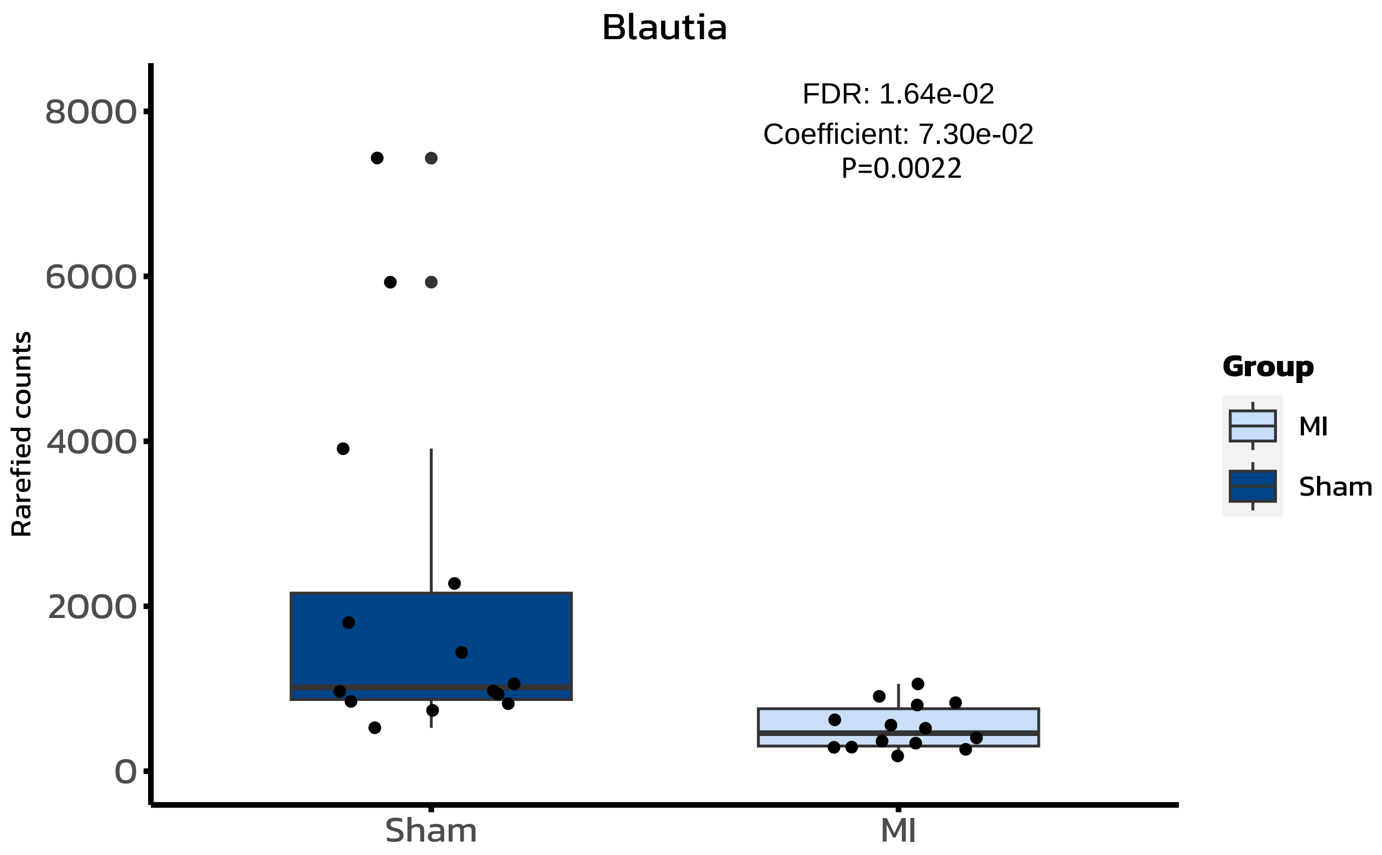
Blautia
FDR: 1.64e-02
Coefficient: 7.30e-02
P=0.0022
Rarefied counts
8000
6000
4000
2000
0
Sham
MI
Group
MI
Sham

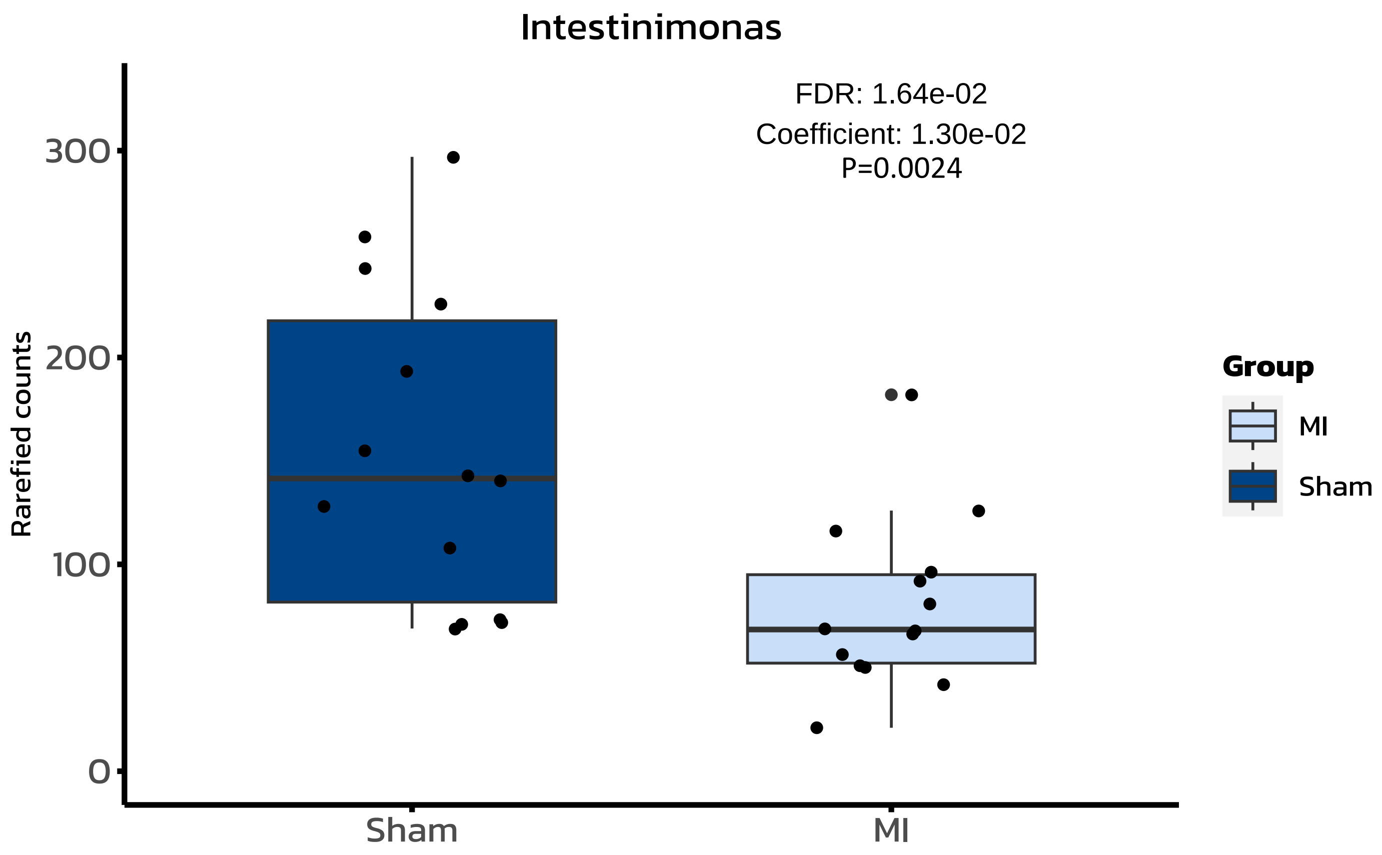

Intestinimonas
FDR: 1.64e-02
Coefficient: 1.30e-02
P=0.0024
Rarefied counts
300
200
100
0
Sham
MI
Group
MI
Sham

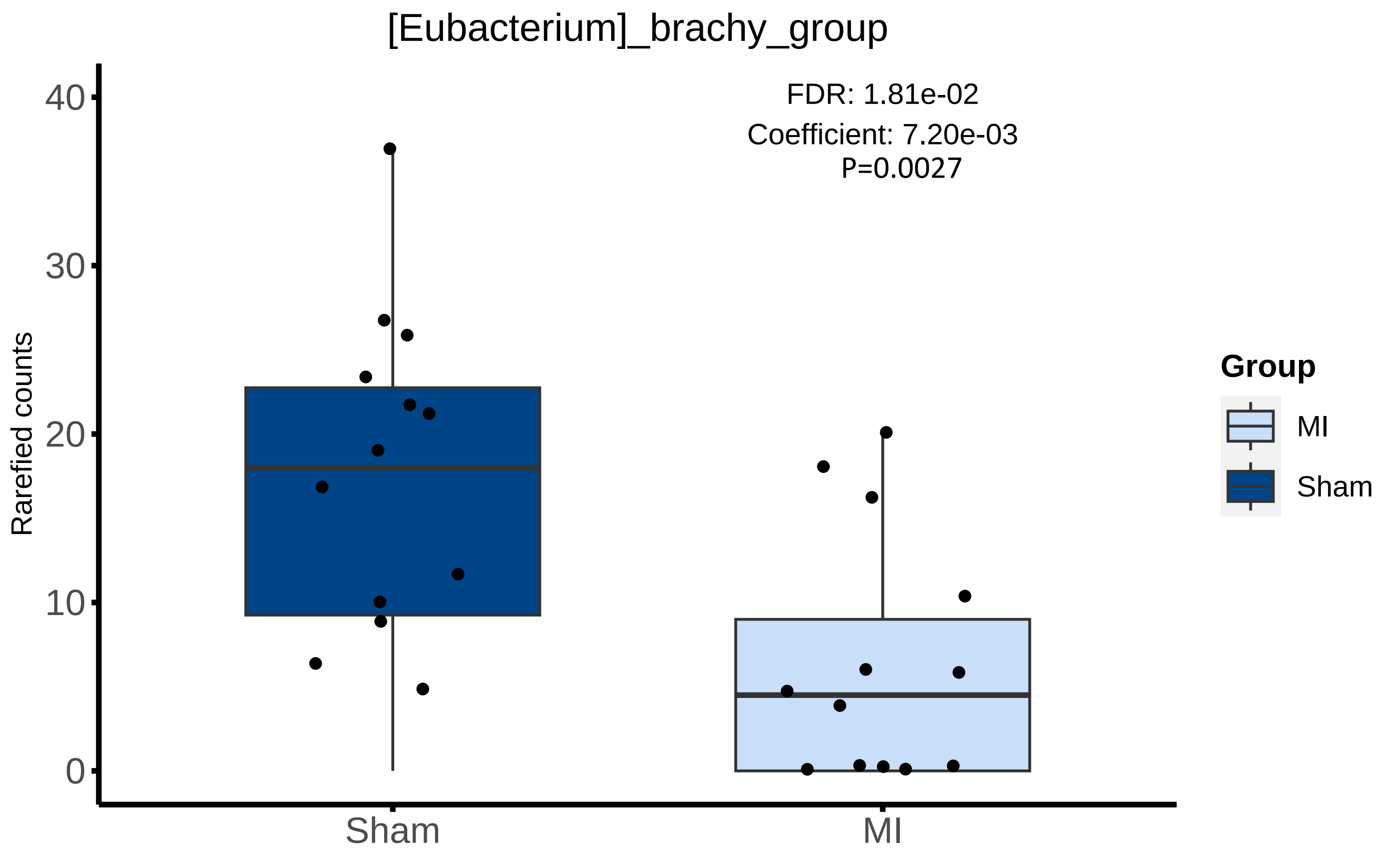

[Eubacterium]_brachy_group
FDR: 1.81e-02
Coefficient: 7.20e-03
P=0.0027
Rarefied counts
40
30
20
10
0
Sham
MI
Group
MI
Sham

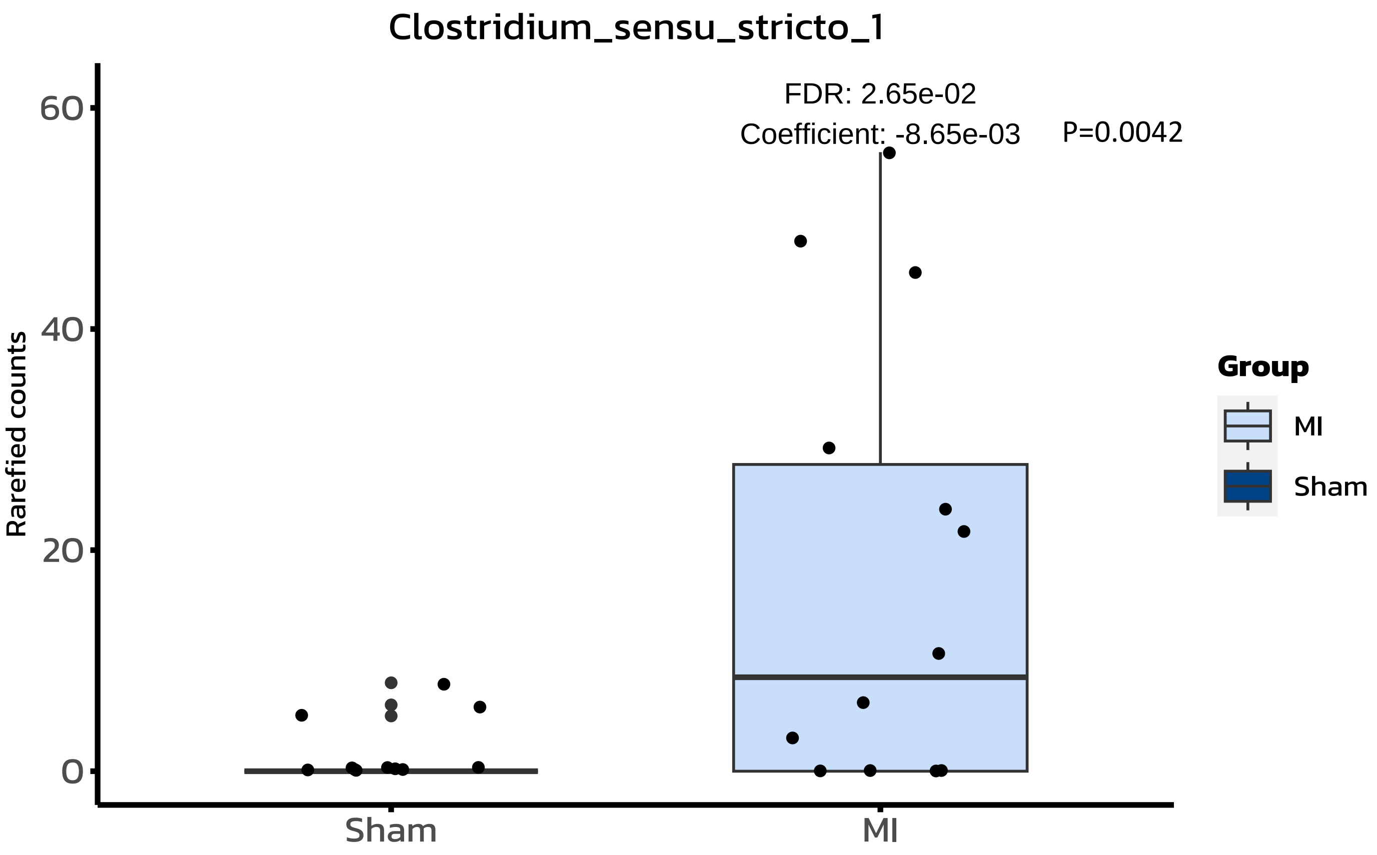
Clostridium_sensu_stricto_1
FDR: 2.65e-02
Coefficient: -8.65e-03
P=0.0042
Rarefied counts
60
40
20
0
Sham
MI
Group
MI
Sham

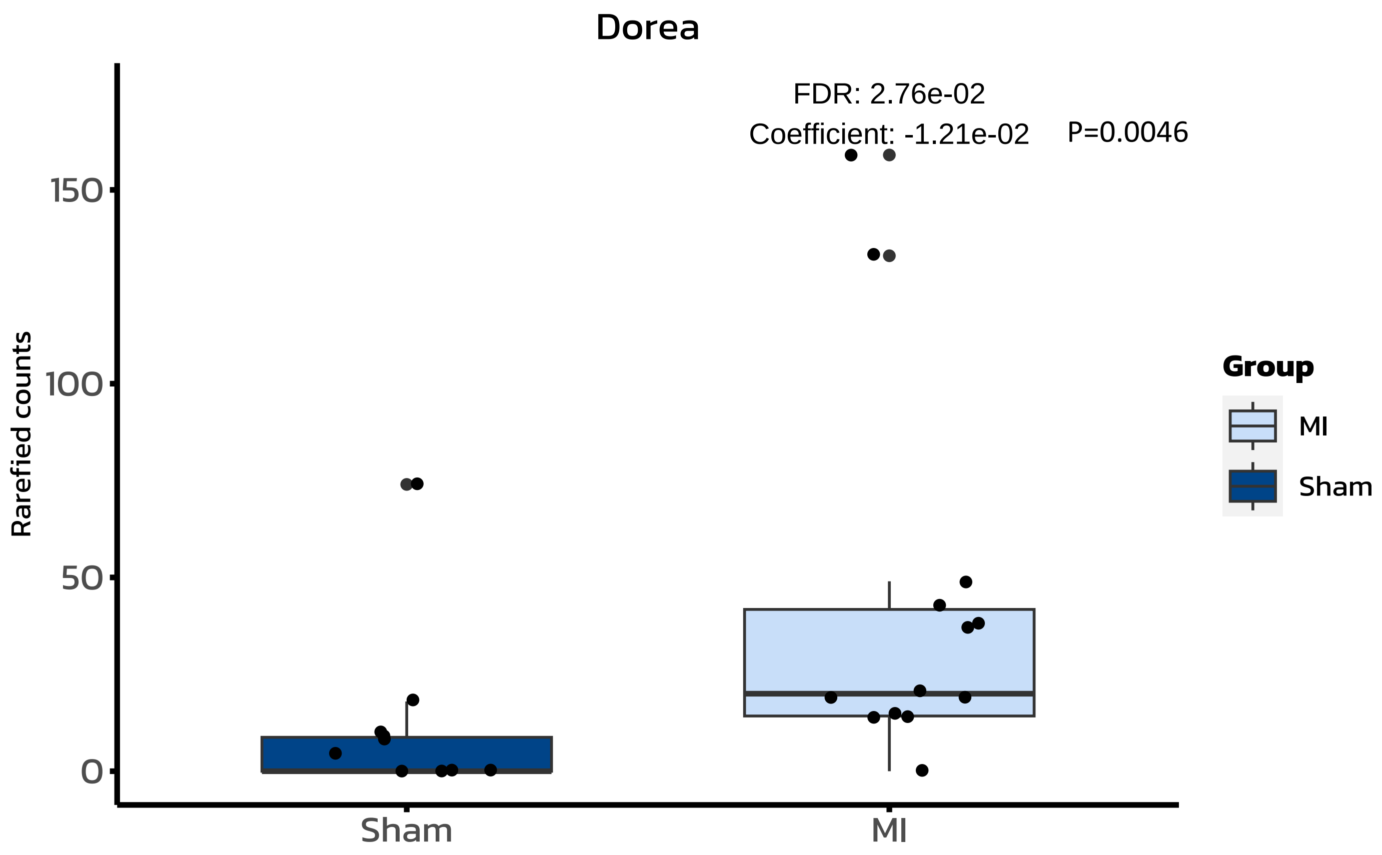
Dorea
FDR: 2.76e-02
Coefficient: -1.21e-02
P=0.0046
Rarefied counts
150
100
50
0
Sham
MI
Group
MI
Sham

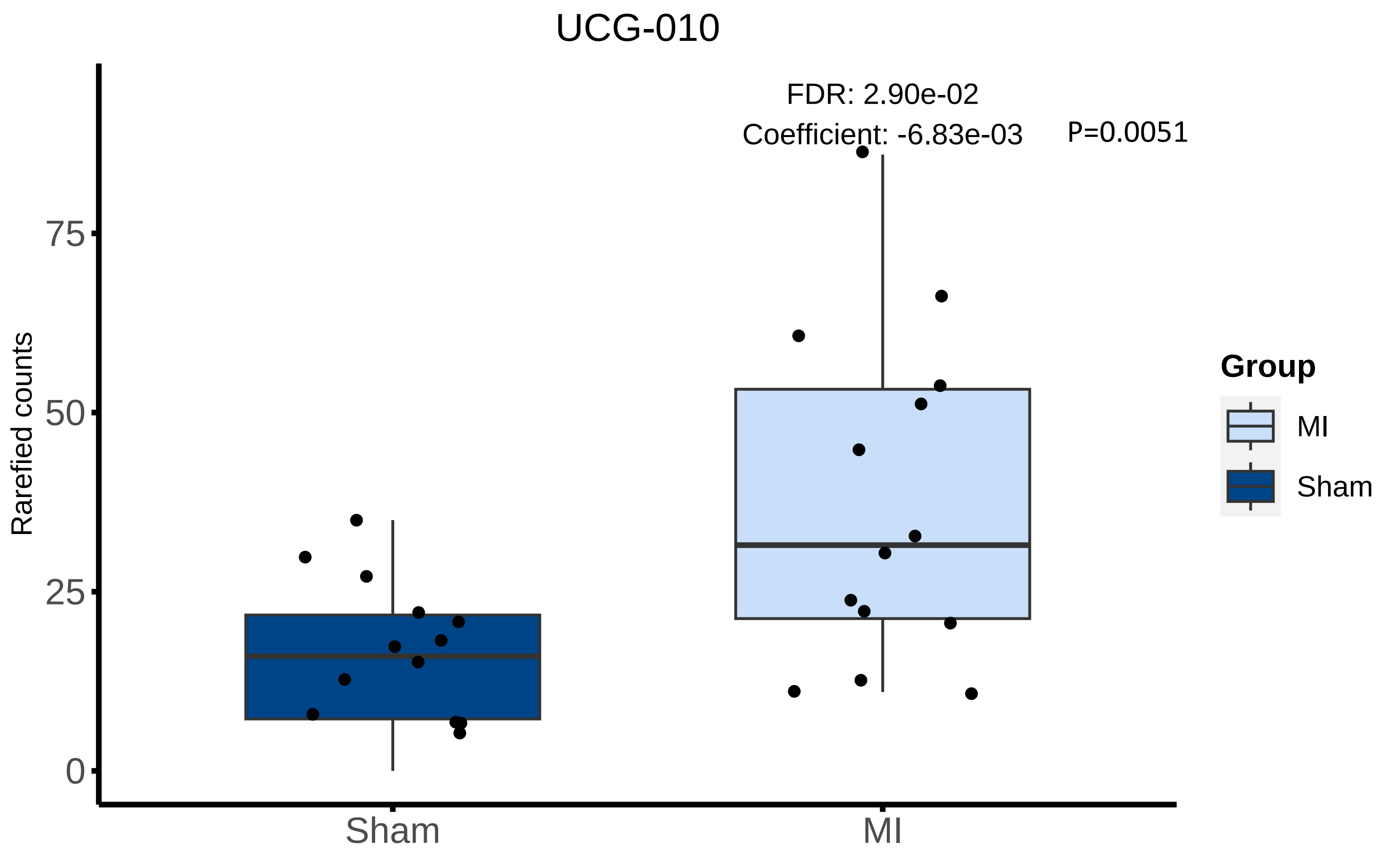
UCG-010
FDR: 2.90e-02
Coefficient: -6.83e-03
P=0.0051
Rarefied counts
75
50
25
0
Sham
MI
Group
MI
Sham

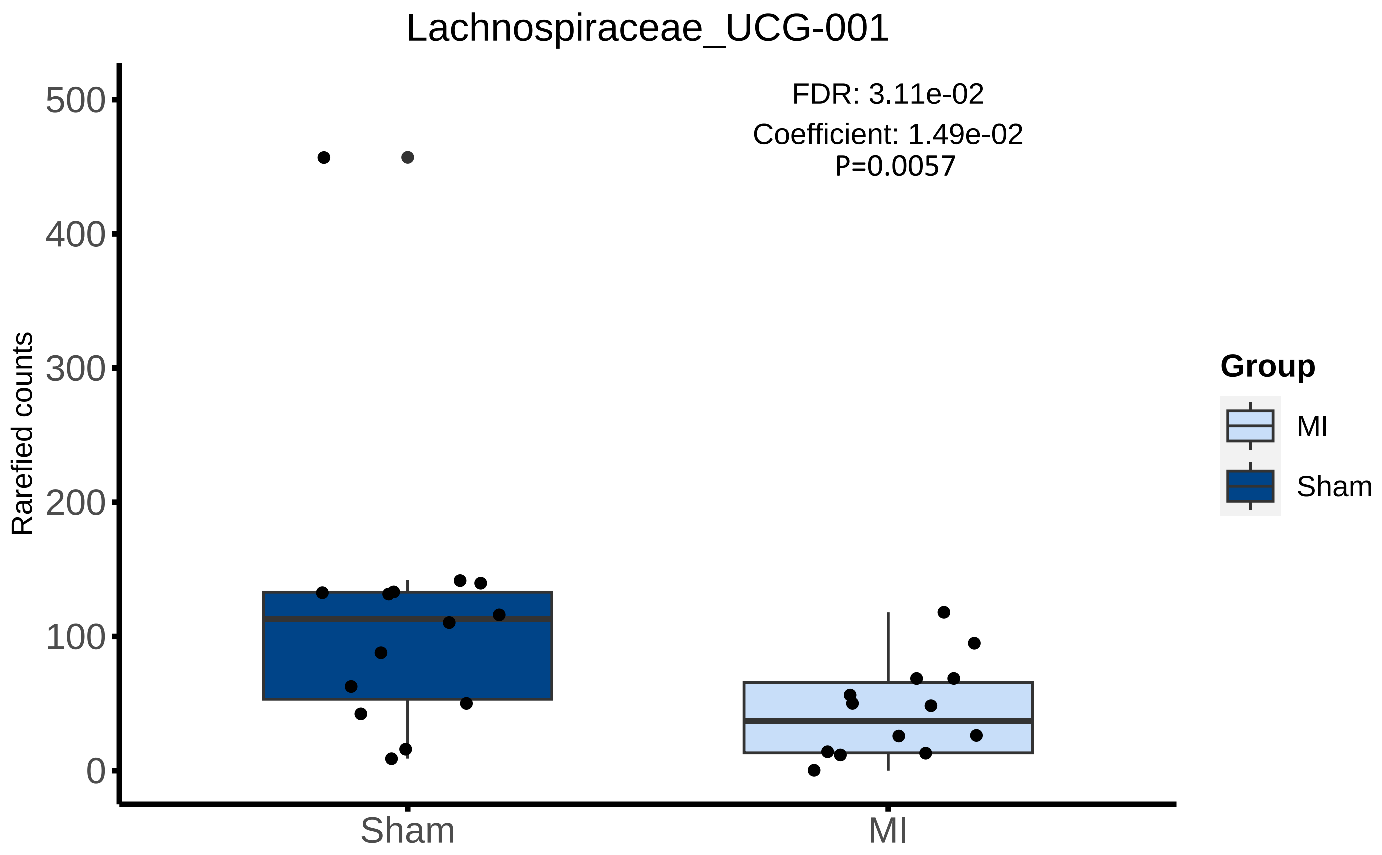
Lachnospiraceae_UCG-001
FDR: 3.11e-02
Coefficient: 1.49e-02
P=0.0057
Rarefied counts
500
400
300
200
100
0
Sham
MI
Group
MI
Sham

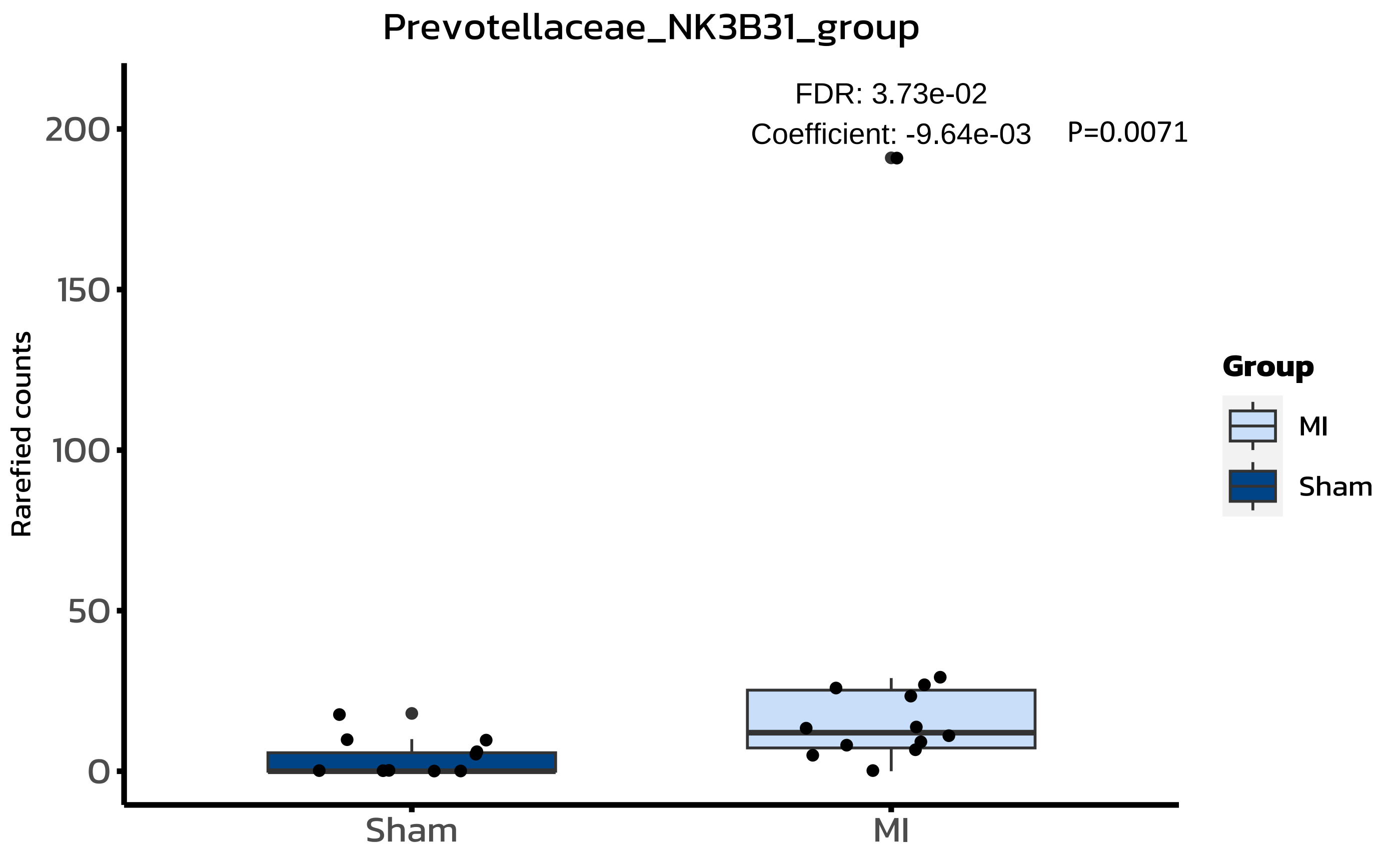
Prevotellaceae_NK3B31_group
FDR: 3.73e-02
Coefficient: -9.64e-03
P=0.0071
Rarefied counts
200
150
100
50
0
Sham
MI
Group
MI
Sham

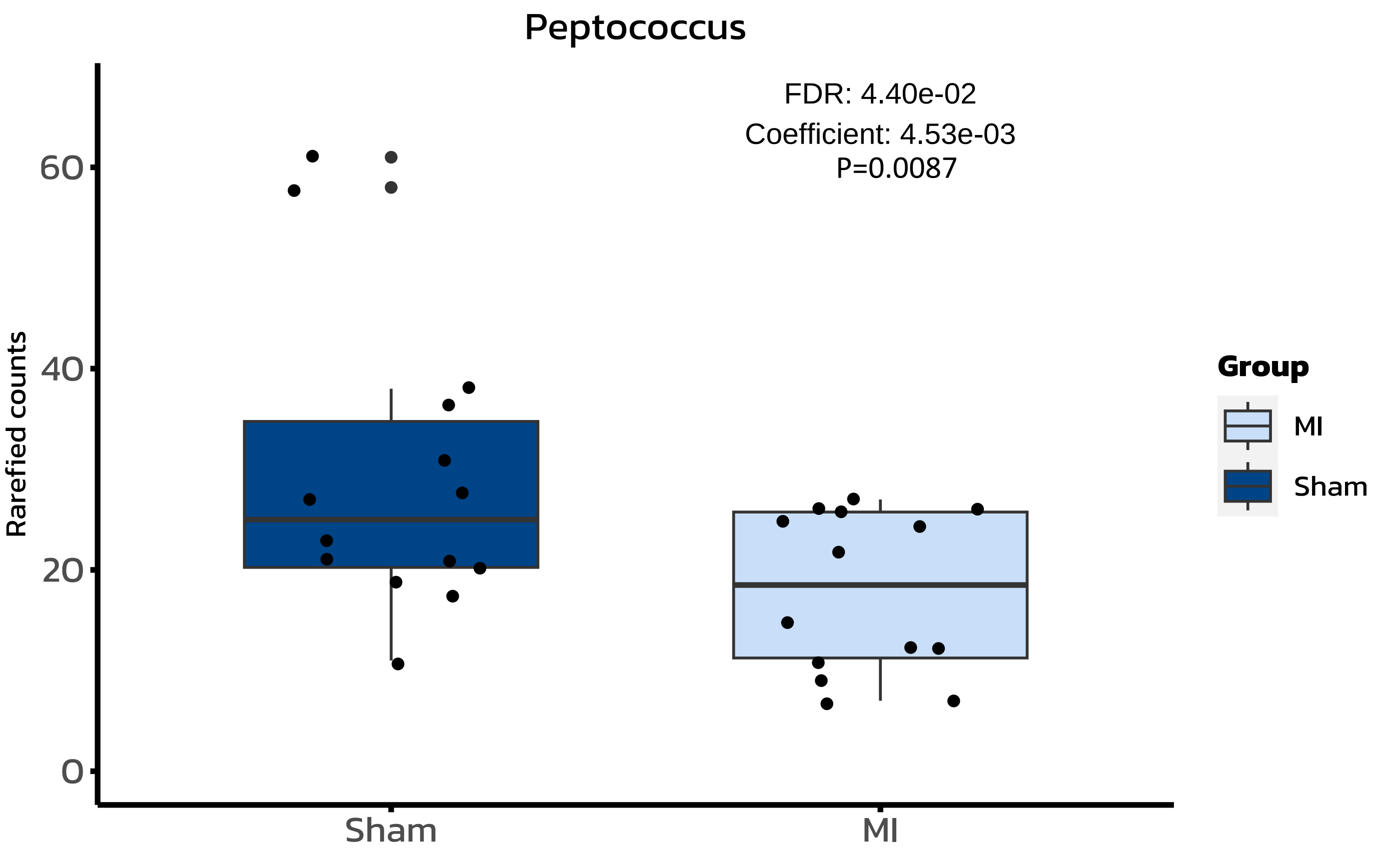

Peptococcus
FDR: 4.40e-02
Coefficient: 4.53e-03
P=0.0087
Rarefied counts
60
40
20
0
Sham
MI
Group
MI
Sham

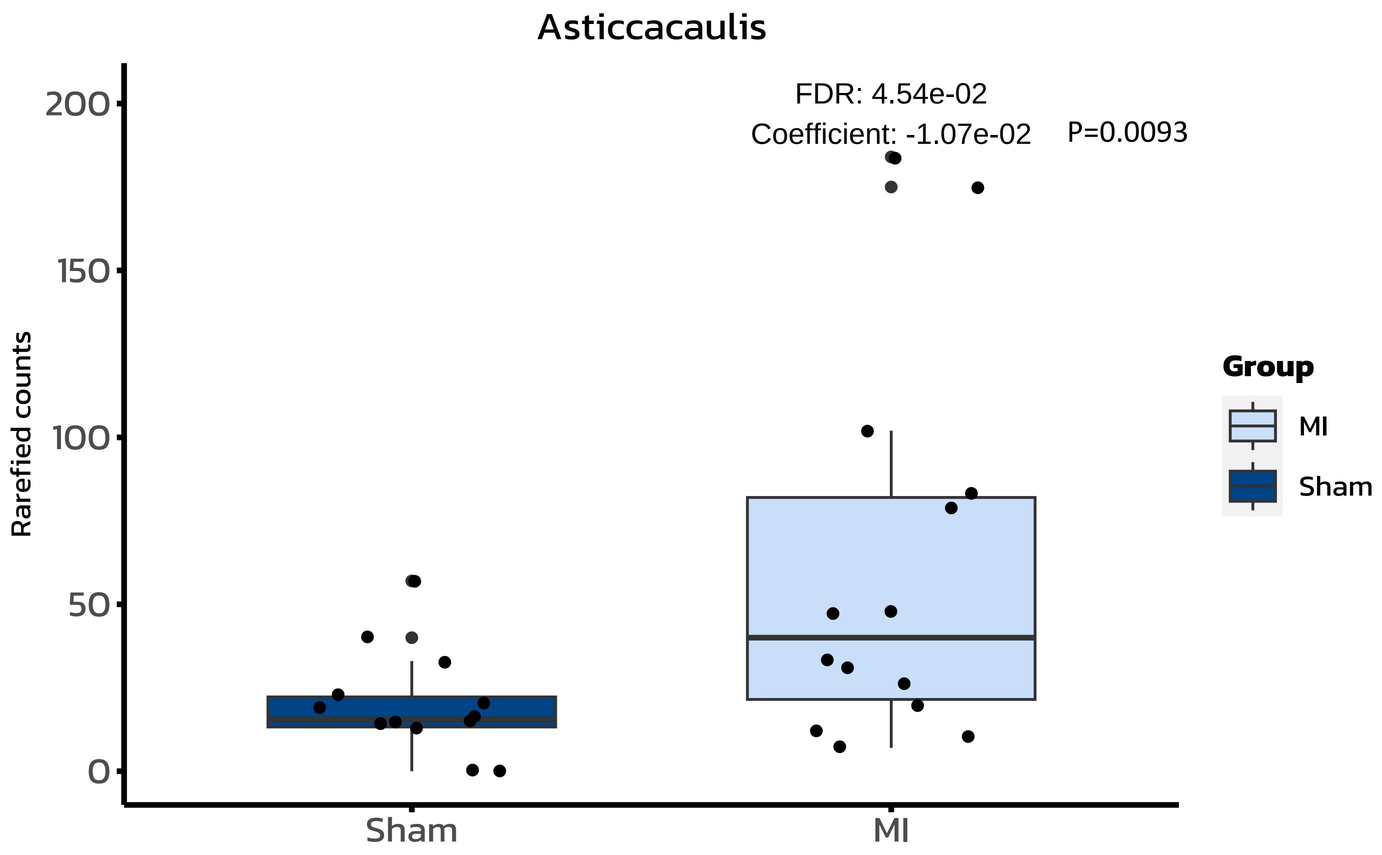

Asticcacaulis
FDR: 4.54e-02
Coefficient: -1.07e-02
P=0.0093
Rarefied counts
200
150
100
50
0
Sham
MI
Group
MI
Sham

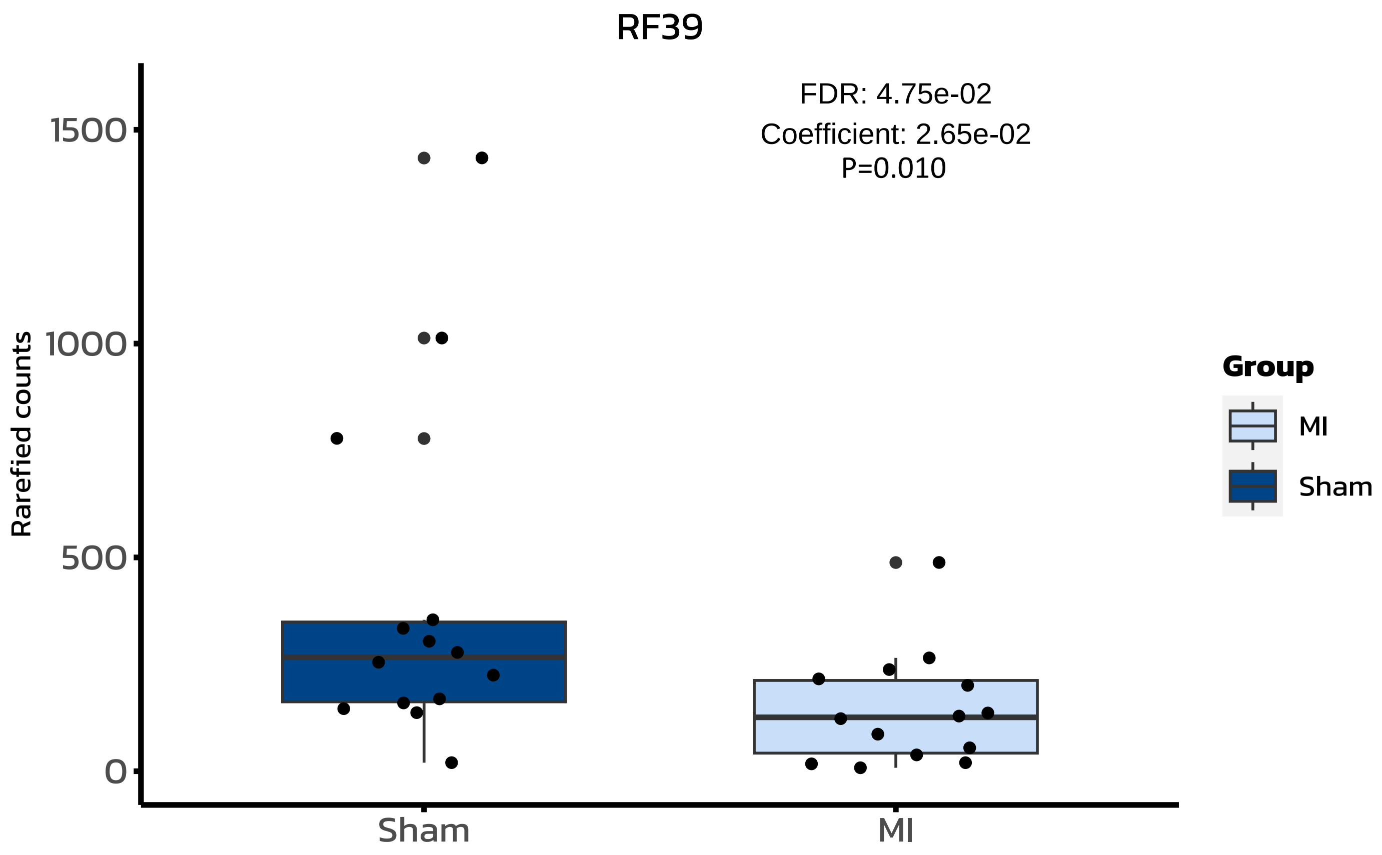
RF39
FDR: 4.75e-02
Coefficient: 2.65e-02
P=0.010
Rarefied counts
1500
1000
500
0
Sham
MI
Group
MI
Sham

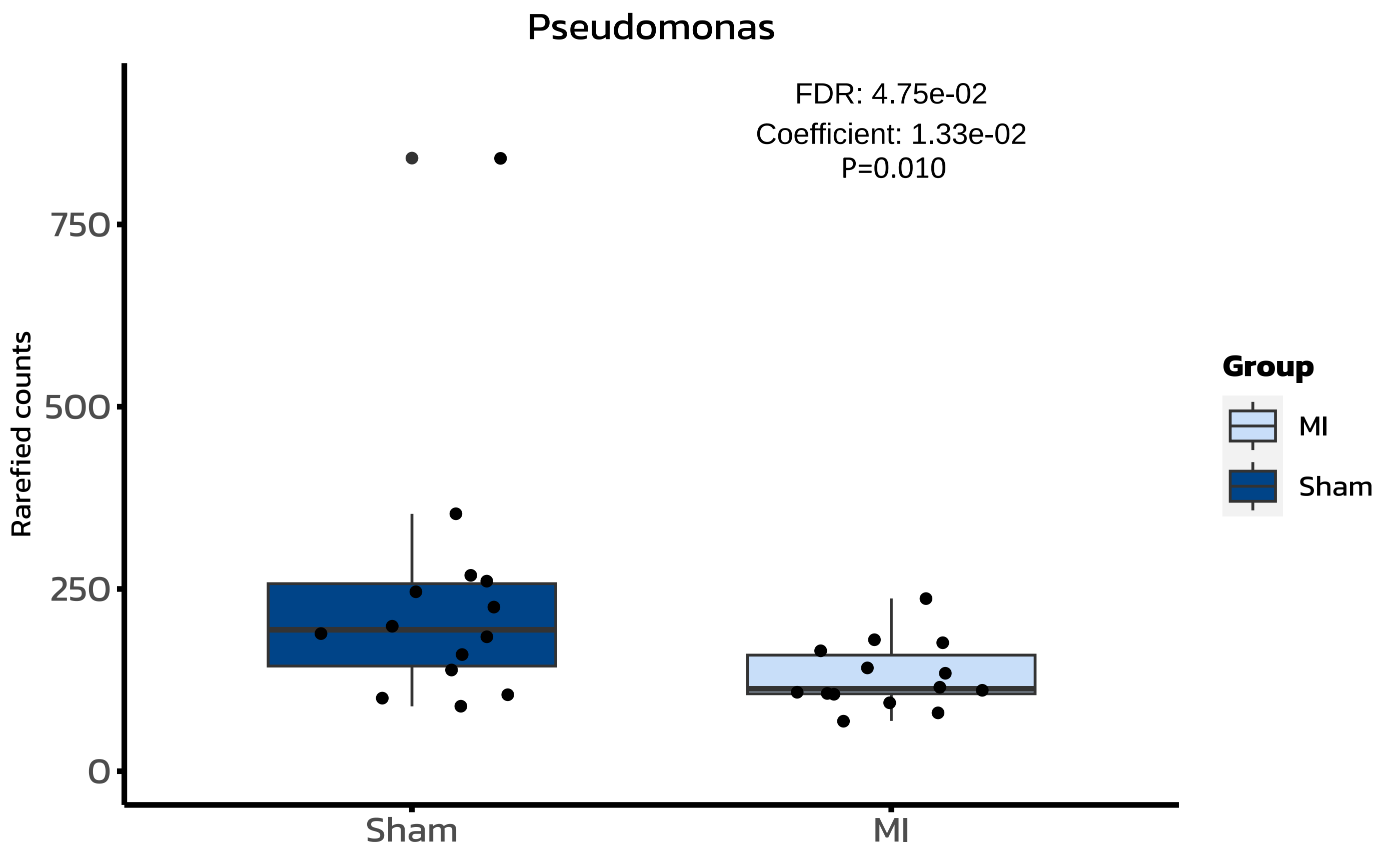
Pseudomonas
FDR: 4.75e-02
Coefficient: 1.33e-02
P=0.010
Rarefied counts
0
250
500
750
Sham
MI
Group
MI
Sham

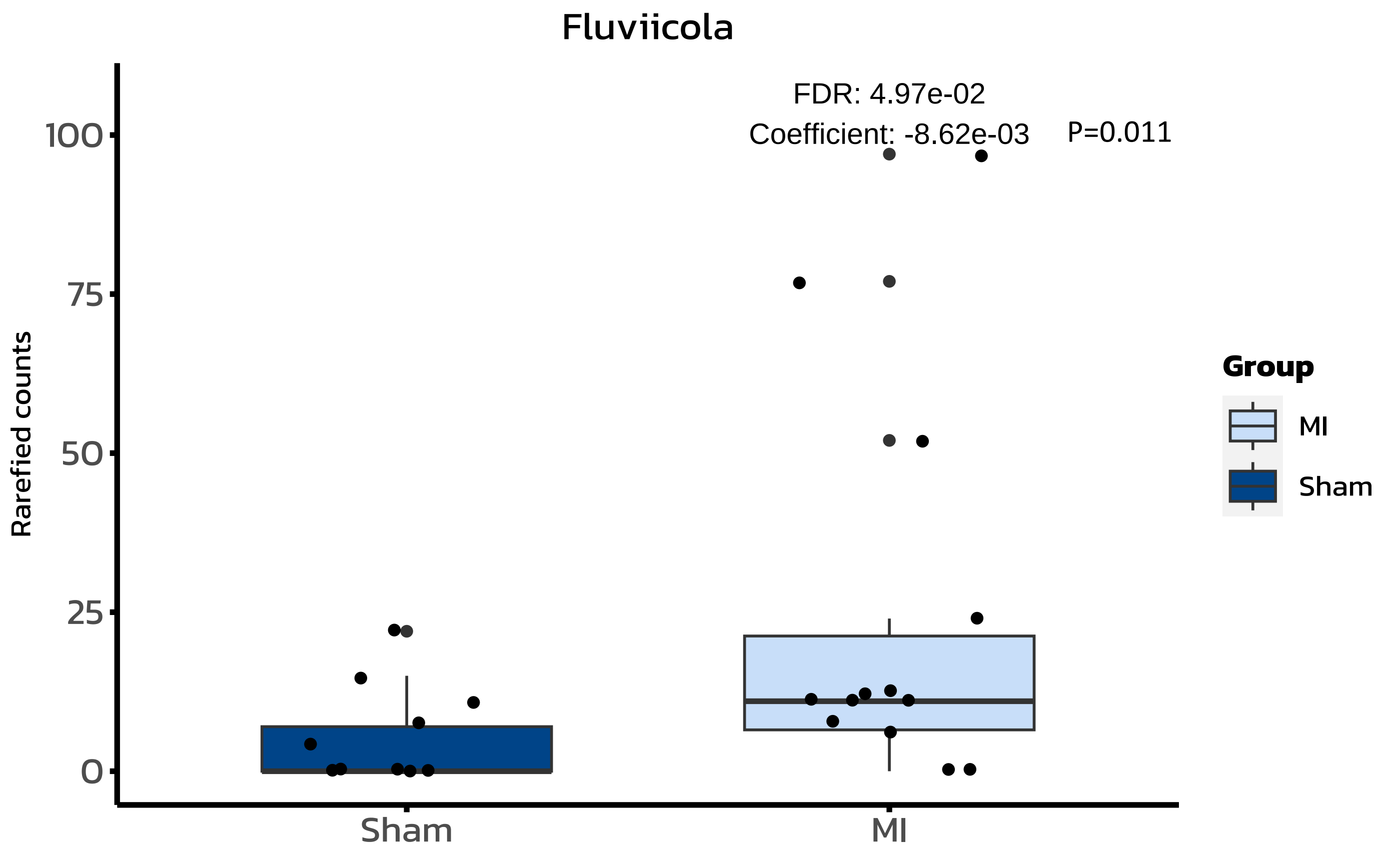
Fluviicola
FDR: 4.97e-02
Coefficient: -8.62e-03
P=0.011
Rarefied counts
100
75
50
25
0
Sham
MI
Group
MI
Sham

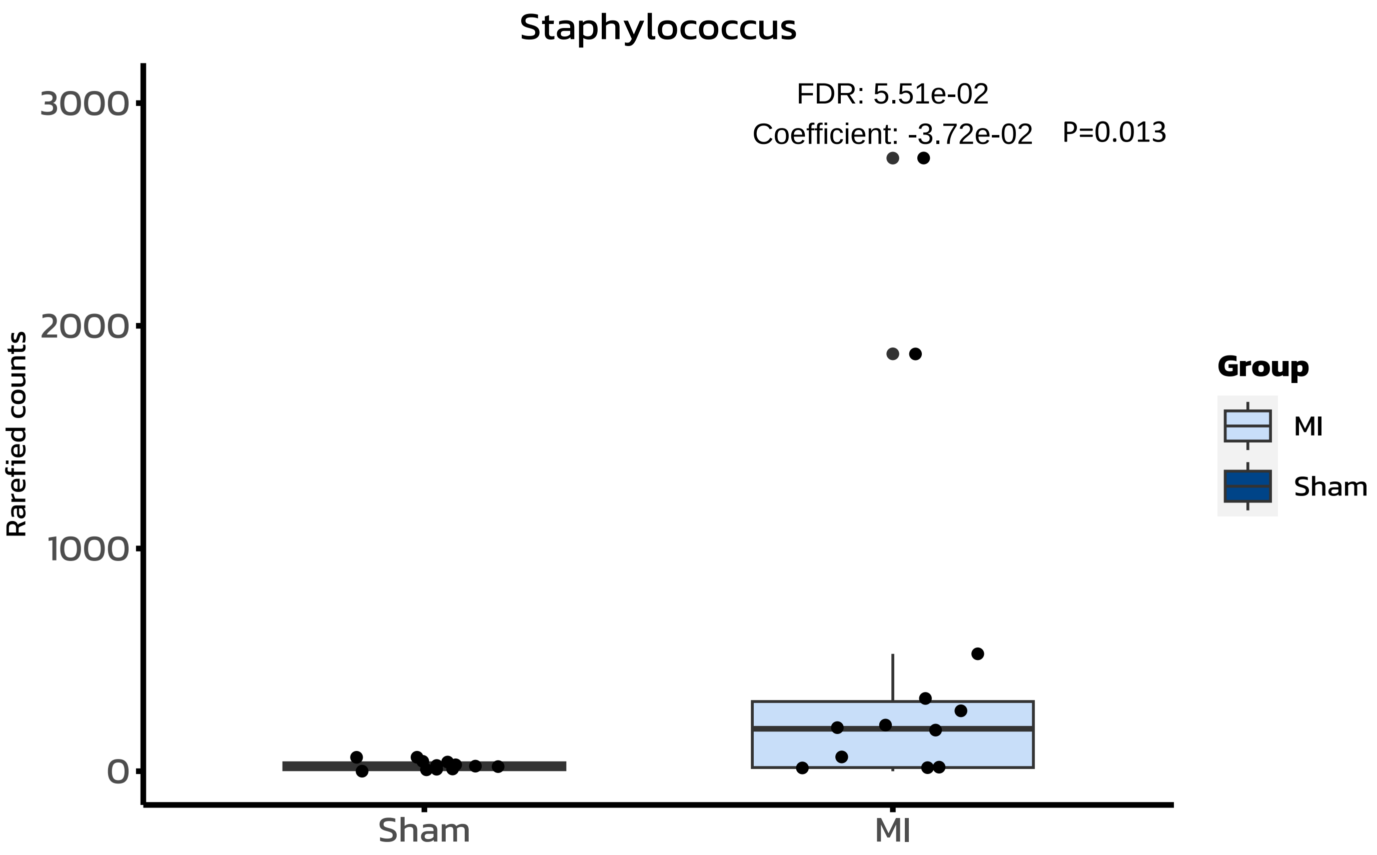
Staphylococcus
FDR: 5.51e-02
Coefficient: -3.72e-02
P=0.013
3000
2000
1000
0
Rarefied counts
Sham
MI
Group
MI
Sham

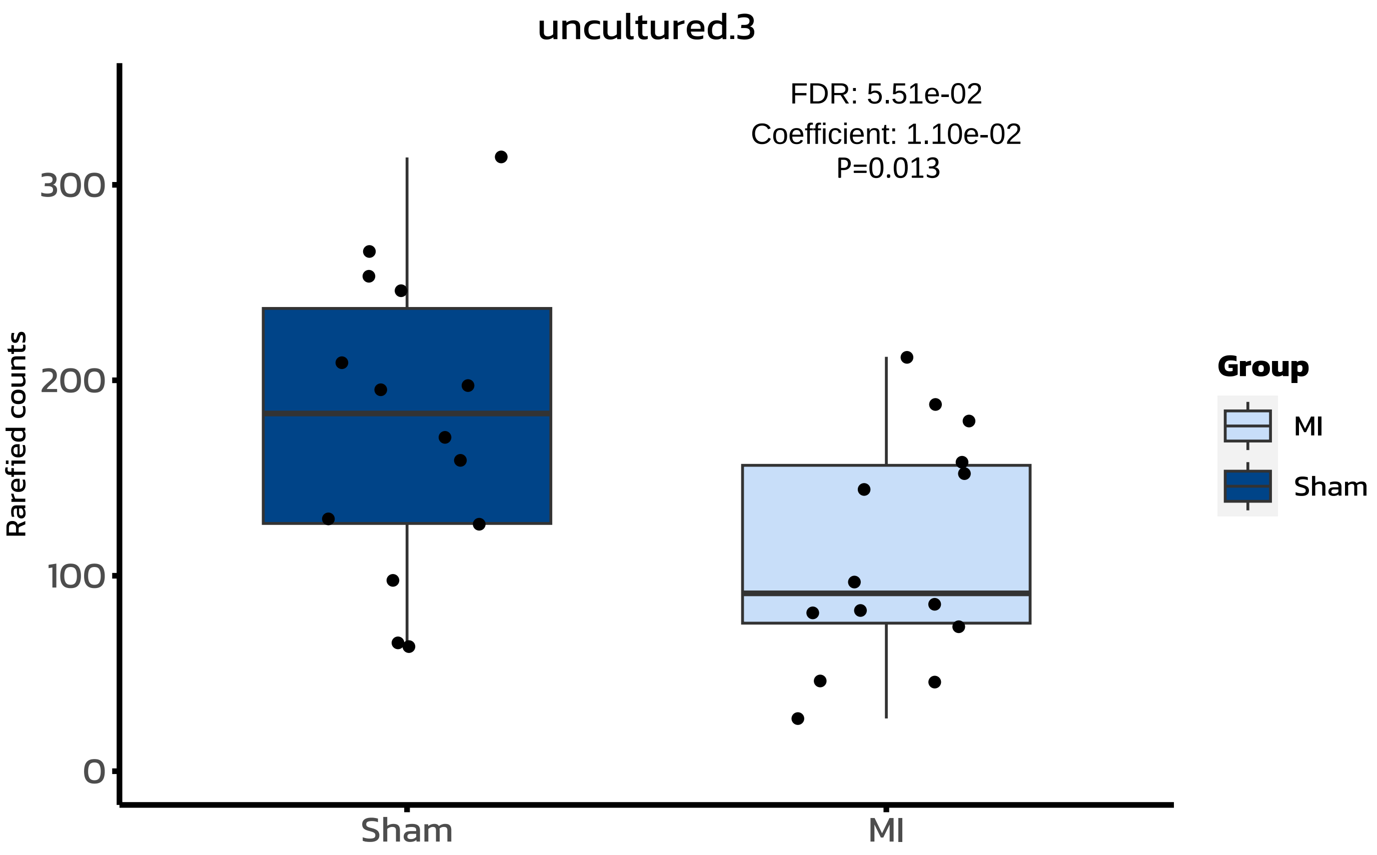
uncultured.3
FDR: 5.51e-02
Coefficient: 1.10e-02
P=0.013
Rarefied counts
300
200
100
0
Sham
MI
Group
MI
Sham

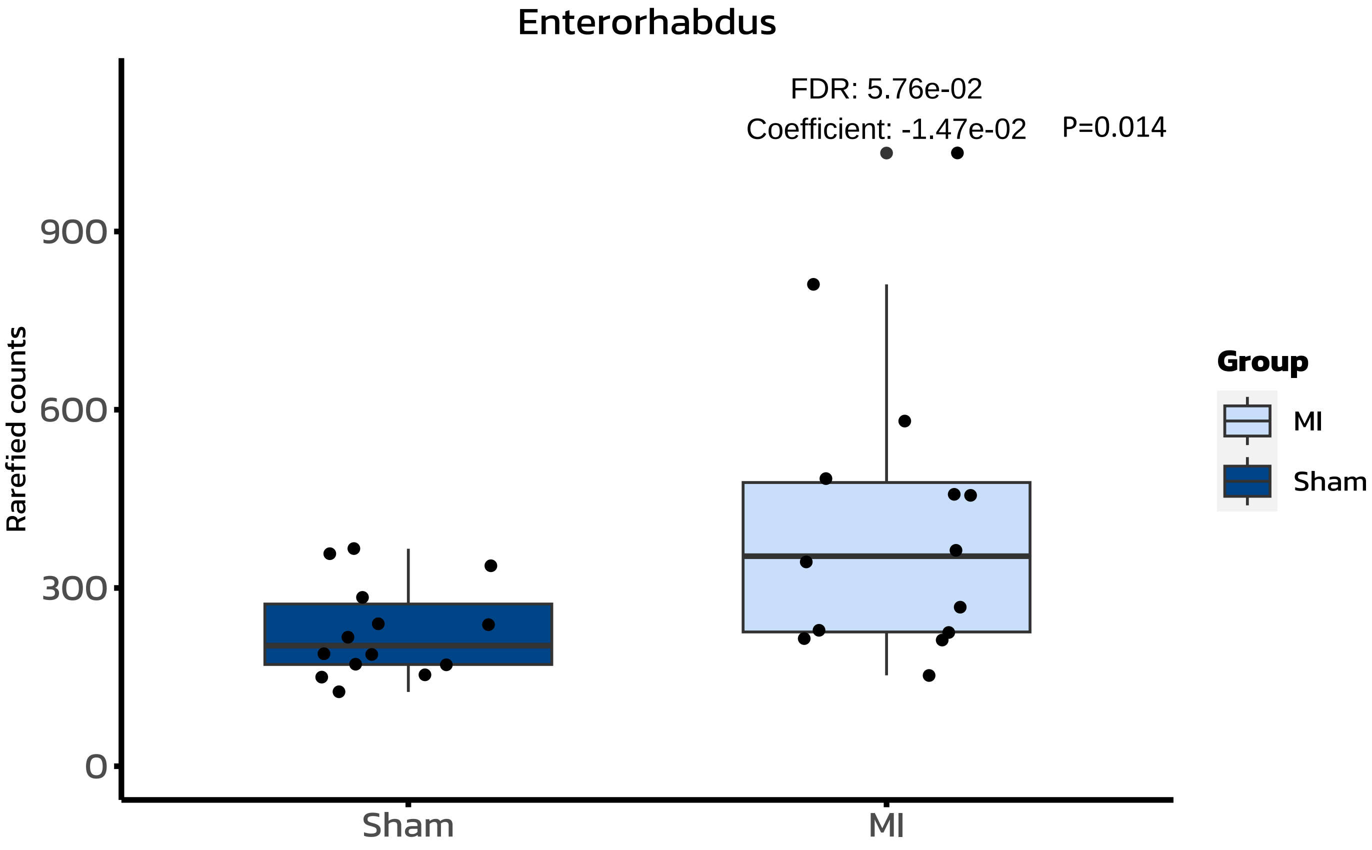
Enterorhabdus
FDR: 5.76e-02
Coefficient: -1.47e-02
P=0.014
900
600
300
0
Rarefied counts
Sham
MI
Group
MI
Sham

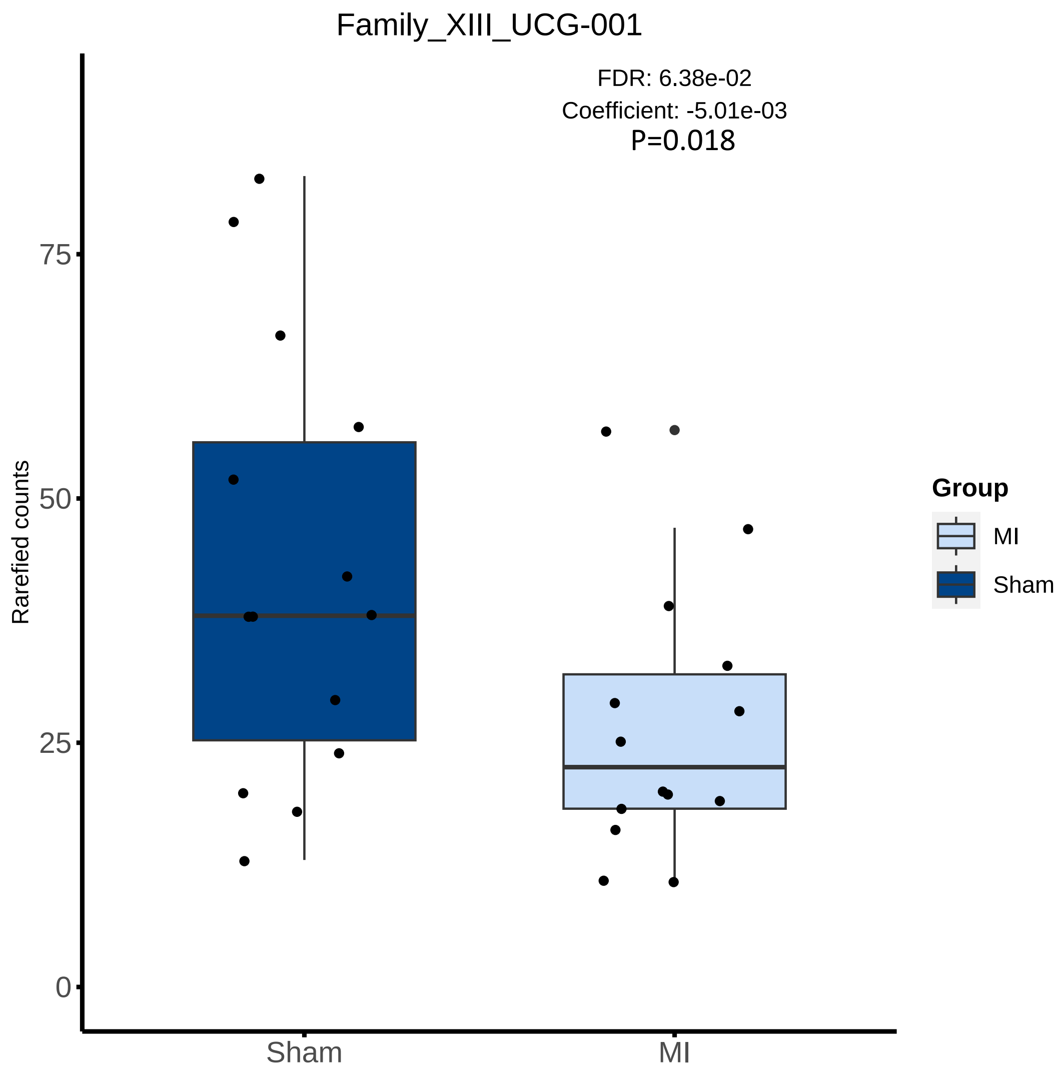

Family_XIII_UCG-001
FDR: 6.38e-02
Coefficient: -5.01e-03
P=0.018
Rarefied counts
75
50
25
0
Sham
MI
Group
MI
Sham

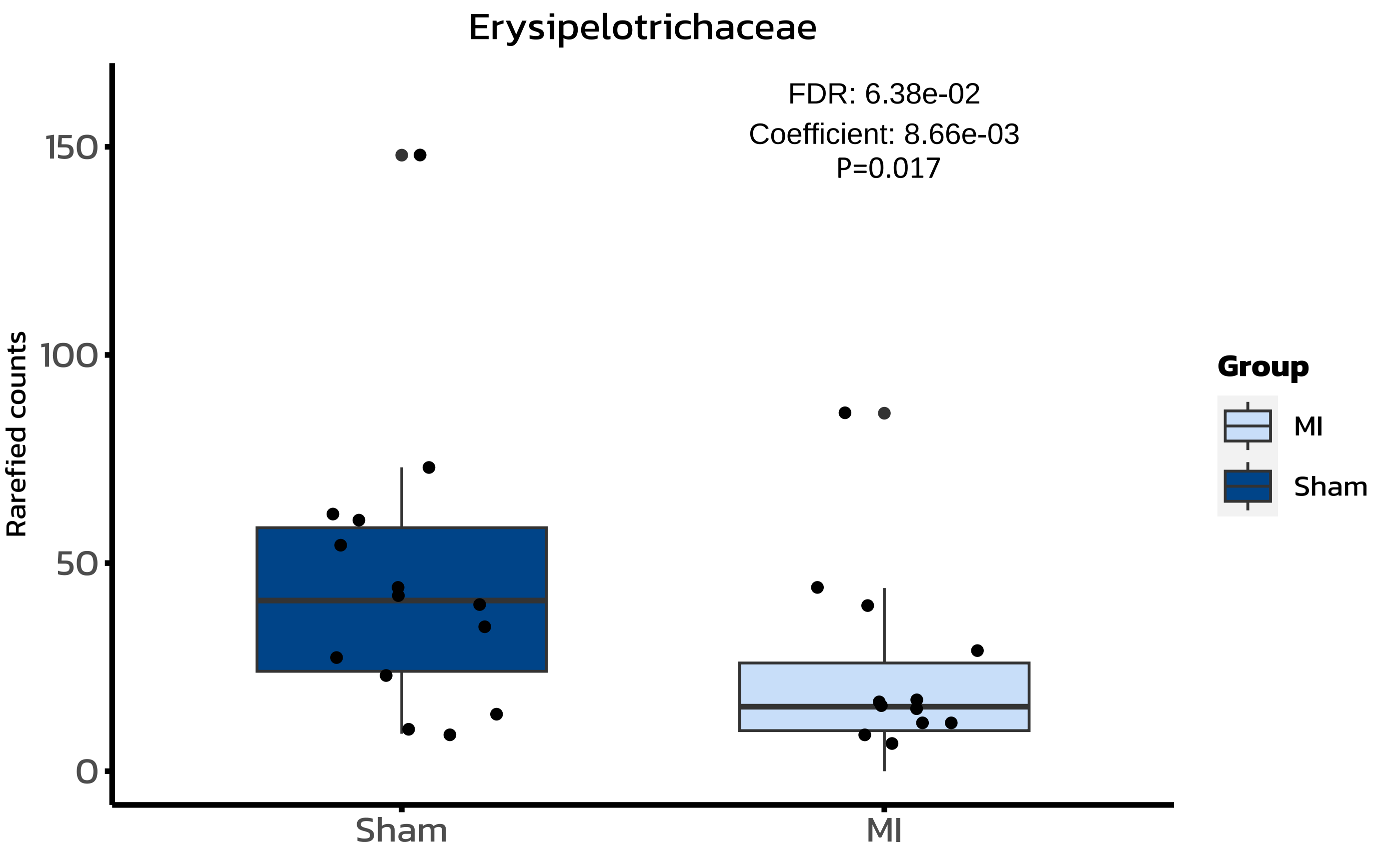

Erysipelotrichaceae
FDR: 6.38e-02
Coefficient: 8.66e-03
P=0.017
Rarefied counts
150
100
50
0
Sham
MI
Group
MI
Sham

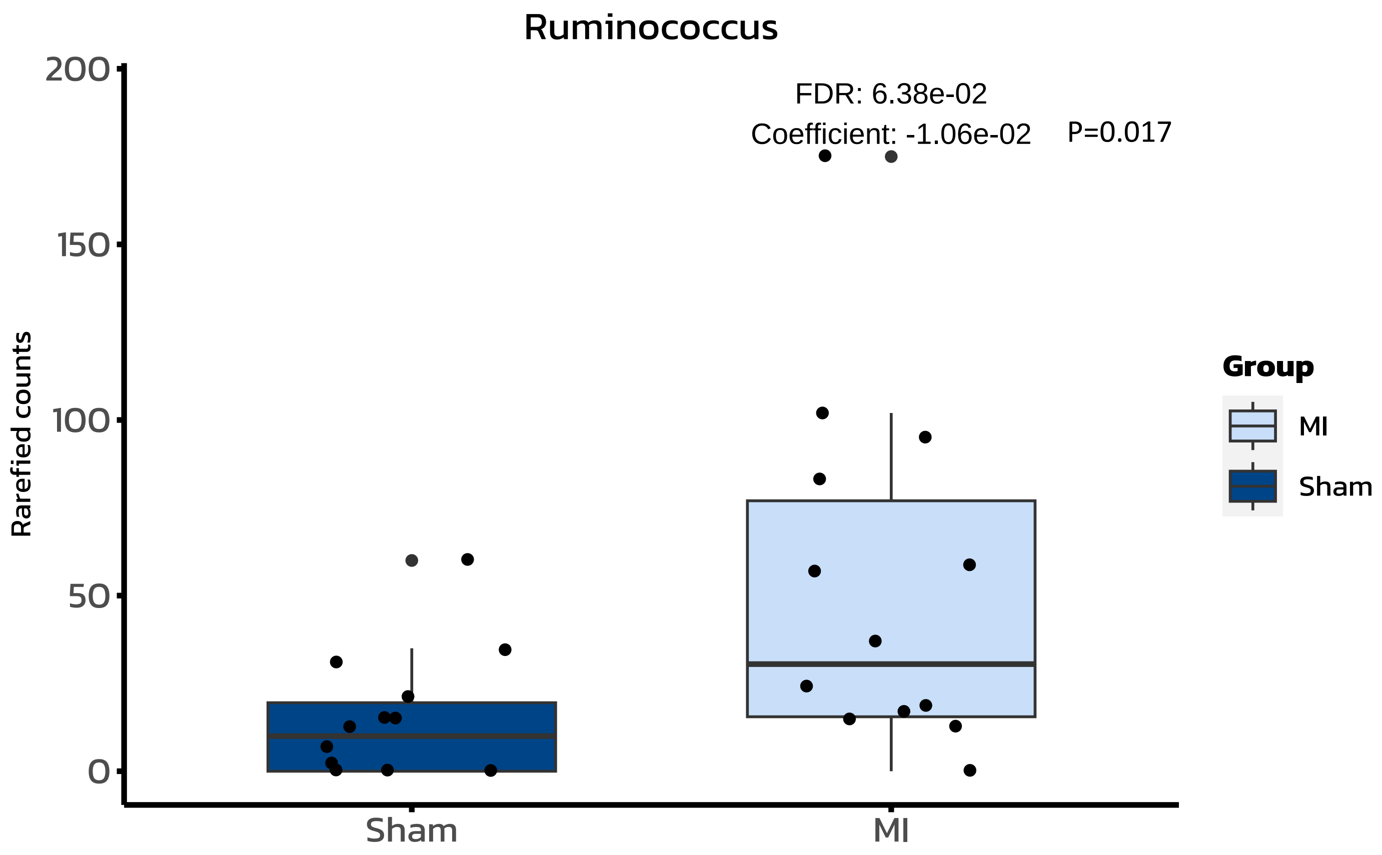
Ruminococcus
FDR: 6.38e-02
Coefficient: -1.06e-02
P=0.017
Rarefied counts
200
150
100
50
0
Sham
MI
Group
MI
Sham

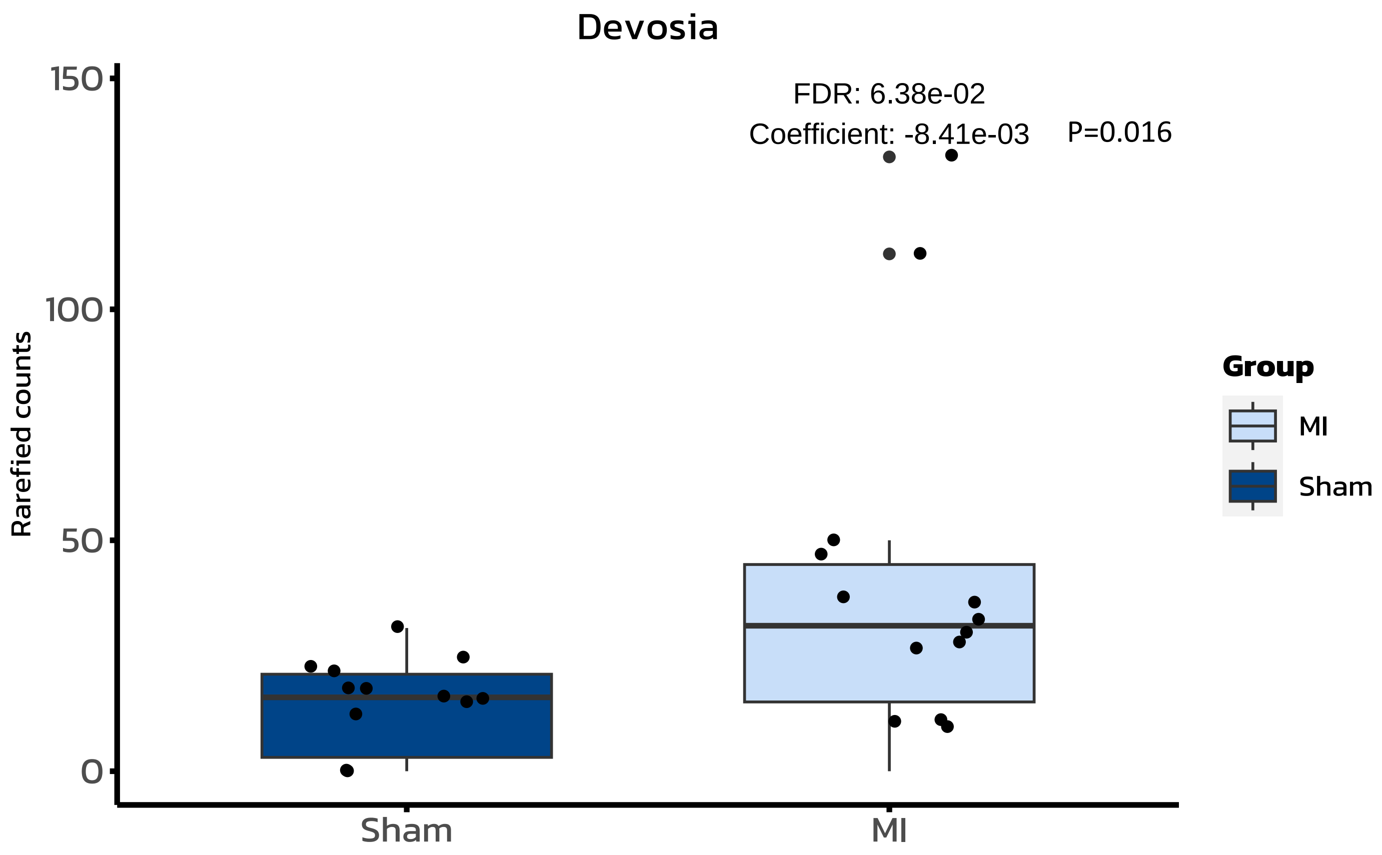
Devosia
FDR: 6.38e-02
Coefficient: -8.41e-03
P=0.016
Rarefied counts
150
100
50
0
Sham
MI
Group
MI
Sham

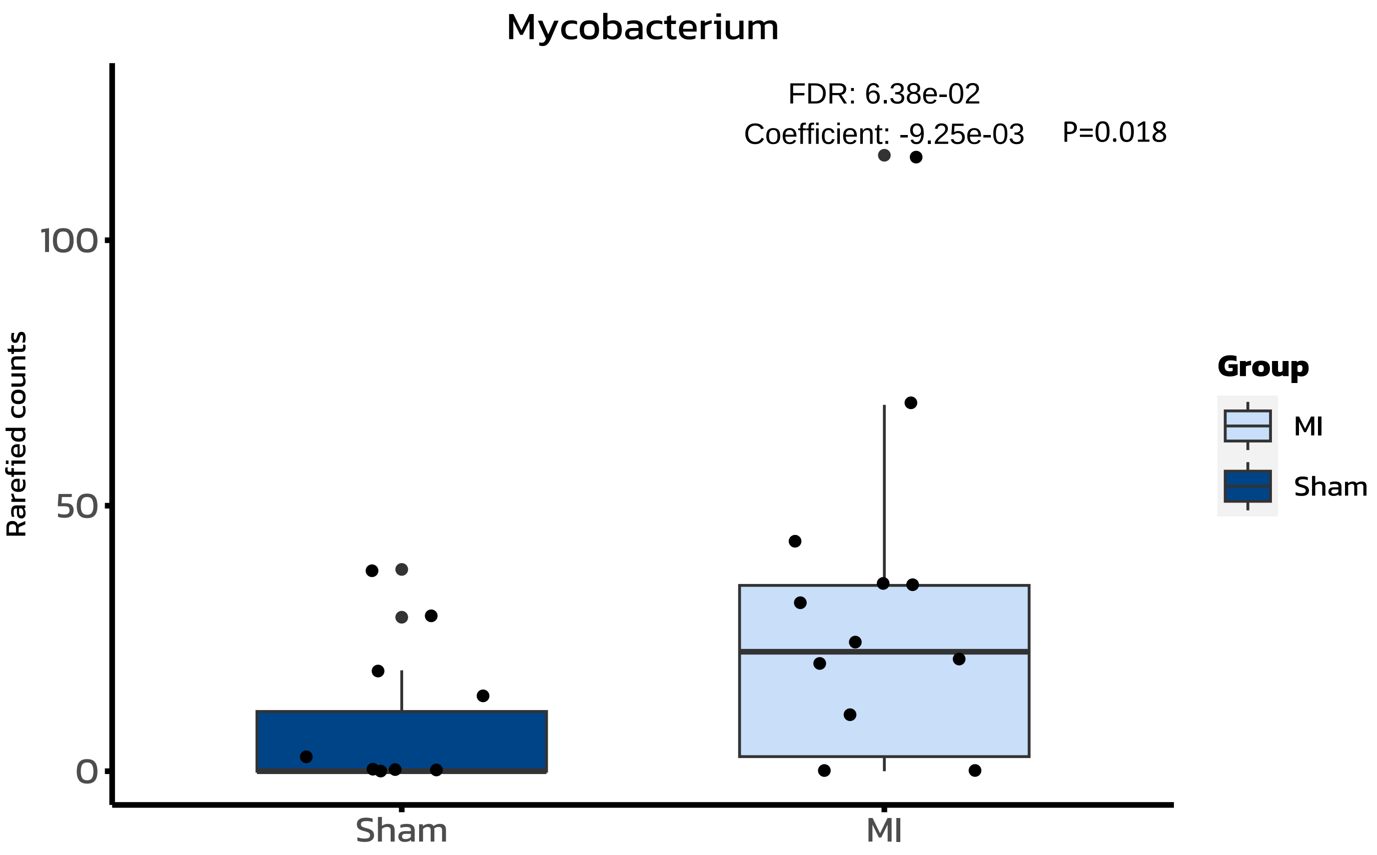
Mycobacterium
FDR: 6.38e-02
Coefficient: -9.25e-03
P=0.018
Rarefied counts
100
50
0
Sham
MI
Group
MI
Sham

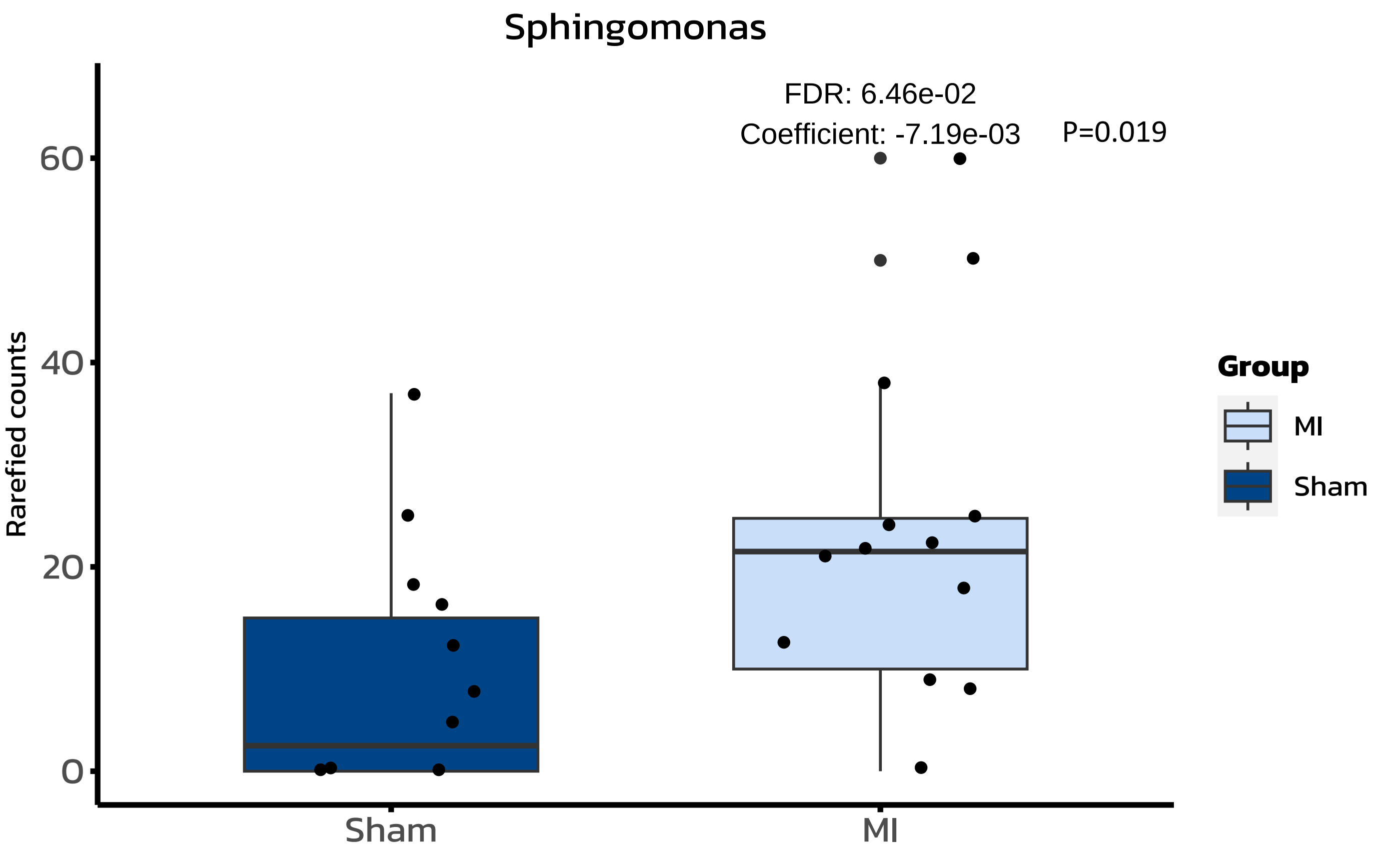

Sphingomonas
FDR: 6.46e-02
Coefficient: -7.19e-03
P=0.019
Rarefied counts
60
40
20
0
Sham
MI
Group
MI
Sham

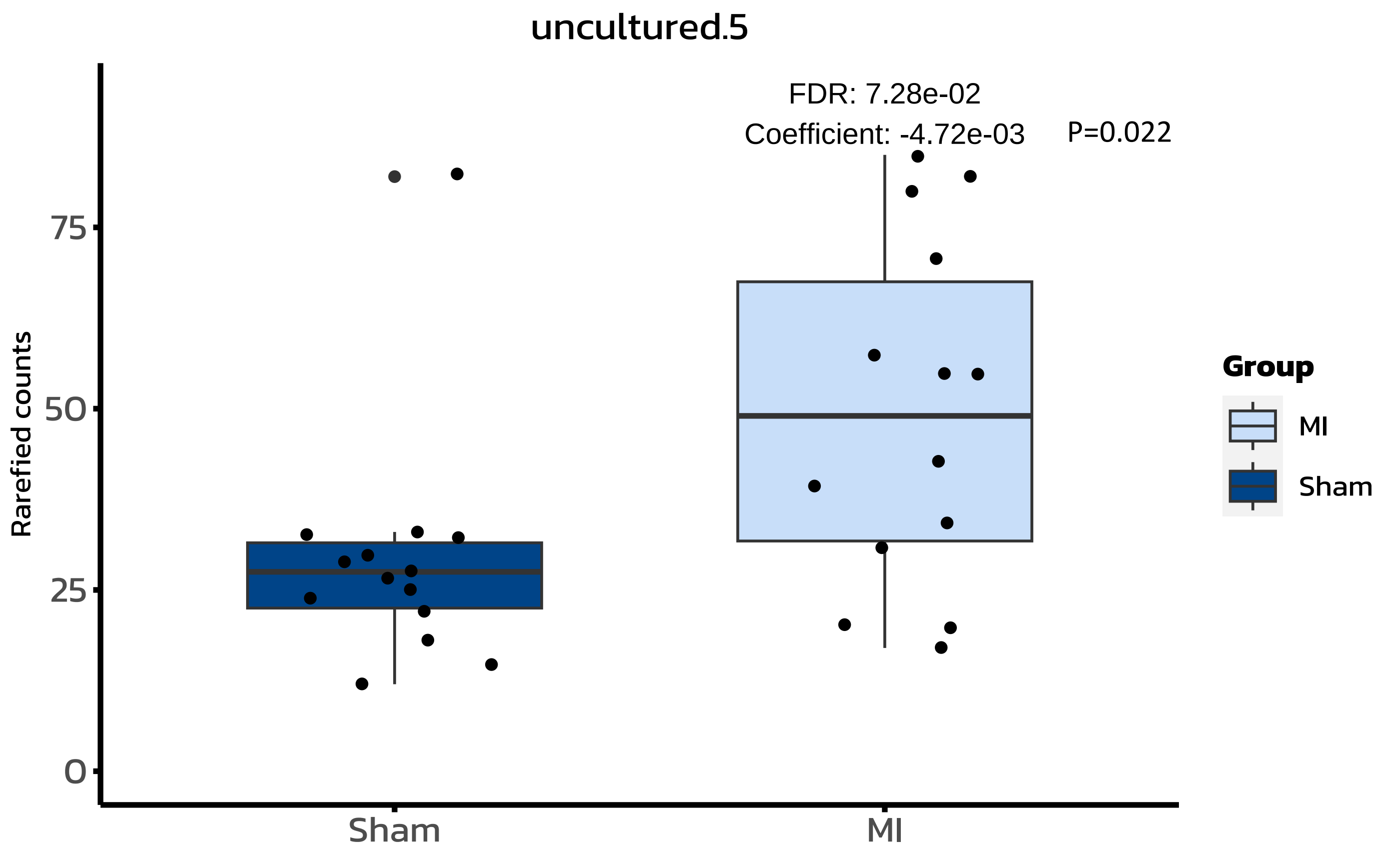
uncultured.5
FDR: 7.28e-02
Coefficient: -4.72e-03
P=0.022
Rarefied counts
75
50
25
0
Sham
MI
Group
MI
Sham

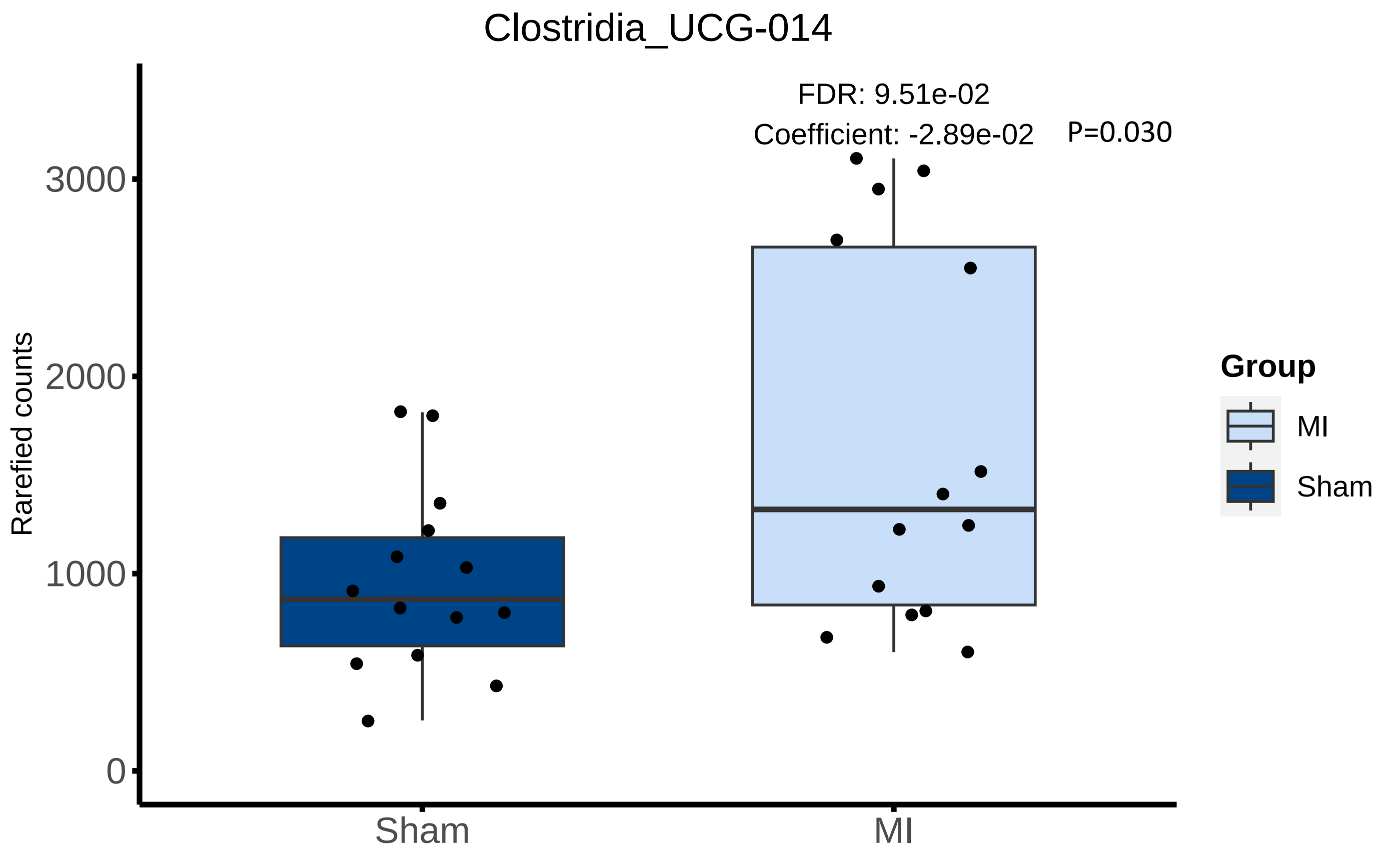

Clostridia_UCG-014
FDR: 9.51e-02
Coefficient: -2.89e-02
P=0.030
Rarefied counts
0
1000
2000
3000
Sham
MI
Group
MI
Sham

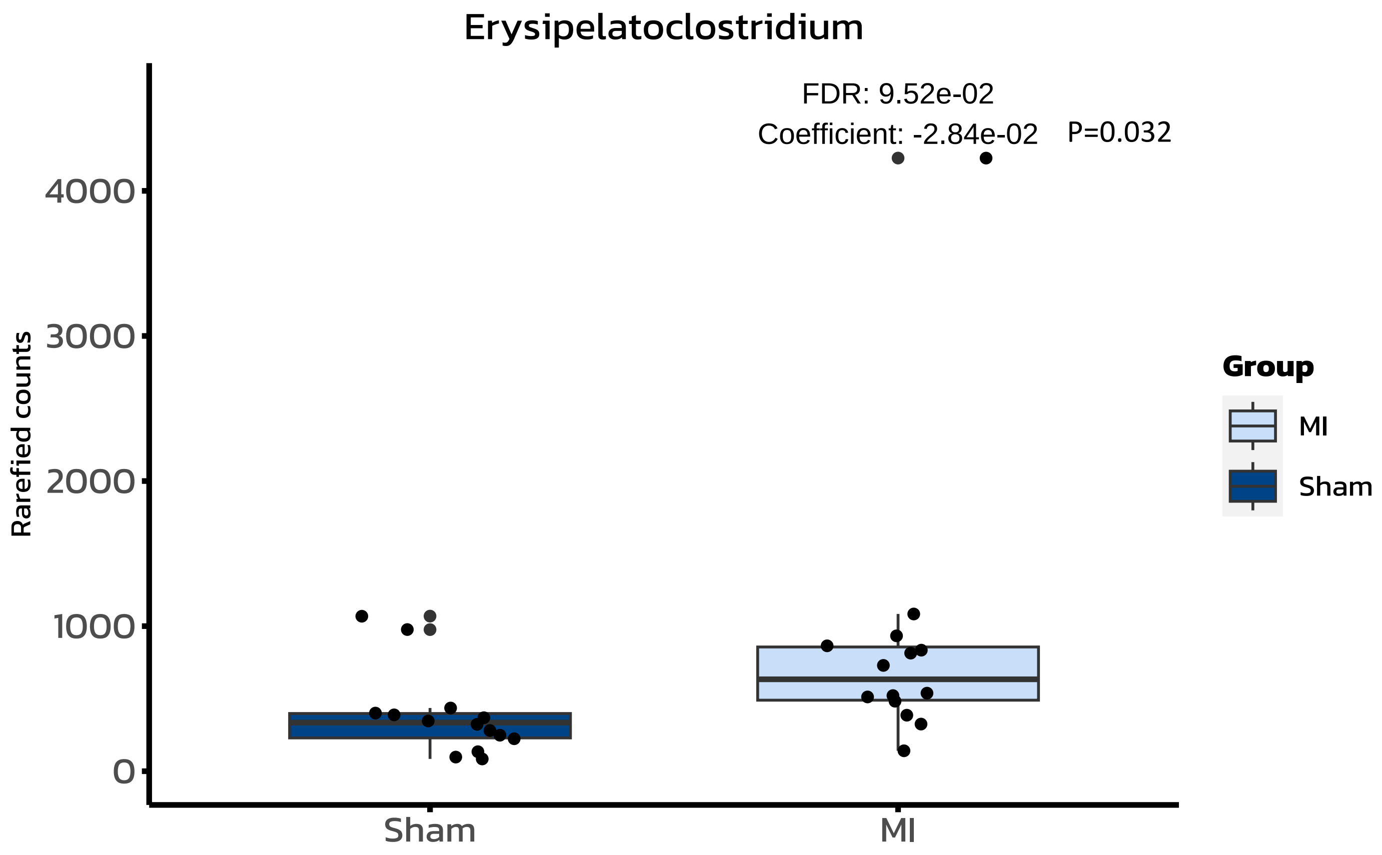
Erysipelatoclostridium
FDR: 9.52e-02
Coefficient: -2.84e-02
P=0.032
Rarefied counts
4000
3000
2000
1000
0
Sham
MI
Group
MI
Sham

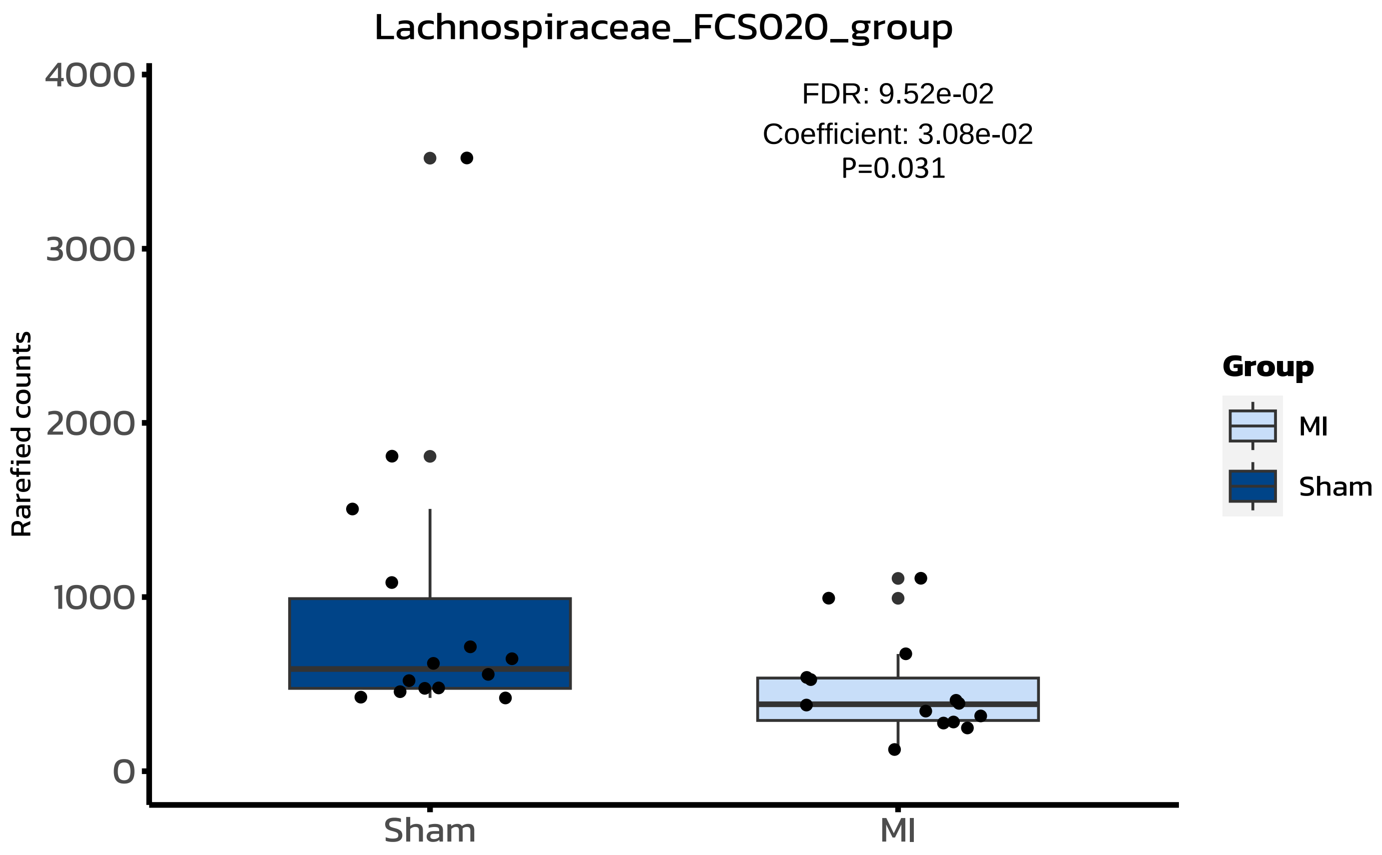
Lachnospiraceae_FCS020_group
FDR: 9.52e-02
Coefficient: 3.08e-02
P=0.031
Rarefied counts
4000
3000
2000
1000
0
Sham
MI
Group
MI
Sham

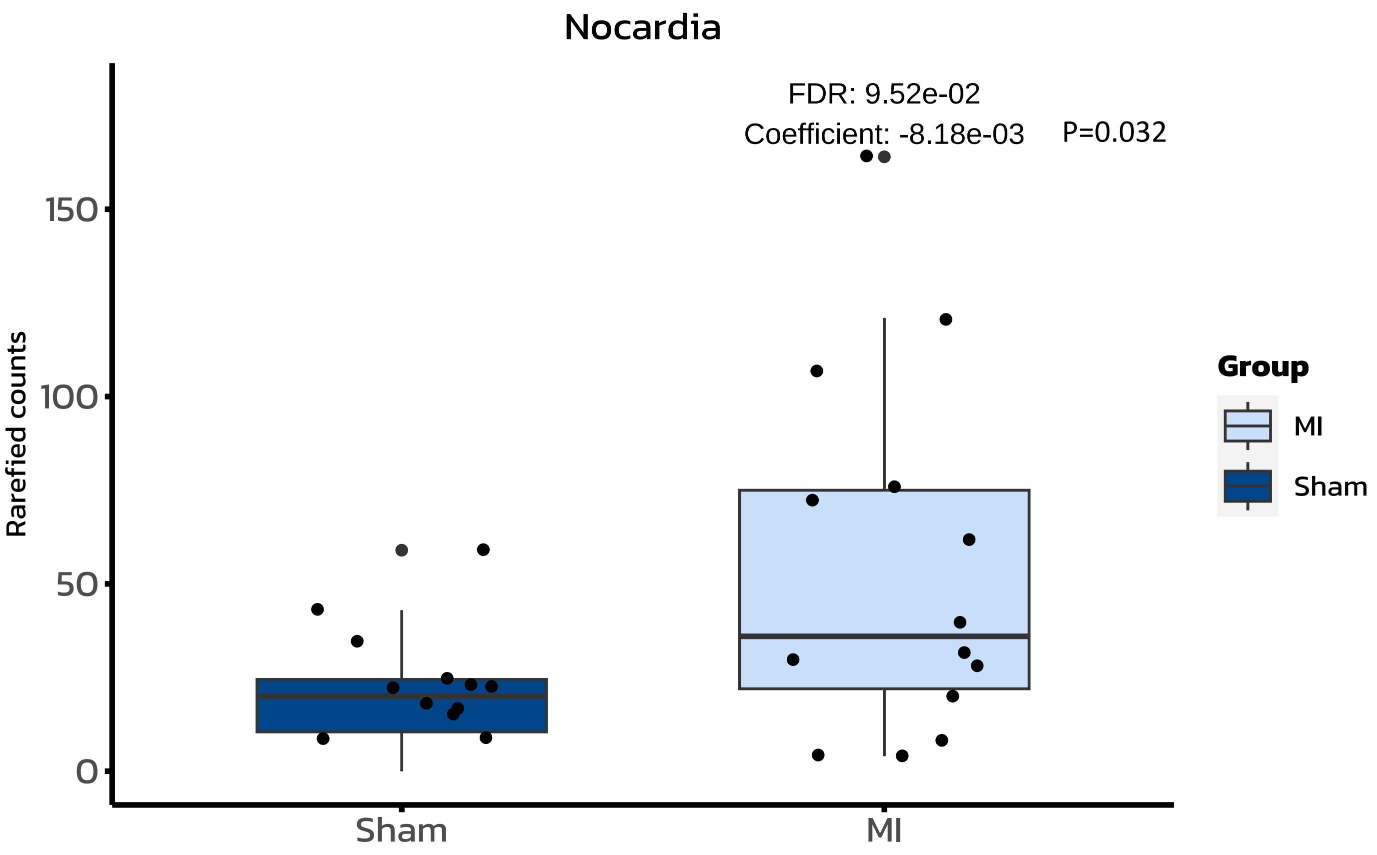
Nocardia
FDR: 9.52e-02
Coefficient: -8.18e-03
P=0.032
Rarefied counts
150
100
50
0
Sham
MI
Group
MI
Sham

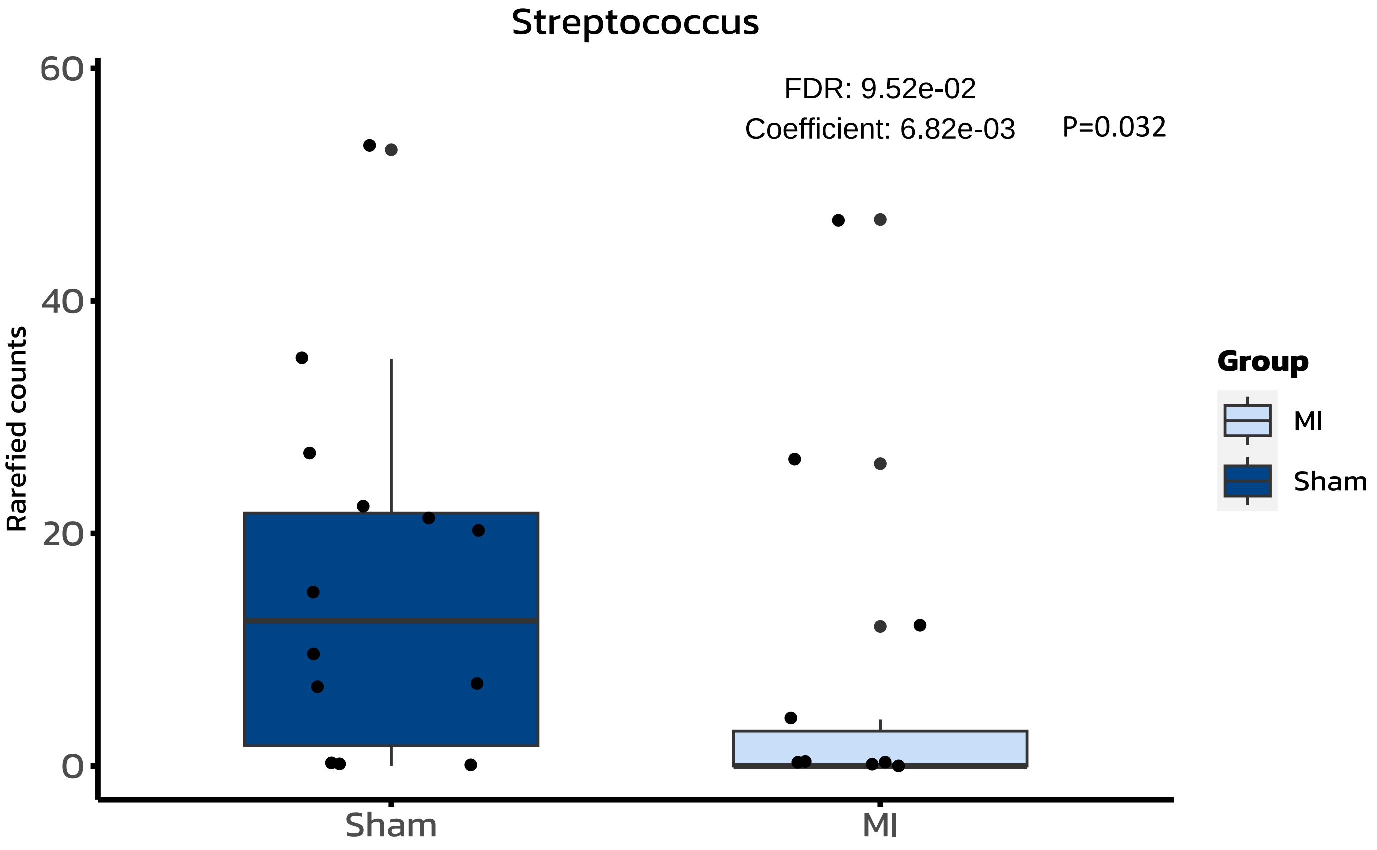
Streptococcus
FDR: 9.52e-02
Coefficient: 6.82e-03
P=0.032
Rarefied counts
60
40
20
0
Sham
MI
Group
MI
Sham

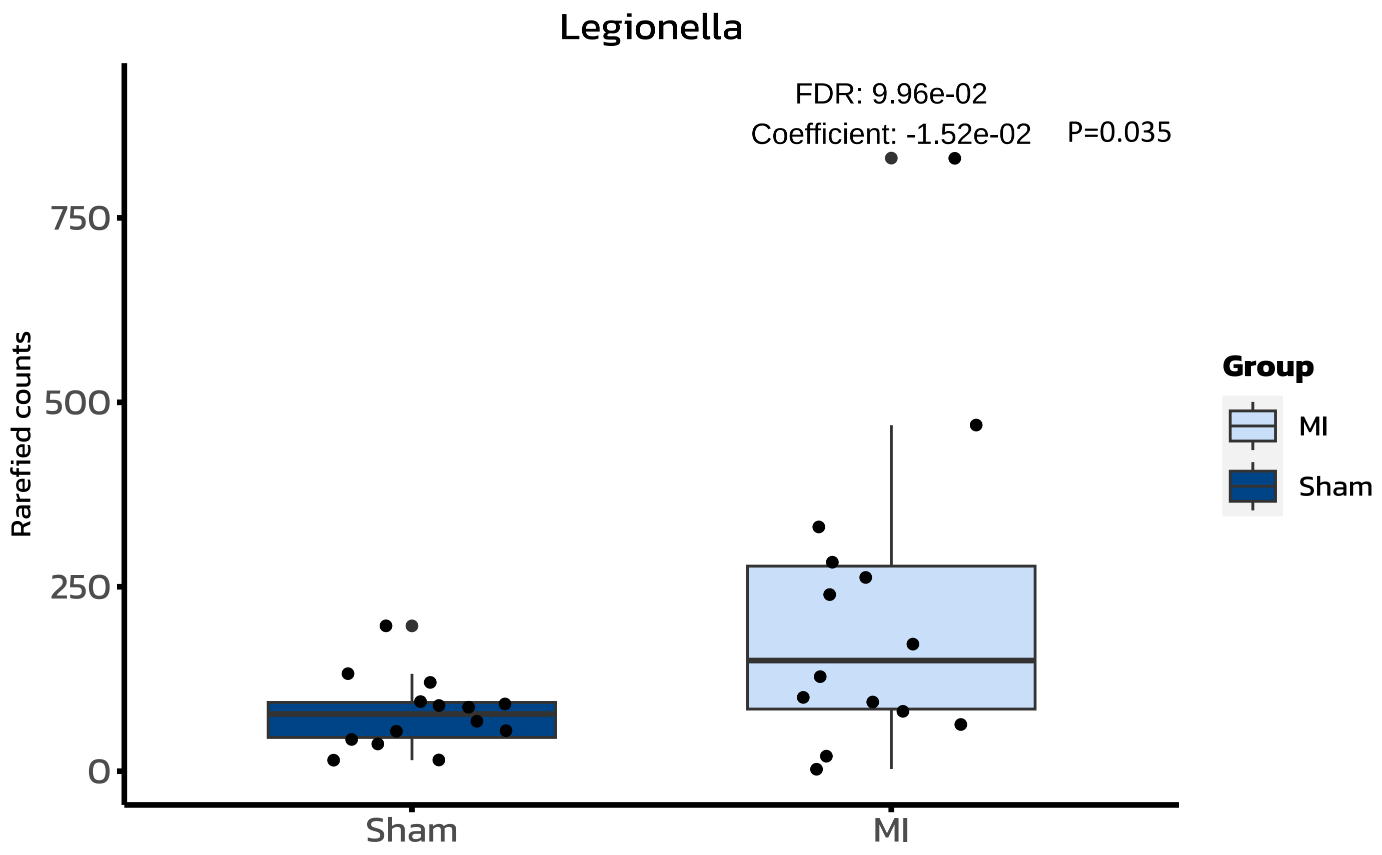
Legionella
FDR: 9.96e-02
Coefficient: -1.52e-02
P=0.035
Rarefied counts
750
500
250
0
Sham
MI
Group
MI
Sham

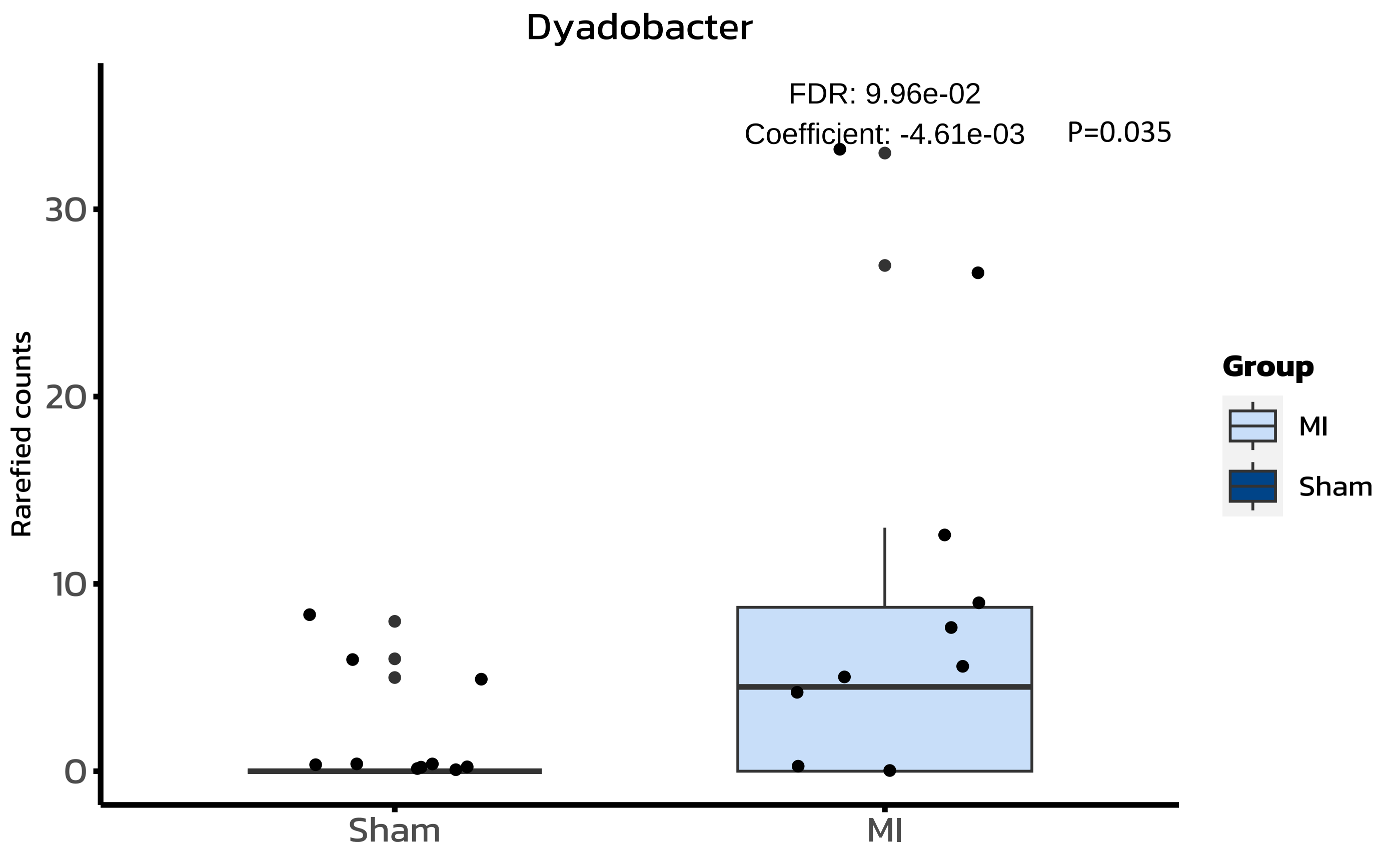
Dyadobacter
FDR: 9.96e-02
Coefficient: -4.61e-03
P=0.035
Rarefied counts
30
20
10
0
Sham
MI
Group
MI
Sham

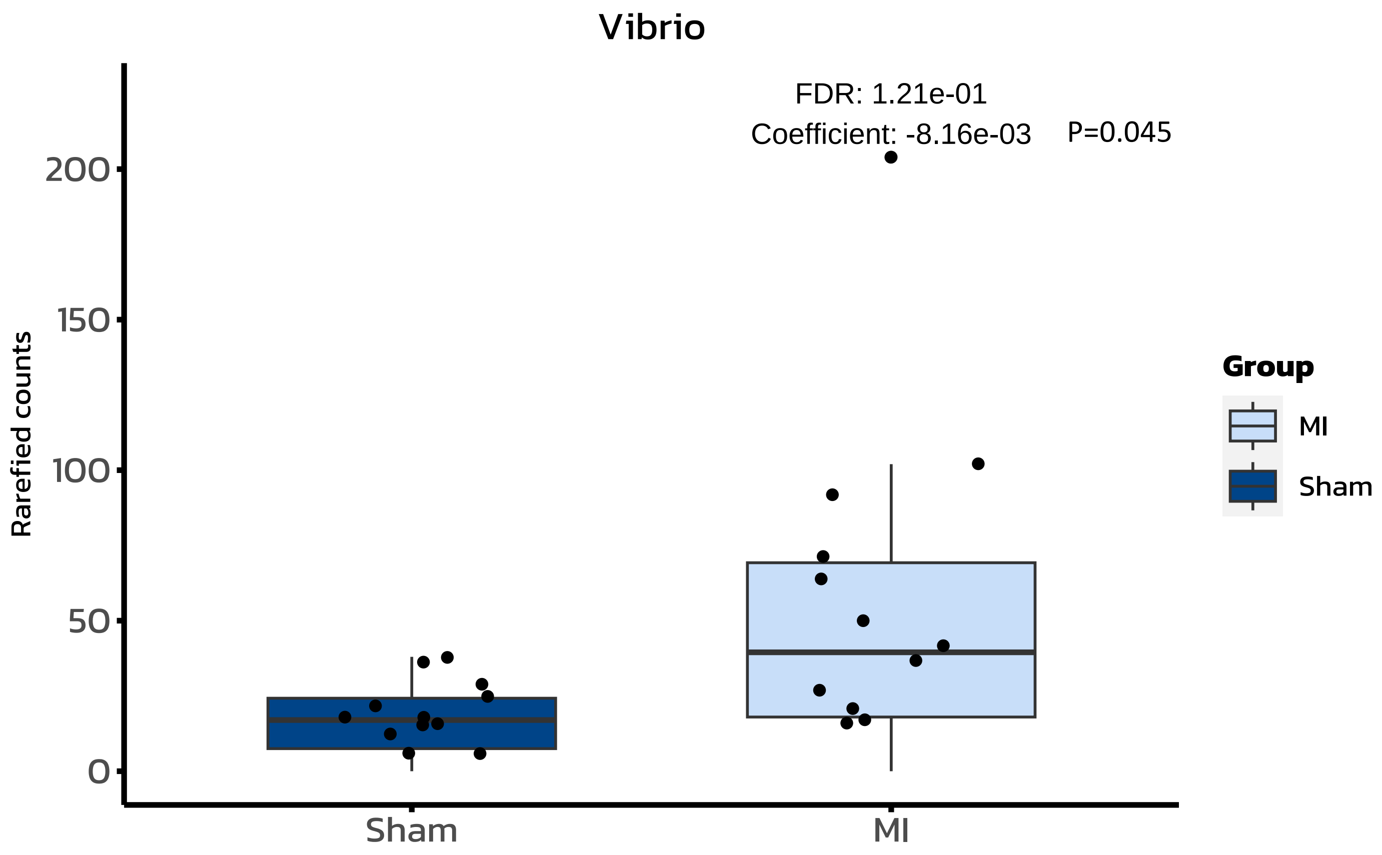
Vibrio
FDR: 1.21e-01
Coefficient: -8.16e-03
P=0.045
Rarefied counts
200
150
100
50
0
Sham
MI
Group
MI
Sham

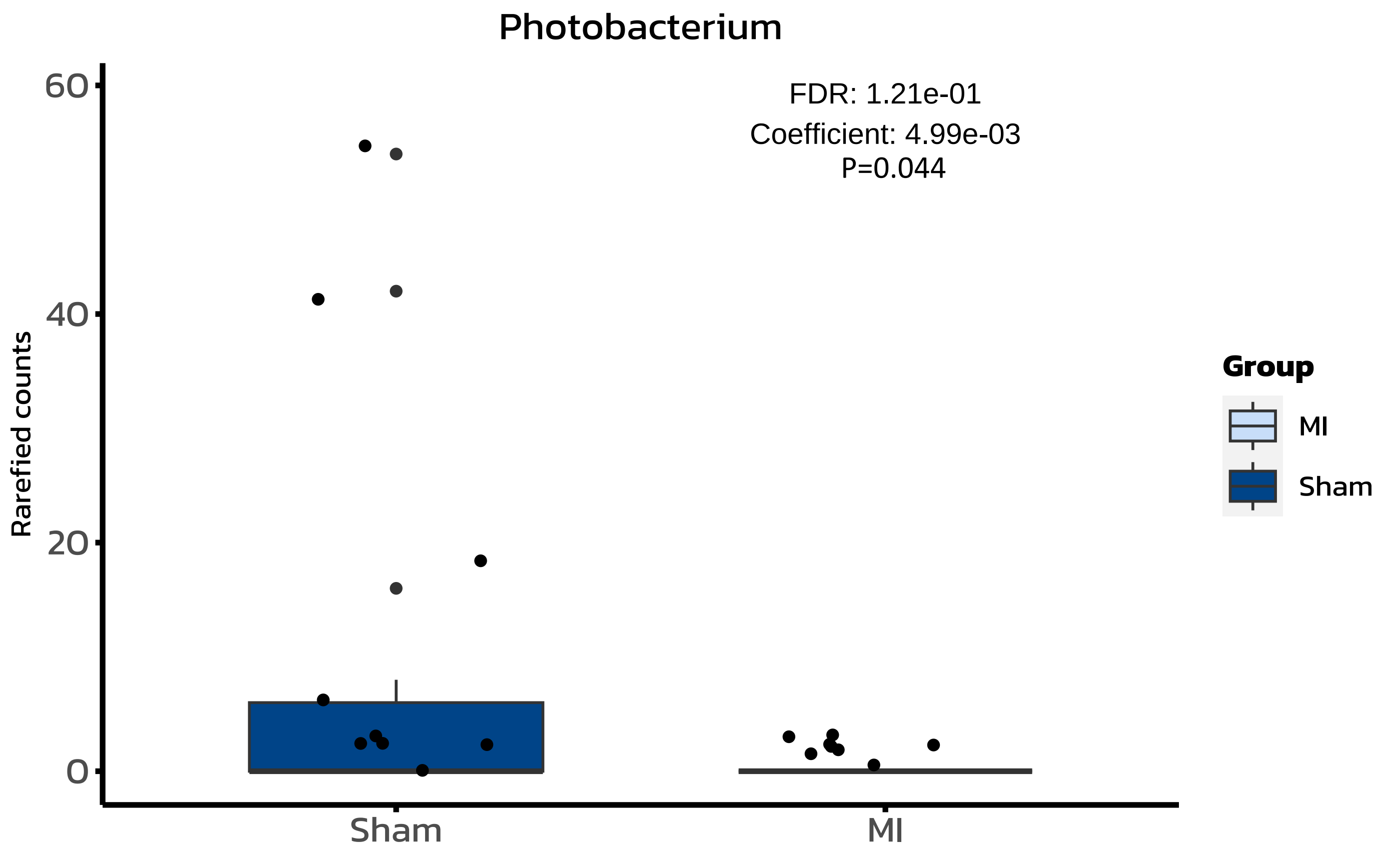
Photobacterium
FDR: 1.21e-01
Coefficient: 4.99e-03
P=0.044
Rarefied counts
60
40
20
0
Sham
MI
Group
MI
Sham

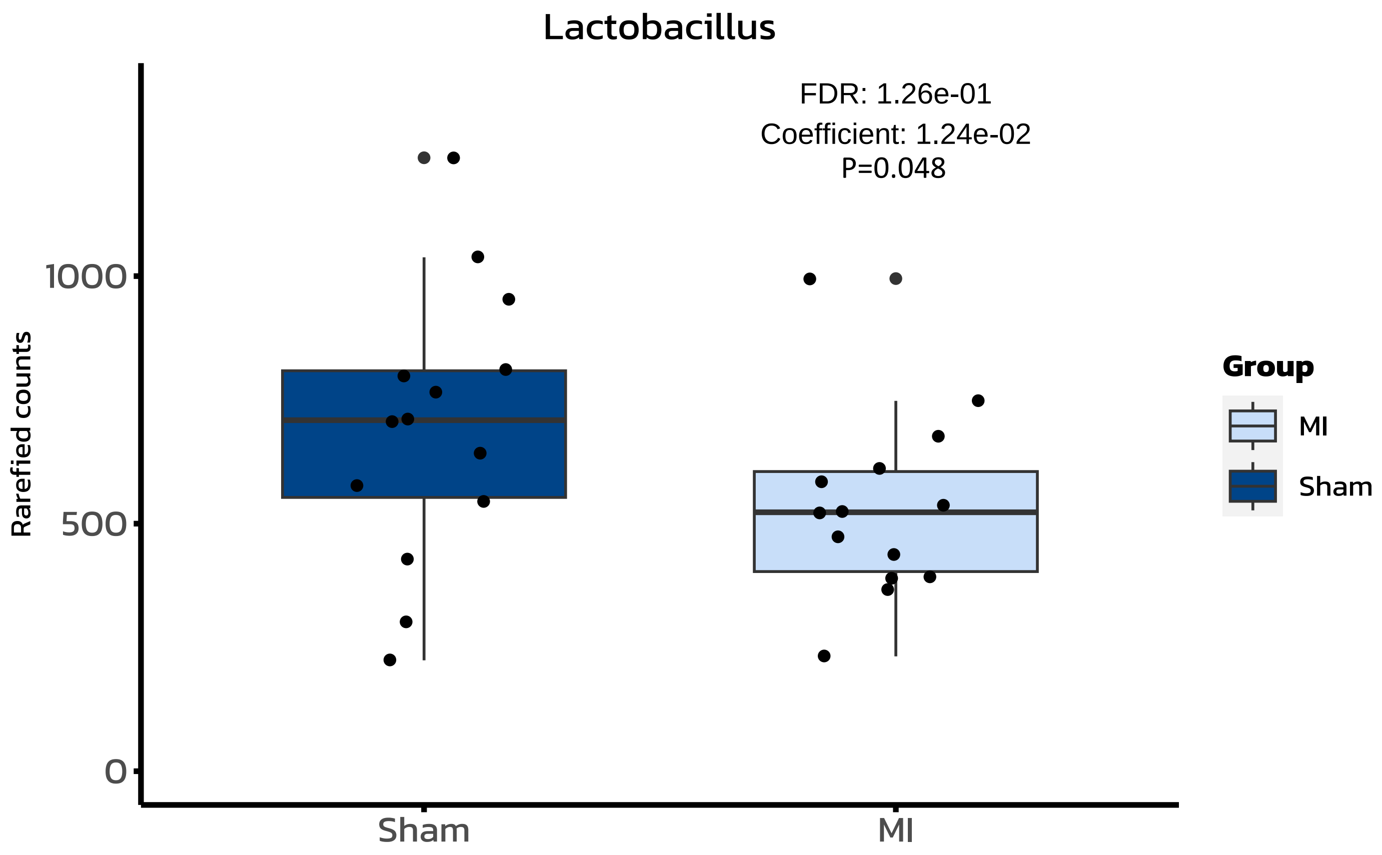
Lactobacillus
FDR: 1.26e-01
Coefficient: 1.24e-02
P=0.048
Rarefied counts
1000
500
0
Sham
MI
Group
MI
Sham
