# Supplementary figures and images for: Heart failure-induced microbial dysbiosis contributes to colonic tumour formation in mice
Source: Cardiovasc Res. 2024 Feb 24;120(6):612–22. doi: 10.1093/cvr/cvae038 (PMC11074794; doi:10.1093/cvr/cvae038)

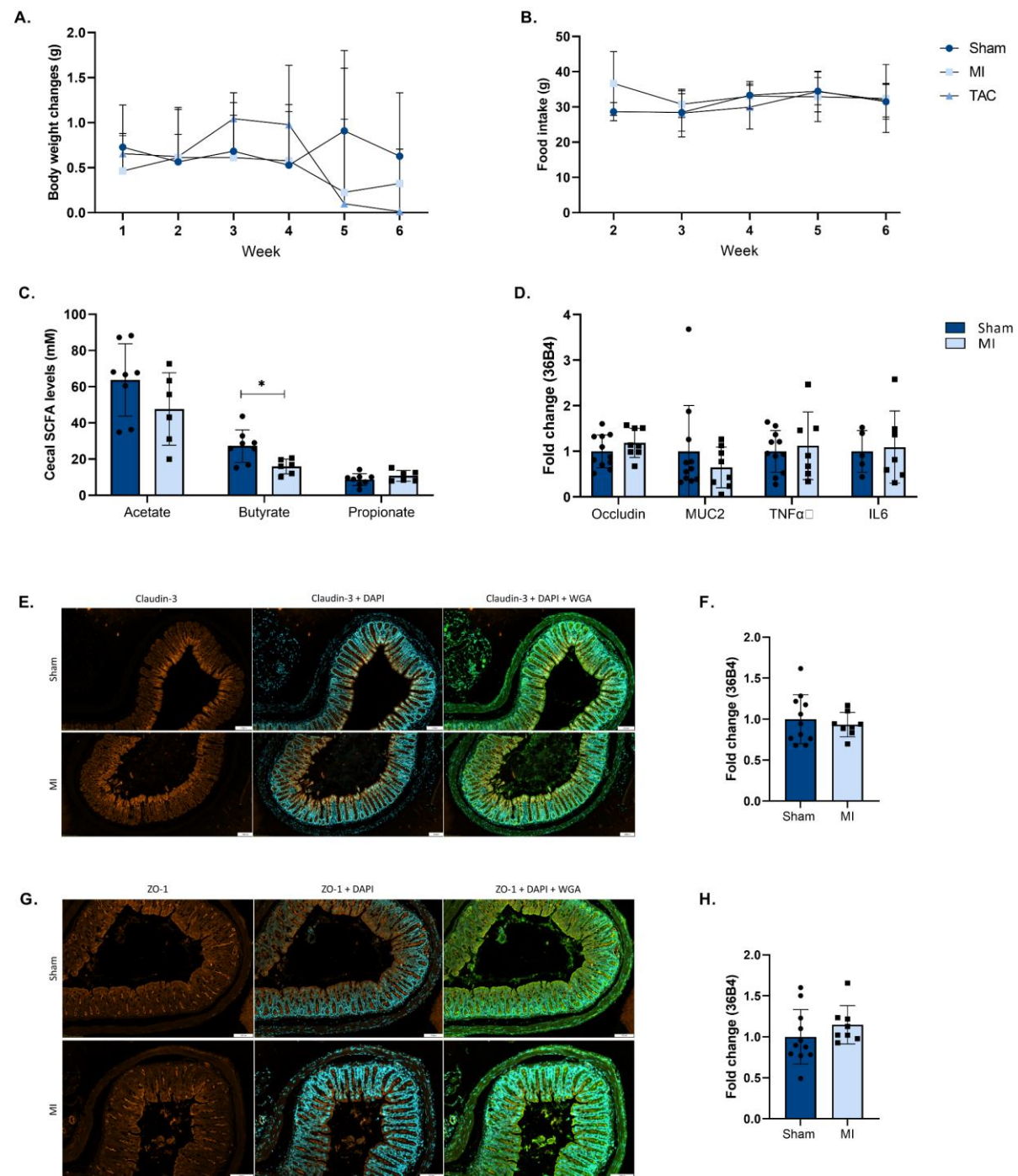

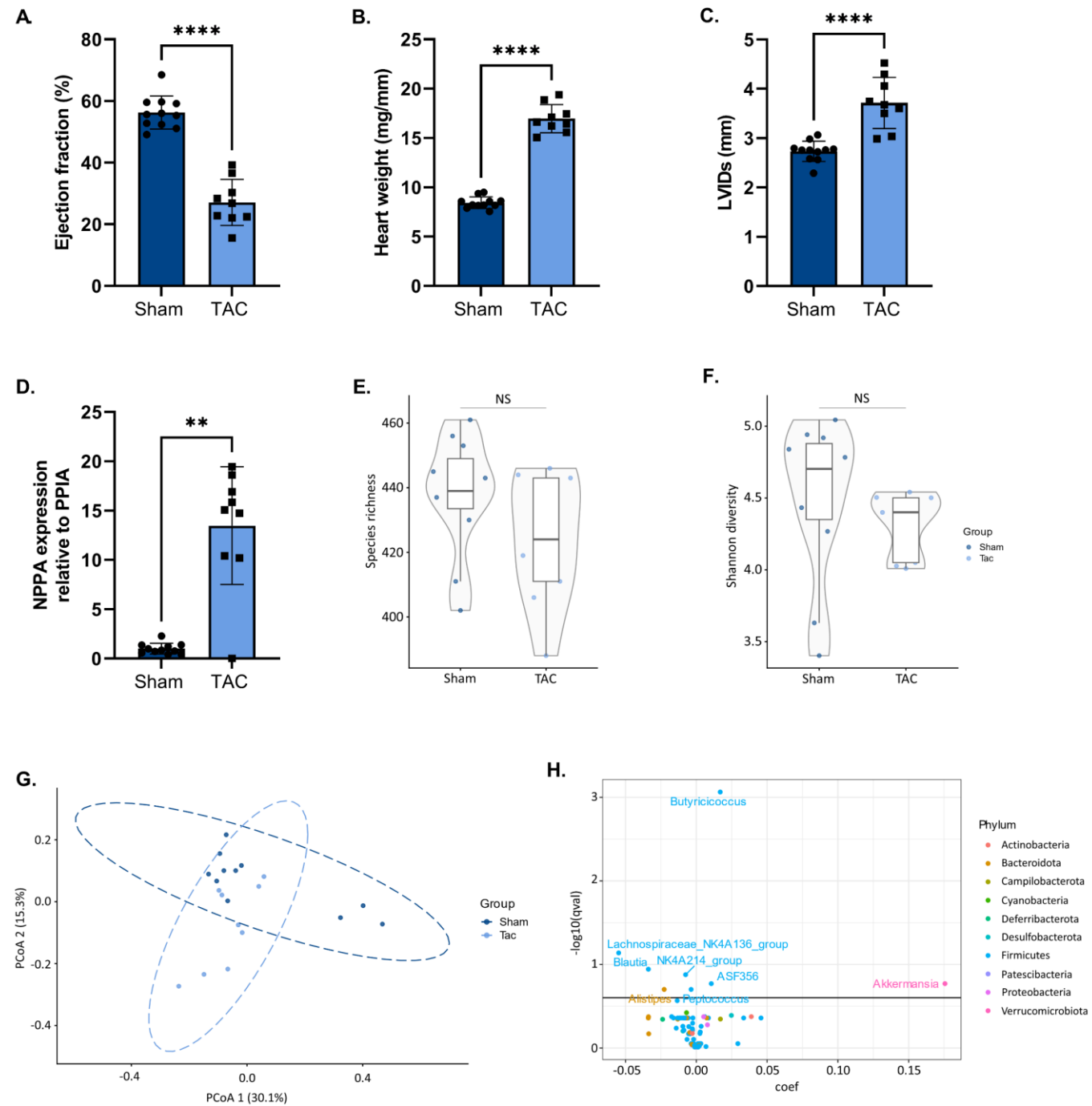

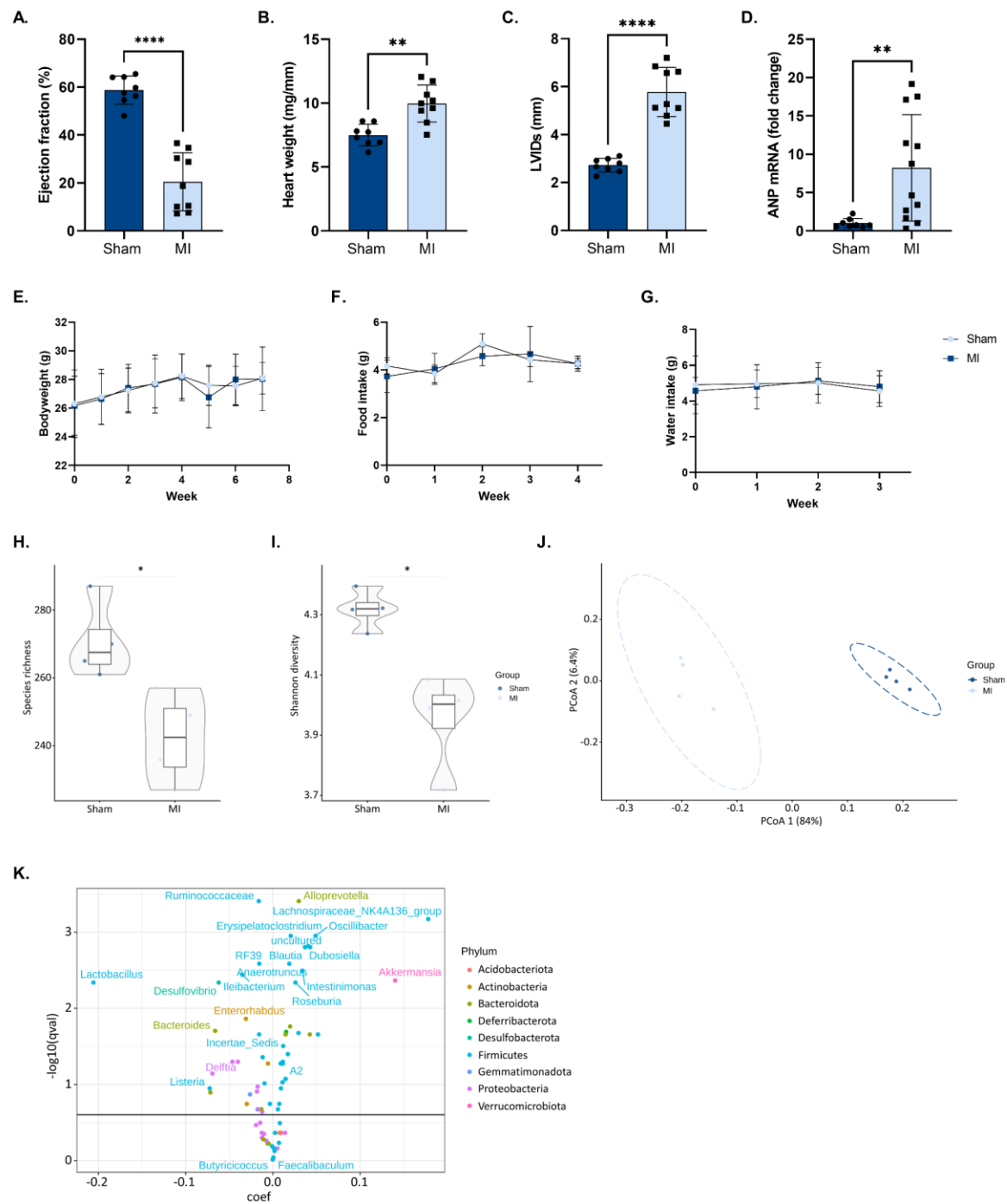

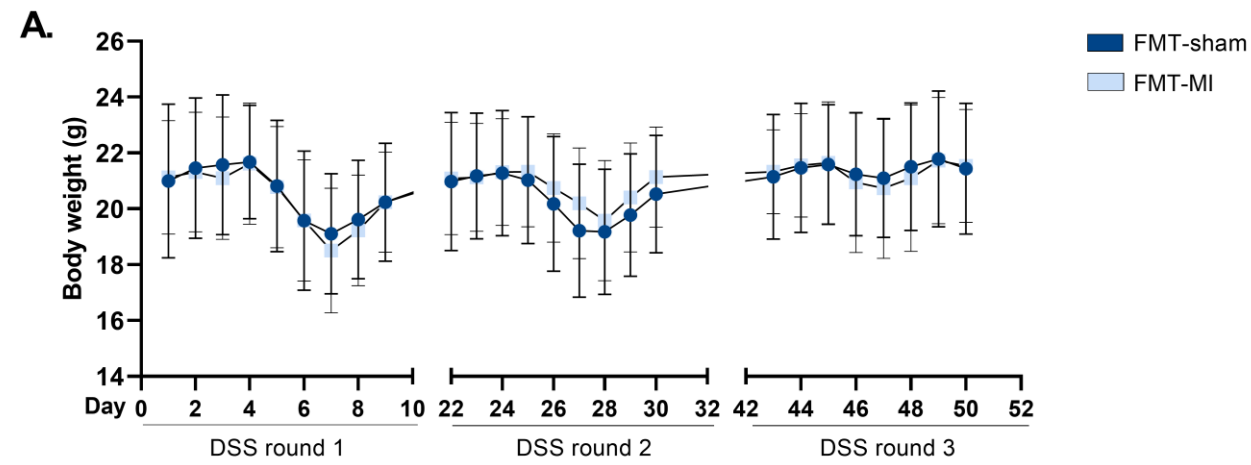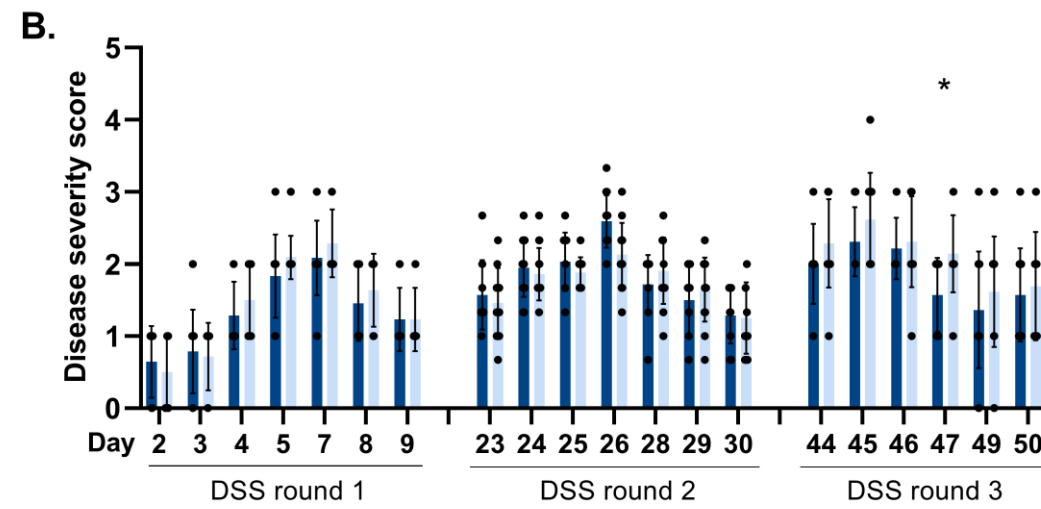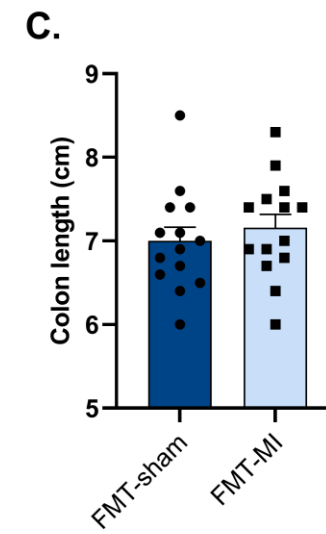

Supplement: cvae038_Supplementary_Data [file cvae038_supplementary_data.zip › Supplemental Figures.pdf]
